# The Small Heat Shock Protein α-Crystallin B Shows Neuroprotective Properties in a Glaucoma Animal Model

**DOI:** 10.3390/ijms18112418

**Published:** 2017-11-14

**Authors:** Fabian Anders, Aiwei Liu, Carolina Mann, Julia Teister, Jasmin Lauzi, Solon Thanos, Franz H. Grus, Norbert Pfeiffer, Verena Prokosch

**Affiliations:** 1 Experimental Ophthalmology, Department of Ophthalmology, University Medical Center of the Johannes Gutenberg–University Mainz, Langenbeckstrasse 1, 55131 Mainz, Germany; fanders@eye-research.org (F.A.); aliu@eye-research.org (A.L.); cmann@students.uni-mainz.de (C.M.); jteister@eye-research.org (J.T.); jasminschweikhard@gmx.de (J.L.); grus@eye-research.org (F.H.G.); norbert.pfeiffer@unimedizin-mainz.de (N.P.); 2 Department of Experimental Ophthalmology, School of Medicine, University of Münster, Albert-Schweitzer-Campus 1, 48149 Münster, Germany; solon@uni-muenster.de

**Keywords:** experimental glaucoma, α-crystallin B, neuroprotection, proteomics

## Abstract

Glaucoma is a neurodegenerative disease that leads to irreversible retinal ganglion cell (RGC) loss and is one of the main causes of blindness worldwide. The pathogenesis of glaucoma remains unclear, and novel approaches for neuroprotective treatments are urgently needed. Previous studies have revealed significant down-regulation of α-crystallin B as an initial reaction to elevated intraocular pressure (IOP), followed by a clear but delayed up-regulation, suggesting that this small heat-shock protein plays a pathophysiological role in the disease. This study analyzed the neuroprotective effect of α-crystallin B in an experimental animal model of glaucoma. Significant IOP elevation induced by episcleral vein cauterization resulted in a considerable impairment of the RGCs and the retinal nerve fiber layer. An intravitreal injection of α-crystallin B at the time of the IOP increase was able to rescue the RGCs, as measured in a functional photopic electroretinogram, retinal nerve fiber layer thickness, and RGC counts. Mass-spectrometry-based proteomics and antibody-microarray measurements indicated that a α-crystallin injection distinctly up-regulated all of the subclasses (α, β, and γ) of the crystallin protein family. The creation of an interactive protein network revealed clear correlations between individual proteins, which showed a regulatory shift resulting from the crystallin injection. The neuroprotective properties of α-crystallin B further demonstrate the potential importance of crystallin proteins in developing therapeutic options for glaucoma.

## 1. Introduction

Glaucoma is a neurodegenerative disorder affecting the optic nerve head that causes the irreversible loss of retinal ganglion cells (RGCs) [1,2]. Glaucoma is one of the leading causes of blindness worldwide [3], and its prevalence is predicted to increase dramatically in the foreseeable future [4]. The mainstay of treatment is based on lowering the intraocular pressure, which is the main risk factor. However, glaucoma may also occur and progress in patients with a normal intraocular pressure (IOP), or successful IOP reduction [5,6,7]. The pathophysiology of glaucoma remains unclear.

Factors additional to the IOP increase, such as lamina cribrosa alterations, ocular tissue stiffness, diminished resilience of RGCs [8,9] vascular defects, reactive oxygen species, high glutamate levels, and overproduction of nitric oxide causing apoptosis, have also been suspected to play important roles in glaucoma [10,11,12,13,14]. However, the definitive pathophysiological mechanisms underlying the course of this disease remain elusive.

In vivo experimental models of glaucoma remain one of the most promising approaches to obtaining further insights into the origin of glaucoma and its pathogenesis and the associated molecular alterations within the retina. A particularly interesting protein family in this context is the crystallins, which are major structural proteins of the lens that have been widely reported as being essential in retinal and also CNS structures. The protein family itself is rather heterogeneous, and comprises three different subgroups: α, β, and γ. Recent studies found shifts in α- and β-crystallin regulation in glaucoma animal trials during different stages of RGC loss [15,16].

α-Crystallins have already been described in various other neurodegenerative diseases, such as Alzheimer’s disease and Parkinson’s disease [17,18], and also in the context of ocular disorders such as diabetic retinopathy and age-related macular degeneration (AMD) [19,20]. Due to their high homology with the small heat shock proteins (sHSP), α-crystallins are believed to act as molecular chaperons in the presence of an external stimulus [21].

Thus, the aim of this study was the measure of α-crystallin B and its potential neuroprotective properties in an experimental animal model of glaucoma with a subsequent analysis of possible alterations in downstream markers in a complex mass-spectrometric (MS) approach.

## 2. Results

### 2.1. IOP Elevation

**Figure 1:**
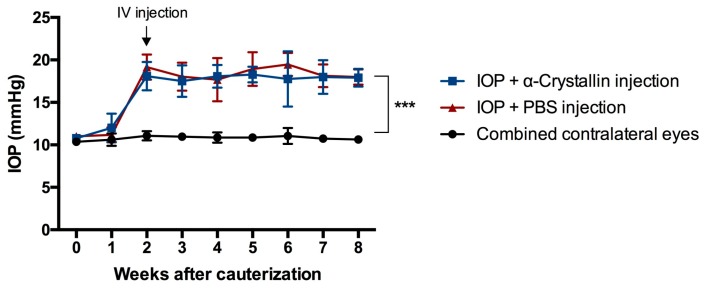
IOP elevation as a result of the episcleral vein cauterization. The intraocular pressure (IOP) of all cauterized eyes rose significantly (*** *p* < 0.001, *n* = 11, mean ± SD, parametric *t*-test) two weeks after the surgical intervention. With rise of the IOP, the intravitreal (IV) injection of α-crystallin B and PBS was performed. No noticeable influence on the IOP was observed due to the injection. The untreated contralateral eyes remained unaffected in terms of IOP changes.

Cauterization of the episcleral veins resulted in a significant elevation of IOP in all of the eyes where the cauterization surgery was performed. The IOP rose from a baseline level of 10.88 ± 0.38 mmHg, to an average level of 17.94 ± 1.72 mmHg in the α-crystallin B injected eyes and an average level of 18.48 ± 1.63 mmHg in the phosphate-buffered saline PBS injected eyes (Figure 1). Neither did the intravitreal injection have an influence on the IOP nor was the IOP of the contralateral eyes influenced by the surgery performed on the left eye of the animals (average IOP contralateral eye: 10.79 ± 0.56 mmHg).

### 2.2. Morphological Changes

**Figure 2:**
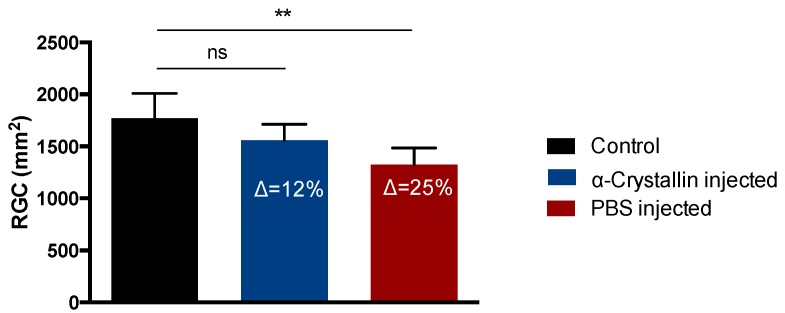
Quantification of RGC density in retinal flat-mounts. Elevation of the IOP resulted in an average loss of ∆ = 12% retinal ganglion cell (RGCs) in the α-crystallin B injected animals compared to the untreated contralateral eyes, while the RGC loss in the PBS-injected animals was about 25% (** *p* < 0.01, ns—not significant, *n* = 11, mean ± SD, one-way ANOVA).

Elevation of the IOP resulted in considerable changes in the RGC density and RNFL thickness (Figure 2). When compared to the RGC density of the contralateral eyes with 1771 ± 238.9 RGC/mm^2^, the α-crystallin B injected eyes lost 12% RGCs (1560 ± 153.6 RGC/mm^2^, not significant) and the PBS injected eyes 25% RGCs (1323 ± 161.1 RGC/mm^2^, *p* < 0.01).

**Figure 3:**
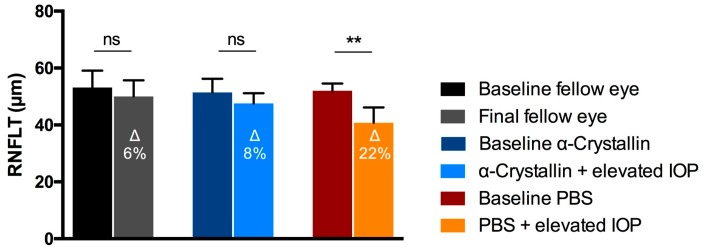
Survey of the retinal nerve fiber layer thickness using optical coherence tomography. Untreated contralateral eyes showed a 6% decrease of the retinal nerve fiber layer thickness (RNFLT) during the time of the study. IOP elevation and α-crystallin B injection effectuated in a loss of ∆ = 8%, while PBS-injected eyes showed a decrease of ∆ = 22% with respect to retinal nerve fiber layer (RNFL) thickness (** *p* < 0.01, ns—not significant, *n* = 5, mean ± SD, one-way ANOVA).

With respect to the RNFLT, there was a 6% decrease between the baseline measurement and the final measurement of the contralateral control eyes (53.18 ± 5.9 µm, 50.00 ± 5.7 µm, not significant), an 8% decrease within the crystallin-injected group (51.40 ± 4.8 µm, 47.60 ± 3.6 µm, not significant), and a 22% reduction of the RNFLT in the PBS-injected group (52.00 ± 2.6 µm, 40.75 ± 5.4 µm, *p* < 0.01) (Figure 3).

### 2.3. Functional Changes

**Figure 4:**
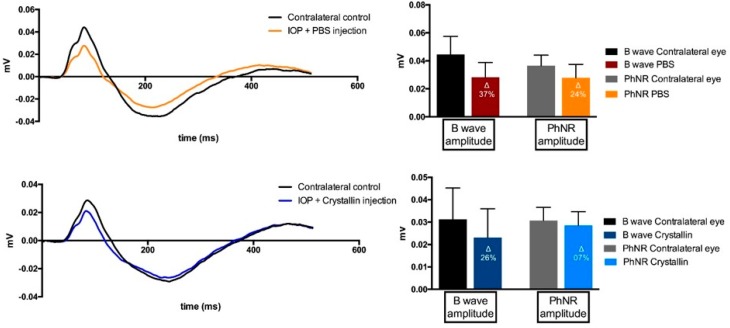
Pattern of the ganzfeld photopic electroretinogram analysis. ERG results showed considerable aberrances in the B-wave and photopic negative response (PhNR) between contralateral control eyes and experimental glaucoma eyes. However, the deviance to the respective control eyes seems much higher in PBS-injected eyes than in α-crystallin B injected eyes (*n* = 11, mean ± SD). The millivolt decrease is defined in percentaged change (∆ = % change).

The Ganzfeld Photopic Electroretinogram (ERG) showed considerable changes in the B-wave and the photopic negative response (PhNR) between the ERG patterns of the contralateral eyes when compared to the eyes with elevated IOP (Figure 4). Nevertheless, differences of the B-wave and PhNR amplitudes were more distinct in the PBS-injected animals as compared to the respective contralateral eyes (B-wave: Δ = 37%, PhNR: Δ = 24%) than was the case in the crystallin-injected group (B-wave: Δ = 26%, PhNR: Δ = 7%).

### 2.4. Proteomic Analysis

**Figure 5:**
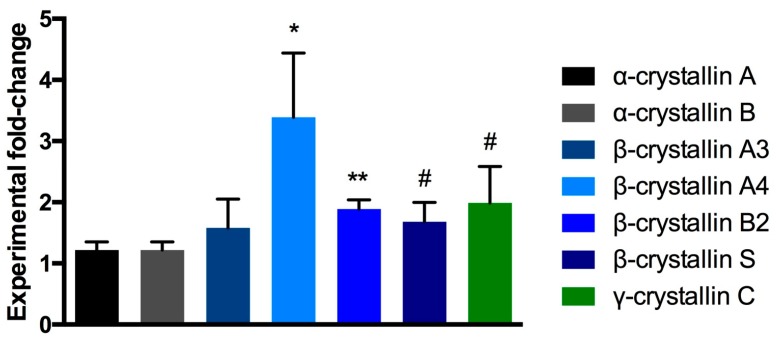
MS fold-change analysis of crystallin protein family members. Injection of α-crystallin B induced moderate to distinct up-regulation of α-, β-, and γ-crystallins in the retina when compared to PBS-injected retinal protein levels (** *p* < 0.01, * *p* < 0.05, ^#^ *p* < 0.1, unpaired parametric *t*-test, *n* = 4 per exp. group, mean ± SEM).

A total number of 1765 retinal proteins could be identified and quantified, whereas only a small proportion of 5% (87 proteins) showed an up- or down-regulation of 1.5-fold or more when compared to the respective control group. However, mass-spectrometric measurements identified higher levels of crystallin proteins of all the subtypes α, β, and γ in the α-crystallin B injected samples as compared to the PBS-injected controls (Figure 5).

**Figure 6:**
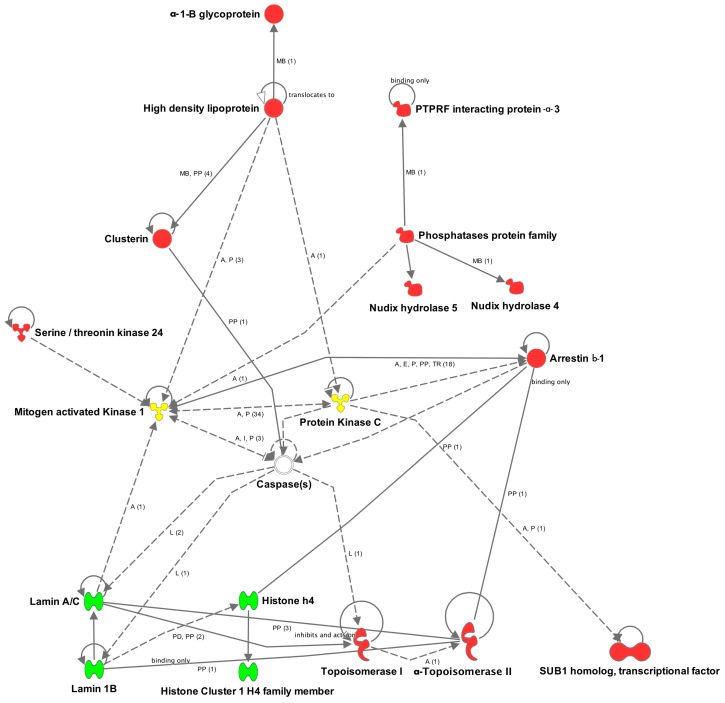
Ingenuity Pathway Analysis interactive protein network of highly up- or down-regulated retinal proteins. Up-regulated proteins (red) are located in the extracellular space, plasma membrane, cytoplasm, as well as in the nucleus. Down-regulated proteins in this network are exclusively located in the nucleus (green). The mitogen-activated protein kinase 1, protein kinase C (yellow) and caspase proteins show considerable interaction with a variety of displayed proteins with altered regulation levels. Legend: A = activation; L = proteolysis; P = phosphorylation/dephosphorylation; PP = protein–protein binding; I = inhibition; E = expression; PD = protein–DNA binding; TR = translocation; MB = group/complex membership; solid arrow = direct interaction; dashed arrow = indirect interaction; (count) = number of scientific references on the respective relation; if not indicated differently in the figure, the arrow head indicates “acts on”.

Creation of an interactive protein network revealed various relations between retinal proteins, identified as up-regulated (red) and down-regulated (green) through all of the cellular layers (Figure 6). Mitogen-activated protein kinase 1 and protein kinase C (yellow), which were also found in the MS data, but without a noticeable change with respect to the relative level, interact with a multitude of the displayed proteins. Caspases could not be identified within the MS measurements, but were added to the network by the IPA^®^ software, since they interact with numerous regulated nucleus proteins.

**Figure 7:**
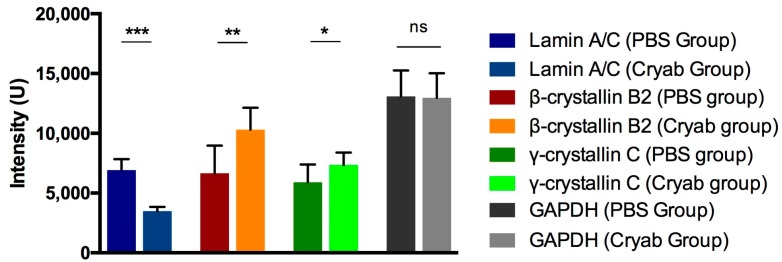
Antibody microarray detection of specifically altered retinal proteins. The protein regulations, detected in the MS measurements, could be validated with antibody microarray for lamin A/C, β-crystallin B2 and γ-crystallin C, using GAPDH for loading control purposes. Lamin A/C levels in the PBS group were significantly higher than in the crystallin-injected (cryab) group, while the levels of β-crystallin B2 and γ-crystallin C were found higher in the cryab group (* *p* < 0.05, ** *p* < 0.01, *** *p* < 0.001, ns—not significant, *n* = 9 per exp. group, mean ± SD, one-way ANOVA).

The down-regulation of the nucleus protein lamin A/C as a reaction to the α-crystallin B injection was further validated by antibody microarray detection and could confirm the previous obtained MS-based results (Figure 7). Measured intensities indicated a two-fold increase of lamin A/C in the retinal protein-mix of PBS-injected eyes when compared to the α-crystallin B injected group (*p* < 0.001).

## 3. Discussion

The data obtained in this study provide further evidence for the neuroprotective effects of crystallin proteins and a potential basis for the development of novel translational glaucoma therapies. The glaucoma model that was constructed in Sprague–Dawley rats led to significant neurodegenerative effects, such as the loss of RGCs, a thinning of the retinal nerve fiber layer (RNFL), and functional alterations within the ERG pattern. However, the injection of α-crystallin B prior to the IOP elevation was able to markedly mitigate these neurodegenerative processes.

The neuronal damage that was induced by occluding episcleral veins and the subsequent elevation of the IOP were highly consistent with the results obtained in previous comparable glaucoma studies [15,20,22]. Regarding the ERG, a low signal for the PhNR amplitude was expected, given the significant loss of RGCs [23], but the changed B-wave amplitude indicated that other retinal segments had also been damaged. However, comparable results have already found in glaucoma models in rats and mice [24,25]. As Nork et al. reported in 2000, chronically elevated IOP can also affect photoreceptor cells, which could explain the observed B-wave changes [26]. Also, Hernandez et al. described molecular changes in the inner retina during different periods of elevated IOP, which is a clear sign that structures additional to RGCs are functionally altered in experimental glaucoma models [27].

At both the morphological and functional levels, the injection of α-crystallin B induced distinct neuroprotective effects in glaucomatous eyes when compared to the glaucomatous eyes that were injected with PBS as controls. No significant difference in RGC density was observable between untreated and crystallin-injected eyes. Also, the decrease in RNFL was almost identical to that in the contralateral eyes. The PhNR amplitudes in the ERG pattern only differed slightly between the crystallin-injected and the untreated contralateral eyes.

Protective effects of α-crystallins have already been reported for acute inflammation, diabetic retinopathy, multiple sclerosis, and UVA-induced photoreceptor apoptosis [28,29,30,31]. Doss et al. questioned the role of α-crystallins in primary open-angle glaucoma already back in 1998 [32]. Several studies have demonstrated neuroprotective properties of crystallins in RGC survival, and even neuroregenerative effects have been attributed to this particular protein group based on findings in experimental glaucoma models [33,34,35,36]. However, the precise mode of action of these protective effects remains unclear. Fischer et al. reported crystallin-mediated astrocyte-derived CNTF as the main reason for the protective effects of crystallins in the retina, while other authors have described the indirect inhibition of c-Jun N-terminal kinases and double-stranded RNA-dependent protein kinases [33,37,38]. To the best of our knowledge, the present study is the first to provide indications that the injection of a certain member of the crystallin family can stimulate the expression of numerous crystallin proteins covering all of the subclasses. The levels of α-, β-, and γ-crystallins were higher in the retina of eyes injected with α-crystallin B than in the corresponding PBS-injected controls; however, it should be noted that the intravitreal injections were applied almost two months prior to the animals being sacrificed. Anyhow, it is noticeable that specifically the relative protein level of α-crystallin B was only moderately higher with a fold-change of 1.2. Injected α-crystallin is certainly expected to be metabolized over time. Further MS analyses and the bioinformatics-assisted creation of a protein network revealed distinct regulatory changes of retinal proteins in the extracellular space, plasma membrane, cytoplasm, and nucleus. Mitogen-activated protein kinase 1 and protein kinase C appeared to interact strongly with the identified altered proteins, but were themselves found at levels equal to those in the controls.

Clusterin, which is an extracellular protein that is located in the cytoplasm that has been implicated in processes, such as cell-cycle control and apoptosis, was found to be up-regulated in the crystallin-injected retinal tissues. Clusterin functions as a chaperone and was described to be neuroprotective in the context of glaucoma in the interplay between interleukin-6 (IL-6), vascular endothelial growth factor (VEGF), and hypoxia-1α [39]. Caspases, which have a direct binding relation to clusterin, are known to cleave lamin proteins and topoisomerase I in the nucleus [40,41]. Those proteins directly influence cell viability via their effects on cellular functioning and cell-death signaling. Lamin A/C and lamin 1B were found distinctly down-regulated with fold-change ratios of 0.29 and 0.28 due to the crystallin-injection. Decreased levels of lamin A/C were further confirmed in microarray analyses. Lamin A/C was previously identified as an early marker in experimental glaucoma [42], being found to be markedly up-regulated in response to short-term IOP elevation or initial neuronal impairment. The injection of α-crystallin B may counterbalance the increase of lamins in this connection. Lamin proteins are known to have a direct association to cellular apoptosis [43], acting on previous cleavage by caspases [44,45]. The down-regulation of lamins may represent a direct link to the neuroprotective effects on RGCs, and their axons induced by the intravitreal injection of α-crystallin B. This hypothesis is further supported by Adhikari et al. showing the colocalization of α-crystallin B and lamin A/C in the nucleus of cells under stressful conditions [46].

α-1B glycoprotein, the transcriptional repressor CTCF, and the neurofilament light polypeptide, which MS revealed were also altered, but which were not included in the displayed protein network, have previously been reported to be associated with glaucoma [47,48,49]. The altered proteins plectin and filamin-C are both reportedly related to α-crystallin B in the context of neurodegeneration and myofibrillar degeneration [50,51].

It seems obvious from the data obtained in this study, combined with the numerous existing positive indications regarding neurodegenerative correlations that the family of crystallin proteins could play an important role in the development of innovative neuroprotective therapies for glaucoma. Several compounds, such as *L. barbarum* polysaccharide and 17β-estradiol, have already been found to exert considerable neuroprotective effects via the stimulation of retinal crystallin proteins in experimental animal models of glaucoma [33,52]. However, the up-regulation of crystallins in the retina appears to occur naturally as a reaction to neurodegeneration or IOP elevation, but this process appears to exhibit a temporal delay [15,16]. The overriding question is whether early treatment or generally higher levels of crystallin proteins can be a realistic treatment modality—combined with the classical IOP lowering procedure—in patients, and what underlying translational research is required. First and foremost, continuing studies are needed to reveal the mechanisms underlying the neuroprotective effects of crystallins in the retina, and especially which particular retinal cells primarily react to increased crystallin levels. Furthermore, advanced studies of the role of crystallin proteins in glaucoma would be needed in primates whose eye physiology and anatomy are more similar to those of humans. An up-regulation of crystallins has already been shown in human retinal tissue obtained postmortem from glaucoma patients [53]. However, despite this new knowledge about the indisputable potential of crystallins, there is still a considerable journey to cover from the laboratory bench to the patient bedsides.

## 4. Materials and Methods

### 4.1. Ethical Statement and Animal Experiments

All of the experiments were conducted in accordance with the Association of Research in Vision and Ophthalmology (ARVO)—Statement for the Use of Animals in Ophthalmic and Vision Research. Animal trials were approved by the committee for animal research (Health Investigation Office Rhineland-Palatinate, permission number: 14-1-085; approval date: 13 October 2014). In total, eleven Sprague Dawley rats, all female and of the same age, were used for this study. All animals were housed at the translational animal research center (TARC) of the University Medical Center of the Johannes–Gutenberg University Mainz with a twelve-hour day/night cycle. Food and water were provided ad libitum. Health and behavior of the animals were checked daily by schooled staff members of the TARC, and additionally by FELASA–B educated group members, as well as group’s veterinary surgeons on a weekly basis. During surgical interventions, minimizing the of animals’ discomfort and pain was of the highest priority. For anesthesia, a mixture of medetomidine (Dorbene vet., Pfizer, New York, NY, USA) and Ketamine (Inresa Arzneimittel, Freiburg, Germany) was injected intramuscularly into the hamstrings and oxybuprocain (Novesine, OmniVision, Puchheim, Germany) was applied topically on the eye. To reduce postoperative pain, novaminsulfon (Novalgin, Ratiopharm, Ulm, Germany) was injected subcutaneously after the surgery. Further investigations, which required a sedated status of the animals without an invasive intervention were conducted using medetomidine exclusively.

### 4.2. Episcleral Vein Cauterization

Experimental glaucoma was induced by the occlusion of three episcleral veins, which form the trunks of the vortex veins, as shown by Shareef et al. [54]. The episcleral veins were made accessible through a careful incision of the conjunctiva. Cauterization and subsequent transection of those vessels results in a considerable elevation of IOP approximately two weeks after surgery. The episcleral veins were made accessible through a careful incision of the conjunctiva. All of the animals received this surgical intervention on the left eye, while the contralateral eye served as a control throughout the study. To ensure the effect of the vein cauterization, the IOP was measured immediately before the surgery and once a week thereafter throughout the study. IOP measurement was performed using a rebound-tonolab (iCare, Vantaa, Finland), designed for rodents. Twenty individual measurements were taken per eye and subsequently averaged. Animals were only slightly fixated through handholding and fully conscious. Device calculated average values were ignored. The animals were sacrificed after seven weeks of elevated IOP.

### 4.3. Intravitreal Injection of α-Crystallin B

Recombinant produced and purified α-crystallin B (ABIN666647, antibodies-online, Aachen, Germany) was injected into the vitreous body of six cauterized eyes two weeks after the episcleral vein occlusion, immediately after the first rise of the IOP. As a control, the remaining five animals received an injection of phosphate-buffered saline (Sigma Aldrich, St. Louis, MO, USA) into the vitreous of their cauterized eyes. The injection volume for all animals was 5 µL. The protein concentration of the α-crystallin B stock was 1 µg/µL. To avoid a reflux of the injected fluid, the 30 G needle was kept intravitreal for a period of 15 s. Care was taken not damage the lens or other surrounding tissues. The amount of injected crystallin was adapted from previous studies focusing on β-crystallin proteins [15,35]. All of the injections were performed using a Hamilton Syringe (Sigma Aldrich). Contralateral eyes received no injections at all.

### 4.4. Quantification of Retinal Ganglion Cell Density

RGC loss was determined by immunohistochemical staining against the brain-specific homeobox/POU domain protein 3A (BRN3A) in retinal flat-mounts, as shown by Nadal-Nicolas et al. [55]. After sacrifice of the animals, one quarter of each retinal tissue was carefully separated with a micro-scissor and subsequently fixed in 4% formalin solution for 30 min (Carl Roth, Karsruhe, Germany). Subsequently, the retinal tissue parts were stored in 30% sucrose solution overnight and stained as previously described [15]. All of the retinal quarters were arranged at the site of the optic nerve head and 15 pictures were taken with a magnification of 200. Great care was taken to ensure an identical proportion of centrally and peripherally located retinal images for all individual retinal pieces. RGC quantification was performed using an ImageJ (ImageJ Fiji v_1) macro, which converted all of the pictures into a 16-bit grey scale format, following a background subtraction and auto-threshold application. The nucleus-counter particle analysis provided the final number of RGCs per picture. In addition, all pictures were manually checked for precision and accurateness of the macro.

### 4.5. Optical Coherence Tomography

Measurements of the retinal nerve fiber layer thickness (RNFLT) were performed on both eyes of all the animals at the beginning of the study before the vein cauterization, and immediately before the sacrifice. The in vivo imaging of the RNFL was accessed using a SD–OCT device (Heidelberg Engineering, Heidelberg, Germany). Pupils of the anesthetized animals were dilated by topical administration of tropicamide (Mydriaticum, Pharma Stulln, Stulln, Germany). To achieve a higher quality of the fundus picture and B-scan, a contact lens (PMMA 2.70/5.20, Cantor + Nissel, Brackley, UK) was placed on the eye during the optical coherence tomography measurements. To adjust the OCT device to rodents, the corneal radius was set to a fixed value of 7.7 mm and the reference arm and focus were adjusted manually for each animal to ensure the highest possible quality. A 12° dm circular B-scan around the optic nerve head with 100 frames per picture was taken. Subsequent segmentation of the RNFL and other retinal layers had to be performed manually with the aid of the Heidelberg Eye Explorer software v. 1.9.10.1. (Heidelberg Engineering), since the algorithm provides automated segmentation only for recordings in humans.

### 4.6. Ganzfeld Photopic Electroretinogram

All of the animals were anesthetized systemically and topically before the acquisition. After sedation, pupils were fully dilated with topical administration to a diameter of approximate 4 mm using Mydriaticum (Pharma Stulln). Thence, the animals were positioned properly on a custom-designed platform, and were connected with four electrodes (Roland Consult, Brandenburg, Germany): two goldring-electrodes (diameter: 4 mm) to both corneas as actives, one needle-electrode with dual cables for both eyes at the top of the head as reference, and one needle-electrode in the tail serving as ground, respectively. Following the placement of the electrodes, the eyes were lubricated with 2% Methocel (OmniVision, Puchheim, Germany) during the entire procedure. The Ganzfeld photopic ERG was recorded using a RETI system (Roland Consult): the background luminance was set to 40 cd·s·m^−2^ of green light; intensities of the white stimuli flash set to −0.15, 0.23, 0.61, 0.99, 1.37 log_10_ cd·s·m^−2^, respectively. The duration of the recording after stimulus was set to 512 ms (thereby sampling frequency 1 kHz). Artefacts were automatically filtered by the RETI system. Each record consisted of an average of over 25 responses obtained at the flash frequency of 0.33 Hz. Sufficiency of the anesthesia depth was monitored by the ERG signal baseline. After acquisition, raw data were exported from the system and plotted in GraphPad Prism v. 6 (GraphPad Software, San Diego, CA, USA). The amplitudes of the B-wave and the photopic negative response (PhNR) were further used for statistical analysis.

### 4.7. Mass Spectrometry Sample Preparation

Retinal tissue of α-crystallin B and PBS injected eyes was carefully separated from the parts used for IHC staining and transferred into individual 2 mL sample tubes in liquid nitrogen. The tissues were firstly disintegrated with a precooled mortar and subsequently lysed using 0.5% *N*-Dodecyl β-d-maltoside (Sigma Aldrich) in PBS for one hour at 4 °C. Further breakdown of the retinal cells was facilitated using an ultrasonic bath for 30 min on ice and multiple impulses of an ultrasonic wand. The protein concentration for each sample was determined with a BCA Pierce Protein Assay kit (Thermo Fisher, Waltham, MA, USA), and 10 µg of the total protein mixture was transferred into 1× LDS sample buffer (NuPAGE, Thermo Fisher) containing 0.1 M DTT. Each sample was subsequently boiled at 70 °C for ten minutes and separated on a 4–12% NuPAGE Novex Bis-Tris precast gel (Life Technologies, Waltham, MA, USA) for ten minutes at 180 V in 1× MOPS buffer. The individual gel lanes were further cut into one piece per lane and were destained with 50% ethanol and 25 mM ammonium bicarbonate (ABC). After dehydration with 100% acetonitrile (ACN), the gel pieces were digested using trypsin at 37 °C overnight. The tryptic peptides were extracted twice with 3% TFA and 30% CAN, concentrated with an Eppendorf concentrator and passed through a C_18_ Stage Tip [56].

### 4.8. MS Measurement and Data Analysis

The samples were injected through the autosampler unit into an Ultra High Performance Liquid Chromatography (uHPLC) system (EASY-nLC 1000, Thermo Fisher). The peptides were subsequently loaded on a 25-cm capillary (C18-AQ 1.9 µm resin, Dr. Maisch GmbH, Ammerbuch, Germany) for the reverse-phase chromatography. The HPLC system was directly mounted to a Q Exactive Plus mass spectrometer (Thermo Fisher) with a 90 min optimized gradient from 2 to 40% ACN with 0.1% formic acid at a flow rate of 200 nL/min. Chromatography stabilization, spray voltage range, and MS mode were the same, as previously described [57]. The MS full scans were obtained in the orbitrap with a resolution of 70,000, while the MS/MS scan resolution was set to 17,500. Obtained raw files were processed with MaxQuant (version 1.5.3.30) and search against the Swiss-Prot annotated protein database of Rattus norvegicus (10116). Carbamidomethyl (Cys) was set as fixed modification, while acetyl (N-term protein) and oxidation (Met) were considered as variable modifications. Only proteins with at least two ratio counts based on unmodified unique- or razor-peptides were quantified with an applied false-discovery rate (FDR) of 1%. Identified proteins with a ratio count below two were not reported into the final files. The obtained label-free quantification (LFQ) intensities were further used for the fold-change ratio calculation. The mass spectrometry proteomics data have been deposited to the ProteomeXchange Consortium via the PRIDE [58] partner repository with the dataset identifier PXD007751. Additionally to the raw files in the repository, all proteins, including their regulation fold-change and LFQ intensity, can be found in the supplementary files.

### 4.9. Antibody Microarray

Lamin A/C (0.1 mg/mL), β-crystallin B2 (0.25 mg/mL), and γ-crystallin C (1 mg/mL) antibodies were spotted with nine technical replicates per subarray on a glass-nitrocellulose 16 multi-pad slide (Oncyte, Grace Bio-Labs, Bend, OR, USA) using a non-contact array spotter (ciFLEXARRAYER 3, Scienion, Berlin, Germany). As a loading control, GAPDH (1 mg/mL) antibodies were spotted on the same subarrays in the same manner. The retinal protein mix was labeled with Dylight 649 NHS Ester (Cy5) (Thermo Fisher) following the manufactures protocol. After one hour of incubation with the dye, the reaction was stopped by adding 100 µL of HCl-Tris for another hour. In the meantime, the array slide was blocked with Grace Bio-Labs Super G blocking buffer (Sigma Aldrich) for one hour and subsequently washed with 0.5% Tween in PBS (three times, 10 min). Afterwards the Dylight-labeled protein mixes (10 µg in total) from the particular experimental groups were loaded onto the individual subarrays and incubated for 2.5 h. To remove unbound proteins, the slide was washed again three times with 0.5% Tween in PBS and subsequently dried in a SpeedVac-Concentrator for two minutes (Eppendorf, Hamburg, Germany). Finally, the emitted fluorescence signals were scanned with a high-resolution confocal array scanner (Affymetrix 428, Santa Clara, CA, USA) at a gain of 10 dB and a line average of 10. Digitized signals were analyzed with Imagene 5.5 (BioDiscovery Inc., El Segundo, CA, USA).

### 4.10. Bioinformatics Analysis and Proteomic Networks

Considerably altered proteins (exp. Fold-change > 1.5), determined by their relative regulation between the crystallin- and the PBS-injected group, were further processed to the Ingenuity Pathway Analysis IPA^®^ software (version v.01-04, Qiagen, Venlo, The Netherlands). Protein relations and networks were calculated accordingly using Fisher’s exact test with a significance level of 0.05. Only altered proteins were provided to the IPA^®^ software in order to generate the interaction network. However IPA^®^ may add a minor amount of additional proteins to generate a broader spectrum of protein/protein relations.

### 4.11. Experimental Design and Statistics

All data obtained from the experimental groups of α-crystallin B injected eyes, PBS injected eyes and untreated contralateral eyes was checked for Gaussian distribution. Parametric *t*-tests and one-way ANOVA testing was performed accordingly using Prism GraphPad v.6. A *p*-value ≤ 0.05 was considered as statistically significant. If not indicated differently, all data is provided with mean ± SD.

## Supplementary Materials

Supplementary materials can be found at www.mdpi.com/1422-0067/18/11/2418/s1.

## Abbreviations

ACN	Acetonitrile
AMD	Age-related macular degeneration
ARVO	Association of Research in Vision and Ophthalmology
BRN3A	Brain-specific homeobox/POU domain protein 3A
CNTF	Ciliary neurotrophic factor
Cryab	α-crystallin B
ERG	Electroretinogram
FDR	False discovery rate
GAPDH	Glyceraldehyde 3-phosphate dehydrogenase
IL-6	Interleukin 6
IOP	Intraocular pressure
IV	intravitreal
LFQ	Label-free quantification
MS	Mass-spectrometry
PBS	phosphate-buffered saline
PhNR	Photopic negative response
RGC	Retinal ganglion cell
RNFL	Retinal nerve fiber layer
RNFLT	Retinal nerve fiber layer thickness
sHSP	Small heat shock protein
TARC	Translational animal research center
uHPLC	Ultra-high pressure liquid chromatography
UVA	Ultraviolet A radiation
VEGF	Vascular endothelial growth factor
