# Supplementary material for: The Small Heat Shock Protein α-Crystallin B Shows Neuroprotective Properties in a Glaucoma Animal Model
Source: Int J Mol Sci. 2017 Nov 14;18(11):2418. doi: 10.3390/ijms18112418 (PMC5713386; doi:10.3390/ijms18112418)
Supplement: Supplementary file 1 [file ijms-18-02418-s001.zip › 4.0.Supplementary table regulated proteins.pdf]

Protein name	Gene name	Fold-change ratio	Regulation	Mean LFQ intensity Alpha Crystallin B Group	Mean LFQ intensity PBS Group
40S ribosomal protein S15	*Rps15*	1.93	Down	54261000	104496000
60S ribosomal protein L32	*Rpl32*	1.55	Down	511965000	792492500
Acetyl-CoA carboxylase 1;Biotin carboxylase	*Acaca*	1.72	Up	254480000	147957500
Activated RNA polymerase II transcriptional coactivator p15	*Sub1*	2.24	Up	1161980000	518802500
Activity-dependent neuroprotector homeobox protein	*Adnp*	1.82	Down	62292500	113136000
ADP-sugar pyrophosphatase	*Nudt5*	2.13	Up	130560000	61229000
Alpha-1B-glycoprotein	*A1bg*	1.68	Up	142690000	84804000
Alpha-internexin	*Ina*	2.32	Down	636297500	1474150000
Anion exchange protein 2	*Slc4a2*	1.57	Up	75682500	48292000
ATP synthase protein 8	*Mt-atp8*	1.72	Up	210400000	122442500
ATP-binding cassette sub-family A member 2	*Abca2*	1.78	Down	888925000	1581000000
Beta-arrestin-1	*Arrb1*	2.07	Up	595772500	288182500
Beta-crystallin A3;Beta-crystallin A3	*Cryba1*	1.58	Up	271492500	171631500
Beta-crystallin A4	*Cryba4*	3.39	Up	85191750	25144500
Beta-crystallin B2	*Crybb2*	1.89	Up	1062927500	561807500
Beta-crystallin S	*Crygs*	1.68	Up	107755000	64106750
Calcium-binding mitochondrial carrier protein SCaMC-2	*Slc25a25*	1.56	Up	108535333	69402000
Casein kinase I isoform delta	*Csnk1d*	2.17	Down	10006000	21690000
Cellular nucleic acid-binding protein	*Cnbp*	1.54	Down	203021250	312617500
Clathrin light chain A	*Clta*	1.77	Up	846083333	478195000
Clusterin;Clusterin beta chain;Clusterin alpha chain	*Clu*	1.91	Up	189795000	99412500
Creatine kinase M-type	*Ckm*	1.70	Up	641130000	377383333
Diphosphoinositol polyphosphate phosphohydrolase 2	*Nudt4*	1.51	Up	59385000	39292000
DNA topoisomerase 1	*Top1*	1.52	Up	57442000	37900000
DNA topoisomerase 2-alpha	*Top2a*	1.81	Up	152155000	83870333.3
Dual specificity tyrosine-phosphorylation-regulated kinase 1A	*Dyrk1a*	1.60	Down	74864000	119740000
Electron transfer flavoprotein-ubiquinone oxidoreductase, mitochondrial	*Etfdh*	1.66	Up	133353333	80505000
Eukaryotic translation initiation factor 1A	*Eif1a*	1.67	Up	102425500	61270000
Fibrinogen alpha chain;Fibrinopeptide A;Fibrinogen alpha chain	*Fga*	1.86	Down	65288500	121578750

Filamin-C	*Flnc*	1.55	Up	222650000	143610000
Gamma-crystallin B	*Crygb*	1.70	Down	168032500	285753333
Gamma-crystallin C	*Crygc*	1.99	Up	41397650	20802250
Glycine cleavage system H protein, mitochondrial	*Gcsh*	1.76	Up	142490000	80924000
Golgi resident protein GCP60	*Acbd3*	1.97	Up	117733500	59821500
GPI inositol-deacylase	*Pgap1*	1.74	Down	81943500	142834750
Guanine nucleotide-binding protein G(I)/G(S)/G(O) subunit gamma-11	*Gng11*	2.00	Down	317790000	634080000
Hemoglobin subunit beta-2	*Hbb-b2*	2.31	Down	1296967500	2997475000
Histone H2A type 2-A	*Hist2h2aa3*	1.99	Down	4218075000	8381700000
Histone H2B type 1;Histone H2B type 1-A	*Hist1h2ba*	1.78	Down	4303400000	7642825000
Histone H3.3;Histone H3.1	*H3f3b*	1.87	Down	925393333	1734025000
Histone H4;Osteogenic growth peptide	*Hist1h4b*	2.07	Down	2266950000	4700975000
Lamin-B1	*Lmnb1*	3.42	Down	1433960000	4896975000
Large neutral amino acids transporter small subunit 1	*Slc7a5*	5.14	Up	132143000	25704000
Liprin-alpha-3	*Ppfia3*	1.50	Up	132940000	88788000
Methyl-CpG-binding protein 2	*Mecp2*	1.84	Down	217625000	399940000
Mitochondrial pyruvate carrier 1	*Mpc1*	2.28	Up	796500000	348980000
Myristoylated alanine-rich C-kinase substrate	*Marcks*	2.47	Up	1760595000	713300000
NAD-dependent protein deacylase sirtuin-5, mitochondrial	*Sirt5*	1.88	Up	172790000	91968666.7
Neurofilament light polypeptide	*Nefl*	1.98	Down	545370000	1078150000
Neuronal calcium sensor 1	*Ncs1*	1.79	Down	42340000	75598000
Non-histone chromosomal protein HMG-17	*Hmgn2*	1.51	Down	316087500	476235000
Nucleoporin NUP53	*Nup35*	1.84	Down	61615000	113388667
Nucleoprotein TPR	*Tpr*	1.54	Down	393040000	606462500
NudC domain-containing protein 2	*Nudcd2*	2.07	Up	85053333.3	41075000
Peptidyl-prolyl cis-trans isomerase F, mitochondrial	*Ppif*	1.68	Down	49242000	82583000
Peroxisomal biogenesis factor 19	*Pex19*	1.74	Up	42736000	24579000
Peroxisomal membrane protein PEX14	*Pex14*	1.64	Up	101900000	62250000
Phospholipid hydroperoxide glutathione peroxidase	*Gpx4*	1.99	Up	1290360000	647730000
Plectin	*Plec*	1.71	Down	554365000	949052500
Podocalyxin	*Podxl*	2.08	Up	119540500	57478000
Polyamine-modulated factor 1-binding protein 1	*Pmfbp1*	2.49	Up	755560000	302990000
Prelamin-A/C;Lamin-A/C	*Lmna*	3.58	Down	341547500	1222425000

Probable 2-oxoglutarate dehydrogenase E1 component DHKTD1, mitochondrial	*Dhtkd1*	1.51	Down	35048500	52998000
Prosaposin receptor GPR37	*Gpr37*	2.10	Up	671413333	319330000
Protein BUD31 homolog	*Bud31*	2.88	Down	28350666.7	81558275
Protein Hikeshi	*HIKESHI*	2.18	Down	38476000	84060000
Protein phosphatase inhibitor 2	*Ppp1r2*	1.56	Down	170503333	265972500
Protein THEM6	*Them6*	1.56	Up	98520000	63031000
Ras-related protein Rab-35	*Rab35*	1.55	Down	372712500	576117500
Receptor-type tyrosine-protein phosphatase alpha	*Ptpra*	2.17	Down	26060000	56626000
Retinal dehydrogenase 1	*Aldh1a1*	11.69	Down	91349750	1067577500
RNA-binding motif protein, X chromosome retrogene-like	*Rbmxrtl;Rbmxl1*	3.02	Up	232250000	76812000
RNA-binding protein 8A	*Rbm8a*	1.52	Down	254630000	386995000
Serine protease inhibitor A3K	*Serpina3k*	1.51	Down	132024667	199932500
Serine/threonine-protein kinase 24	*Stk24*	1.61	Up	104294333	64753000
Serpin H1	*Serpinh1*	1.61	Up	193510000	120133333
SH3 domain-containing kinase-binding protein 1	*Sh3kbp1*	1.86	Up	47868000	25696500
THO complex subunit 6 homolog	*Thoc6*	2.04	Up	80596000	39597500
Threonine--tRNA ligase, mitochondrial	*Tars2*	1.59	Up	84227500	52861000
Transcriptional repressor CTCF	*Ctcf*	1.90	Down	34962666.7	66479250
Trophoblast glycoprotein	*Tpbg*	1.72	Down	49395000	84988000
Tubulin polymerization-promoting protein family member 3	*Tppp3*	1.57	Down	350595000	549615000
Tyrosine 3-monooxygenase	*Th*	1.87	Down	54232000	101462000
Urea transporter 1	*Slc14a1*	1.75	Up	367257500	209727500
Very-long-chain 3-oxoacyl-CoA reductase	*Hsd17b12*	1.51	Up	283850000	187745000
WD repeat-containing protein 6	*Wdr6*	1.92	Down	10836000	20762000
Zinc finger protein 652	*Znf652*	1.63	Down	7768200	12693000
