# Supplementary material for: The Small Heat Shock Protein α-Crystallin B Shows Neuroprotective Properties in a Glaucoma Animal Model
Source: Int J Mol Sci. 2017 Nov 14;18(11):2418. doi: 10.3390/ijms18112418 (PMC5713386; doi:10.3390/ijms18112418)
Supplement: Supplementary file 1 [file ijms-18-02418-s001.zip › 4.0.Supplementary table all proteins.pdf]

Protein name	Gene name	Fold-change ratio	Regulation	Mean intensity Alpha Crystallin B Group	Mean intensity PBS Group
[Protein ADP-ribosylarginine] hydrolase	*Adprh*	1.05	Down	257455000	269192500
1,2-dihydroxy-3-keto-5-methylthiopentene dioxygenase	*Adi1*	1.07	Down	178780000	191163333.3
1,5-anhydro-D-fructose reductase	*Akr1e2*	1.09	Up	137495000	125682500
10 kDa heat shock protein, mitochondrial	*Hspe1*	1.08	Up	3640325000	3373075000
14-3-3 protein beta/alpha;14-3-3 protein beta/alpha, N-terminally processed	*Ywhab*	1.15	Down	4566050000	5269750000
14-3-3 protein epsilon	*Ywhae*	1.06	Up	28391750000	26677000000
14-3-3 protein eta	*Ywhah*	1.29	Up	1480585000	1143457500
14-3-3 protein gamma;14-3-3 protein gamma, N-terminally processed	*Ywhag*	1.28	Up	9173550000	7143750000
14-3-3 protein theta	*Ywhaq*	1.17	Down	7575725000	8845300000
14-3-3 protein zeta/delta	*Ywhaz*	1.02	Up	31803750000	31303000000
15 kDa selenoprotein	*Sep--15*	1.16	Up	106431333.3	91792333.33
1-phosphatidylinositol 4,5-bisphosphate phosphodiesterase beta-1	*Plcb1*	1.27	Up	98442000	77787000
1-phosphatidylinositol 4,5-bisphosphate phosphodiesterase delta-1	*Plcd1*	1.03	Up	499362500	485430000
2,3-cyclic-nucleotide 3-phosphodiesterase	*Cnp*	1.16	Up	197505000	169940000
2,5-phosphodiesterase 12	*Pde12*	1.19	Up	205310000	171906666.7
26S protease regulatory subunit 4	*Psmc1*	1.07	Down	1305142500	1390025000
26S protease regulatory subunit 6A	*Psmc3*	1.03	Down	948675000	974095000
26S protease regulatory subunit 6B	*Psmc4*	1.03	Up	1044927500	1016400000
26S protease regulatory subunit 7	*Psmc2*	1.17	Up	2649625000	2260000000
26S protease regulatory subunit 8	*Psmc5*	1.15	Down	1339350000	1538450000
26S proteasome non-ATPase regulatory subunit 1	*Psmd1*	1.02	Up	1608900000	1573750000
26S proteasome non-ATPase regulatory subunit 11	*Psmd11*	1.04	Up	1764875000	1689700000
26S proteasome non-ATPase regulatory subunit 13	*Psmd13*	1.04	Up	1525950000	1473150000
26S proteasome non-ATPase regulatory subunit 2	*Psmd2*	1.05	Down	2750025000	2898425000
26S proteasome non-ATPase regulatory subunit 9	*Psmd9*	1.01	Up	309247500	306500000
2-oxoglutarate dehydrogenase, mitochondrial	*Ogdh*	1.06	Up	4804525000	4541475000
2-oxoisovalerate dehydrogenase subunit alpha, mitochondrial	*Bckdha*	1.15	Down	52596500	60674500
3(2),5-bisphosphate nucleotidase 1	*Bpnt1*	1.04	Down	2327075000	2419275000
3-hydroxyacyl-CoA dehydrogenase type-2	*Hsd17b10*	1.11	Down	633920000	700607500
3-hydroxyisobutyrate dehydrogenase, mitochondrial	*Hibadh*	1.10	Up	3260550000	2952425000
3-hydroxyisobutyryl-CoA hydrolase, mitochondrial	*Hibch*	1.08	Up	385260000	355667500
3-ketoacyl-CoA thiolase A, peroxisomal;3-ketoacyl-CoA thiolase B, peroxisomal	*Acaa1a;Acaa1b*	1.10	Up	689237500	625102500
3-ketoacyl-CoA thiolase, mitochondrial	*Acaa2*	1.08	Up	221207500	205072500
3-mercaptopyruvate sulfurtransferase	*Mpst*	1.14	Up	667047500	582827500
3-phosphoinositide-dependent protein kinase 1	*Pdpk1*	1.12	Down	39553000	44429000
40S ribosomal protein S10	*Rps10*	1.08	Up	916310000	849555000
40S ribosomal protein S11	*Rps11*	1.05	Up	1330950000	1268050000
40S ribosomal protein S12	*Rps12*	1.06	Up	1467050000	1378925000
40S ribosomal protein S13	*Rps13*	1.07	Up	2175600000	2037975000
40S ribosomal protein S14	*Rps14*	1.27	Up	537087500	423647500
40S ribosomal protein S15	*Rps15*	1.93	Down	54261000	104496000
40S ribosomal protein S15a	*Rps15a*	1.06	Up	1880650000	1768700000
40S ribosomal protein S16	*Rps16*	1.22	Up	1647425000	1352550000
40S ribosomal protein S17	*Rps17*	1.06	Down	993427500	1049500000
40S ribosomal protein S18	*Rps18*	1.15	Up	1693150000	1472975000

40S ribosomal protein S19	*Rps19*	1.18	Up	2143725000	1813525000
40S ribosomal protein S20	*Rps20*	1.48	Up	1026377500	692472500
40S ribosomal protein S21	*Rps21*	1.26	Up	113720000	90330000
40S ribosomal protein S23	*Rps23*	1.14	Down	563745000	641480000
40S ribosomal protein S24	*Rps24*	1.21	Up	600610000	497205000
40S ribosomal protein S25	*Rps25*	1.12	Up	1911625000	1701475000
40S ribosomal protein S26	*Rps26*	1.16	Up	722152500	622260000
40S ribosomal protein S27	*Rps27*	1.45	Up	338790000	232900000
40S ribosomal protein S29	*Rps29*	1.14	Down	290220000	331617500
40S ribosomal protein S3	*Rps3*	1.09	Up	3608025000	3305800000
40S ribosomal protein S3a;40S ribosomal protein S3b	*Rps3a*	1.04	Down	2284700000	2369375000
40S ribosomal protein S4, X isoform	*Rps4x*	1.11	Down	2918725000	3229850000
40S ribosomal protein S5;40S ribosomal protein S5, N-terminally processed	*Rps5*	1.02	Up	1149687500	1121727500
40S ribosomal protein S6	*Rps6*	1.22	Up	634252500	521027500
40S ribosomal protein S7	*Rps7*	1.07	Up	1881800000	1754150000
40S ribosomal protein S8	*Rps8*	1.02	Down	2324925000	2361200000
40S ribosomal protein S9	*Rps9*	1.14	Up	668830000	584300000
40S ribosomal protein SA	*Rpsa*	1.03	Up	4319850000	4184700000
4-aminobutyrate aminotransferase, mitochondrial;4-aminobutyrate aminotransferase, brain isoform;4-aminobutyrate aminotransferase, liver isoform	*Abat*	1.03	Up	10636300000	10277150000
4F2 cell-surface antigen heavy chain	*Slc3a2*	1.09	Up	5547950000	5103125000
4-trimethylaminobutyraldehyde dehydrogenase	*Aldh9a1*	1.05	Down	2645750000	2770100000
5-nucleotidase	*Nt5e*	1.22	Up	637675000	524675000
5-nucleotidase domain-containing protein 2	*Nt5dc2*	1.28	Up	98444000	76857000
5-oxoprolinase	*Oplah*	1.14	Up	125896666.7	110167500
6.8 kDa mitochondrial proteolipid	*Mp68*	1.14	Up	418130000	367806666.7
60 kDa heat shock protein, mitochondrial	*Hspd1*	1.04	Up	13963250000	13412250000
60S acidic ribosomal protein P0	*Rplp0*	1.09	Up	3038225000	2777275000
60S acidic ribosomal protein P1	*Rplp1*	1.23	Down	593967500	729493333.3
60S acidic ribosomal protein P2	*Rplp2*	1.25	Up	173975000	139360000
60S ribosomal protein L10	*Rpl10*	1.25	Down	912750000	1144975000
60S ribosomal protein L10a	*Rpl10a*	1.10	Up	2076125000	1888875000
60S ribosomal protein L11	*Rpl11*	1.14	Down	1360992500	1558150000
60S ribosomal protein L12	*Rpl12*	1.10	Down	1771925000	1945600000
60S ribosomal protein L13	*Rpl13*	1.16	Down	1027702500	1188047500
60S ribosomal protein L13a	*Rpl13a*	1.10	Down	152182500	168000000
60S ribosomal protein L14	*Rpl14*	1.11	Up	935482500	843952500
60S ribosomal protein L15	*Rpl15*	1.02	Up	1389175000	1362600000
60S ribosomal protein L17	*Rpl17*	1.07	Down	525085000	561830000
60S ribosomal protein L18	*Rpl18*	1.35	Up	114355000	84521000
60S ribosomal protein L18a	*Rpl18a*	1.08	Down	1198927500	1293650000
60S ribosomal protein L19	*Rpl19*	1.01	Down	543222500	551110000
60S ribosomal protein L21	*Rpl21*	1.13	Up	600377500	532590000
60S ribosomal protein L22	*Rpl22*	1.15	Down	463772500	535625000
60S ribosomal protein L23	*Rpl23*	1.13	Up	1345450000	1186150000
60S ribosomal protein L23a	*Rpl23a*	1.10	Down	906235000	995380000
60S ribosomal protein L24	*Rpl24*	1.11	Up	835130000	750180000
60S ribosomal protein L26	*Rpl26*	1.02	Down	946687500	961825000
60S ribosomal protein L27	*Rpl27*	1.10	Up	1519625000	1380675000
60S ribosomal protein L27a	*Rpl27a*	1.07	Down	656546666.7	701147500
60S ribosomal protein L28	*Rpl28*	1.02	Down	413895000	420792500
60S ribosomal protein L29	*Rpl29*	1.03	Down	287025000	296287500
60S ribosomal protein L3	*Rpl3*	1.02	Up	1451900000	1424825000
60S ribosomal protein L30	*Rpl30*	1.12	Up	1111527500	991357500
60S ribosomal protein L31	*Rpl31*	1.04	Up	760957500	729600000
60S ribosomal protein L32	*Rpl32*	1.55	Down	511965000	792492500

60S ribosomal protein L34	*Rpl34*	1.01	Up	266687500	264167500
60S ribosomal protein L35	*Rpl35*	1.41	Up	636755000	452895000
60S ribosomal protein L35a	*Rpl35a*	1.10	Up	377240000	341496666.7
60S ribosomal protein L36	*Rpl36*	1.05	Up	341623333.3	324070000
60S ribosomal protein L37a;Putative 60S ribosomal protein L37a	*Rpl37a;Rpl37a-ps1*	1.10	Up	184147500	166670000
60S ribosomal protein L38	*Rpl38*	1.04	Down	499545000	517732500
60S ribosomal protein L4	*Rpl4*	1.12	Up	834195000	746915000
60S ribosomal protein L5	*Rpl5*	1.08	Up	2899450000	2685300000
60S ribosomal protein L6	*Rpl6*	1.19	Up	286527500	241617500
60S ribosomal protein L7	*Rpl7*	1.26	Up	173473333.3	137883333.3
60S ribosomal protein L7a	*Rpl7a*	1.12	Up	2025175000	1815675000
60S ribosomal protein L8	*Rpl8*	1.37	Down	1050802500	1440800000
60S ribosomal protein L9	*Rpl9*	1.08	Up	1534050000	1419225000
6-phosphogluconate dehydrogenase, decarboxylating	*Pgd*	1.02	Up	2681025000	2621600000
6-phosphogluconolactonase	*Pgls*	1.07	Up	883607500	822217500
78 kDa glucose-regulated protein	*Hspa5*	1.06	Up	9451150000	8902225000
7-methylguanosine phosphate-specific 5-nucleotidase	*Nt5c3b*	1.43	Down	32874000	47013000
Abhydrolase domain-containing protein 16A	*Abhd16a*	1.06	Up	300337500	283630000
Abl interactor 1	*Abi1*	1.01	Up	509955000	503755000
Acetylcholinesterase	*Ache*	1.05	Down	147713333.3	154377500
Acetyl-CoA acetyltransferase, cytosolic	*Acat2*	1.02	Up	848120000	827932500
Acetyl-CoA acetyltransferase, mitochondrial	*Acat1*	1.09	Down	2068875000	2254475000
Acetyl-CoA carboxylase 1;Biotin carboxylase	*Acaca*	1.72	Up	254480000	147957500
Acid ceramidase	*Asah1*	1.47	Up	368830000	251067500
Acidic leucine-rich nuclear phosphoprotein 32 family member A	*Anp32a*	1.02	Down	10632275000	10841725000
Acidic leucine-rich nuclear phosphoprotein 32 family member B	*Anp32b*	1.02	Down	2343125000	2381275000
Acidic leucine-rich nuclear phosphoprotein 32 family member E	*Anp32e*	1.07	Up	5487725000	5135225000
Aconitate hydratase, mitochondrial	*Aco2*	1.05	Up	20599750000	19698000000
Actin, alpha cardiac muscle 1;Actin, alpha skeletal muscle	*Actc1;Acta1*	1.37	Down	12702000000	17356250000
Actin, aortic smooth muscle;Actin, gamma-enteric smooth muscle	*Acta2;Actg2*	1.35	Down	472876666.7	637197500
Actin, cytoplasmic 2;Actin, cytoplasmic 2, N-terminally processed	*Actg1*	1.01	Up	1.02274E+11	1.01394E+11
Actin-binding LIM protein 2	*Ablim2*	1.08	Up	144640000	134360000
Actin-related protein 2	*Actr2*	1.04	Down	1398825000	1453100000
Actin-related protein 2/3 complex subunit 1A	*Arpc1a*	1.09	Up	1410200000	1298225000
Actin-related protein 2/3 complex subunit 2	*Arpc2*	1.06	Down	1426550000	1510550000
Actin-related protein 2/3 complex subunit 5	*Arpc5*	1.32	Up	250406666.7	189970000
Actin-related protein 2/3 complex subunit 5-like protein	*Arpc5l*	1.01	Up	187670000	184930000
Actin-related protein 3	*Actr3*	1.04	Up	2093600000	2017725000
Activated RNA polymerase II transcriptional coactivator p15	*Sub1*	2.24	Up	1161980000	518802500
Activity-dependent neuroprotector homeobox protein	*Adnp*	1.82	Down	62292500	113136000
Acylamino-acid-releasing enzyme	*Apeh*	1.32	Up	218070000	165086666.7
Acyl-CoA-binding protein;Triakontatetraneuropeptide;Octadecaneuropeptide	*Dbi*	1.10	Down	2180975000	2390150000

Acyl-coenzyme A thioesterase 2, mitochondrial;Acyl-coenzyme A thioesterase 1	*Acot2;Acot1*	1.12	Down	126033333.3	140630000
Acyl-coenzyme A thioesterase 8	*Acot8*	1.13	Up	63475500	56354000
Acyl-coenzyme A thioesterase THEM4	*Them4*	1.10	Up	159545000	145390000
Acyl-protein thioesterase 1	*Lypla1*	1.02	Down	401245000	410466666.7
Acyl-protein thioesterase 2	*Lypla2*	1.01	Up	1242650000	1236275000
Acylpyruvase FAHD1, mitochondrial	*Fahd1*	1.07	Down	183760000	196827500
Adapter molecule crk	*Crk*	1.08	Up	706272500	654675000
Adaptin ear-binding coat-associated protein 1	*Necap1*	1.01	Down	319975000	324070000
Adenine phosphoribosyltransferase	*Aprt*	1.01	Up	661190000	655415000
Adenosine kinase	*Adk*	1.08	Down	701867500	756380000
Adenosylhomocysteinase	*Ahcy*	1.15	Down	13610500000	15655000000
Adenosylhomocysteinase	*Ahcyl1*	1.19	Down	2421700000	2887000000
Adenylate kinase 4, mitochondrial	*Ak4*	1.05	Down	181402500	190592500
Adenylate kinase isoenzyme 1	*Ak1*	1.05	Down	4301300000	4536850000
Adenylyl cyclase-associated protein 1	*Cap1*	1.21	Down	2795375000	3380875000
Adenylyl cyclase-associated protein 2	*Cap2*	1.25	Down	143705000	180296666.7
Adipocyte plasma membrane-associated protein	*Apmap*	1.16	Down	415505000	479962500
ADP/ATP translocase 1	*Slc25a4*	1.05	Up	37247750000	35407250000
ADP/ATP translocase 2;ADP/ATP translocase 2, N-terminally processed	*Slc25a5*	1.22	Up	10214525000	8366075000
ADP-ribose pyrophosphatase, mitochondrial	*Nudt9*	1.01	Down	310900000	314560000
ADP-ribosylation factor 1;ADP-ribosylation factor 3	*Arf1;Arf3*	1.17	Up	4661300000	3999650000
ADP-ribosylation factor 4	*Arf4*	1.11	Up	405750000	364362500
ADP-ribosylation factor 5	*Arf5*	1.09	Up	4263550000	3910525000
ADP-ribosylation factor 6	*Arf6*	1.13	Up	465700000	413247500
ADP-ribosylation factor GTPase-activating protein 1	*Arfgap1*	1.01	Up	120410000	118670000
ADP-ribosylation factor GTPase-activating protein 2	*Arfgap2*	1.28	Up	94163000	73747000
ADP-ribosylation factor-like protein 1	*Arl1*	1.04	Up	255546666.7	244767500
ADP-ribosylation factor-like protein 3	*Arl3*	1.05	Down	1806000000	1892225000
ADP-ribosylation factor-like protein 8B	*Arl8b*	1.02	Down	863895000	881722500
ADP-sugar pyrophosphatase	*Nudt5*	2.13	Up	130560000	61229000
Afadin	*Mllt4*	1.09	Up	208563333.3	191273333.3
Aflatoxin B1 aldehyde reductase member 2	*Akr7a2*	1.10	Down	2254675000	2486050000
Agrin;Agrin N-terminal 110 kDa subunit;Agrin C-terminal 110 kDa subunit;Agrin C-terminal 90 kDa fragment;Agrin C-terminal 22 kDa fragment	*Agrn*	1.28	Up	82287000	64526000
AH receptor-interacting protein	*Aip*	1.00	Up	374242500	373342500
A-kinase anchor protein 12	*Akap12*	1.10	Up	242190000	221150000
A-kinase anchor protein SPHKAP	*Sphkap*	1.10	Up	130840000	119040000
Alanine--tRNA ligase, cytoplasmic	*Aars*	1.02	Up	3537650000	3451400000
Alanyl-tRNA editing protein Aarsd1	*Aarsd1*	1.02	Down	391677500	401222500
Alcohol dehydrogenase [NADP(+)]	*Akr1a1*	1.09	Down	4444050000	4822525000
Alcohol dehydrogenase class-3	*Adh5*	1.01	Down	3352925000	3380875000
Aldehyde dehydrogenase X, mitochondrial	*Aldh1b1*	1.20	Up	113945000	94674333.33
Aldehyde dehydrogenase, mitochondrial	*Aldh2*	1.09	Up	665385000	613030000
Aldose reductase	*Akr1b1*	1.02	Up	9723375000	9498500000
Aldose reductase-related protein 1	*Akr1b7*	1.14	Down	699993333.3	800652500
Alpha/beta hydrolase domain-containing protein 14B	*Abhd14b*	1.07	Up	1364250000	1276375000
Alpha-1-antiproteinase	*Serpina1*	1.04	Down	302680000	314587500
Alpha-1B-glycoprotein	*A1bg*	1.68	Up	142690000	84804000
Alpha-1-inhibitor 3	*A1i3*	1.16	Down	1021540000	1186125000

Alpha-1-macroglobulin;Alpha-1-macroglobulin 45 kDa subunit	*A1m*	1.05	Down	800012500	841485000
Alpha-2-HS-glycoprotein	*Ahsg*	1.19	Up	175655000	147322500
Alpha-2-macroglobulin	*A2m*	1.50	Down	535727500	803017500
Alpha-2-macroglobulin receptor-associated protein	*Lrpap1*	1.05	Up	154600000	146760000
Alpha-actinin-1	*Actn1*	1.12	Down	229507500	257615000
Alpha-actinin-4	*Actn4*	1.04	Down	7128725000	7439875000
Alpha-adducin	*Add1*	1.13	Down	4178075000	4729775000
Alpha-aminoadipic semialdehyde dehydrogenase	*Aldh7a1*	1.00	Up	1916500000	1910850000
Alpha-centractin	*Actr1a*	1.10	Up	3383100000	3080450000
Alpha-crystallin A chain;Alpha-crystallin A(1-168);Alpha-crystallin A(1-165);Alpha-crystallin A(1-163);Alpha-crystallin A(1-162);Alpha-crystallin A(1-157);Alpha-crystallin A(1-156);Alpha-crystallin A(1-151)	*Cryaa*	1.22	Up	840432500	690380000
Alpha-crystallin B chain	*Cryab*	1.22	Up	541445000	445427500
Alpha-crystallin B chain	*Cryab*	1.12	Up	630605000	564887500
Alpha-enolase	*Eno1*	1.04	Up	1.69143E+11	1.623E+11
Alpha-internexin	*Ina*	2.32	Down	636297500	1474150000
Alpha-mannosidase 2C1	*Man2c1*	1.11	Up	181055000	162952500
Alpha-methylacyl-CoA racemase	*Amacr*	1.10	Up	50960333.33	46536250
Alpha-N-acetylgalactosaminidase	*Naga*	1.01	Down	145817500	147486666.7
Alpha-soluble NSF attachment protein	*Napa*	1.07	Up	1635600000	1533625000
Amine oxidase [flavin-containing] A	*Maoa*	1.07	Up	1697075000	1580975000
Amine oxidase [flavin-containing] B	*Maob*	1.34	Down	298875000	400160000
Aminoacyl tRNA synthase complex-interacting multifunctional protein 2	*Aimp2*	1.12	Down	192152000	214738500
Aminoacylase-1A	*Acy1a*	1.06	Up	957452500	903190000
Aminopeptidase B	*Rnpep*	1.16	Up	3195900000	2766150000
AMP deaminase 2	*Ampd2*	1.18	Down	666775000	790005000
AMP deaminase 3	*Ampd3*	1.22	Down	66268000	80873000
Amphiphysin	*Amph*	1.04	Up	4026575000	3876650000
Amyloid-like protein 2	*Aplp2*	1.12	Down	81971000	91614000
Anamorsin	*Ciapin1*	1.04	Down	405500000	422887500
Anion exchange protein 2	*Slc4a2*	1.57	Up	75682500	48292000
Ankyrin-3	*Ank3*	1.01	Down	884227500	894467500
Annexin A1	*Anxa1*	1.07	Down	261662500	279700000
Annexin A2	*Anxa2*	1.01	Up	897677500	887665000
Annexin A3	*Anxa3*	1.03	Up	804742500	778235000
Annexin A5	*Anxa5*	1.18	Up	2443300000	2076975000
Annexin A6	*Anxa6*	1.29	Up	1461050000	1133925000
AP-1 complex subunit beta-1	*Ap1b1*	1.09	Up	3131300000	2875100000
AP-1 complex subunit mu-1	*Ap1m1*	1.11	Up	441590000	399427500
AP-2 complex subunit alpha-2	*Ap2a2*	1.06	Up	13933750000	13094750000
AP-2 complex subunit beta	*Ap2b1*	1.07	Up	11111075000	10372900000
AP-2 complex subunit mu	*Ap2m1*	1.04	Up	5599075000	5363400000
AP-2 complex subunit sigma	*Ap2s1*	1.05	Down	882905000	930420000
AP2-associated protein kinase 1	*Aak1*	1.00	Up	1647175000	1641075000
AP-3 complex subunit mu-2	*Ap3m2*	1.03	Up	784062500	763630000
Apolipoprotein A-I;Proapolipoprotein A-I	*Apoa1*	1.15	Down	82080000	94529250
Apolipoprotein E	*Apoe*	1.01	Down	468312500	474235000
Apoptosis-inducing factor 1, mitochondrial	*Aifm1*	1.02	Up	581260000	571277500
Aquaporin-4	*Aqp4*	1.32	Up	894137500	677605000
ARF GTPase-activating protein GIT1	*Git1*	1.07	Up	98407000	92045666.67
Arfaptin-2	*Arfip2*	1.07	Down	134500000	143614666.7
Arf-GAP domain and FG repeat-containing protein 1	*Agfg1*	1.06	Down	192722500	203992500
Arf-GAP with SH3 domain, ANK repeat and PH domain-containing protein 1	*Asap1*	1.00	Up	125420000	125010000

Arginine and glutamate-rich protein 1	*Arglu1*	1.25	Down	38298000	47883000
Arginine--tRNA ligase, cytoplasmic	*Rars*	1.09	Down	638097500	697227500
Argininosuccinate lyase	*Asl*	1.21	Up	162370000	134703333.3
Argininosuccinate synthase	*Ass1*	1.15	Up	1958175000	1696350000
Armadillo repeat-containing protein 10	*Armc10*	1.13	Up	205442500	181335000
Aryl-hydrocarbon-interacting protein-like 1	*Aipl1*	1.04	Up	788407500	754965000
Asparagine synthetase [glutamine-hydrolyzing]	*Asns*	1.28	Down	284210000	363932500
Aspartate aminotransferase, cytoplasmic	*Got1*	1.00	Down	45113500000	45245250000
Aspartate aminotransferase, mitochondrial	*Got2*	1.02	Up	35240500000	34581000000
Aspartate--tRNA ligase, cytoplasmic	*Dars*	1.01	Up	1910350000	1889125000
Astrocytic phosphoprotein PEA-15	*Pea15*	1.23	Down	297352500	367025000
Ataxin-10	*Atxn10*	1.10	Up	422640000	384730000
Atlastin-1	*Atl1*	1.11	Down	232990000	258257500
ATP synthase F(0) complex subunit B1, mitochondrial	*Atp5f1*	1.10	Up	8043825000	7305850000
ATP synthase protein 8	*Mt-atp8*	1.72	Up	210400000	122442500
ATP synthase subunit a	*Mt-atp6*	1.01	Down	78347000	78871666.67
ATP synthase subunit alpha, mitochondrial	*Atp5a1*	1.07	Up	66813500000	62618750000
ATP synthase subunit beta, mitochondrial	*Atp5b*	1.12	Up	96061750000	85841250000
ATP synthase subunit d, mitochondrial	*Atp5h*	1.01	Down	7077925000	7153500000
ATP synthase subunit e, mitochondrial	*Atp5i*	1.03	Down	1895875000	1949300000
ATP synthase subunit epsilon, mitochondrial	*Atp5e*	1.32	Up	1247230000	942810000
ATP synthase subunit f, mitochondrial	*Atp5j2*	1.06	Up	2201750000	2078800000
ATP synthase subunit g, mitochondrial	*Atp5l*	1.00	Down	3577375000	3591225000
ATP synthase subunit gamma, mitochondrial	*Atp5c1*	1.13	Up	8465225000	7470725000
ATP synthase subunit O, mitochondrial	*Atp5o*	1.10	Up	14240750000	12994500000
ATPase family AAA domain-containing protein 1	*Atad1*	1.02	Up	777360000	759432500
ATPase family AAA domain-containing protein 3	*Atad3*	1.19	Up	142077500	119810000
ATPase inhibitor, mitochondrial	*Atpif1*	1.03	Down	240128000	247592500
ATP-binding cassette sub-family A member 2	*Abca2*	1.78	Down	888925000	1581000000
ATP-binding cassette sub-family B member 7, mitochondrial	*Abcb7*	1.39	Up	80542333.33	57938000
ATP-binding cassette sub-family D member 3	*Abcd3*	1.09	Down	114430000	124610000
ATP-citrate synthase	*Acly*	1.02	Down	9529375000	9746000000
ATP-dependent (S)-NAD(P)H-hydrate dehydratase	*Carkd*	1.05	Up	241316666.7	229980000
ATP-dependent 6-phosphofructokinase, liver type	*Pfkl*	1.04	Up	16649500000	16062500000
ATP-dependent 6-phosphofructokinase, muscle type	*Pfkm*	1.07	Down	4322750000	4612725000
ATP-dependent 6-phosphofructokinase, platelet type	*Pfkp*	1.01	Down	3159375000	3205825000
ATP-dependent DNA helicase Q1	*Recql*	1.36	Down	34025000	46233500
ATP-dependent RNA helicase DDX1	*Ddx1*	1.00	Up	4859825000	4848525000
ATP-dependent RNA helicase SUPV3L1, mitochondrial	*Supv3l1*	1.25	Up	124909000	99926500
ATP-sensitive inward rectifier potassium channel 10	*Kcnj10*	1.11	Up	505680000	457280000
BAG family molecular chaperone regulator 5	*Bag5*	1.00	Down	145776666.7	146023333.3
Band 4.1-like protein 1	*Epb41l1*	1.02	Down	1812350000	1847475000
Band 4.1-like protein 5	*Epb41l5*	1.25	Up	64173000	51371000
Bardet-Biedl syndrome 2 protein homolog	*Bbs2*	1.13	Up	123912666.7	109362333.3
Basic leucine zipper and W2 domain-containing protein 1	*Bzw1*	1.02	Up	127570000	124643333.3

Basic leucine zipper and W2 domain-containing protein 2	*Bzw2*	1.02	Down	419097500	428787500
Basigin	*Bsg*	1.06	Down	3970000000	4193225000
Beta-adducin	*Add2*	1.03	Down	436765000	451895000
Beta-arrestin-1	*Arrb1*	2.07	Up	595772500	288182500
Beta-catenin-like protein 1	*Ctnnbl1*	1.05	Down	204677500	214127500
Beta-crystallin A3;Beta-crystallin A3, isoform A1, Delta4 form;Beta-crystallin A3, isoform A1, Delta7 form;Beta-crystallin A3, isoform A1, Delta8 form	*Cryba1*	1.58	Up	271492500	171631500
Beta-crystallin A4	*Cryba4*	3.39	Up	85191750	25144500
Beta-crystallin B2	*Crybb2*	1.89	Up	1062927500	561807500
Beta-crystallin S	*Crygs*	1.68	Up	107755000	64106750
Beta-lactamase-like protein 2	*Lactb2*	1.14	Up	101045750	88720500
Beta-soluble NSF attachment protein	*Napb*	1.01	Up	3149450000	3121925000
Beta-synuclein	*Sncb*	1.16	Down	5375875000	6251275000
Bifunctional ATP-dependent dihydroxyacetone kinase/FAD-AMP lyase (cyclizing);ATP-dependent dihydroxyacetone kinase;FAD-AMP lyase (cyclizing)	*Dak*	1.06	Up	116835000	110307250
Bifunctional purine biosynthesis protein PURH;Phosphoribosylaminoimidazolecarboxamide formyltransferase;IMP cyclohydrolase	*Atic*	1.05	Up	2317675000	2205125000
Biliverdin reductase A	*Blvra*	1.05	Up	566887500	540447500
Bis(5-adenosyl)-triphosphatase	*Fhit*	1.14	Down	43029000	48936000
Bis(5-nucleosyl)-tetraphosphatase [asymmetrical]	*Nudt2*	1.06	Up	535497500	503367500
Bleomycin hydrolase	*Blmh*	1.23	Down	895977500	1105300000
Brain acid soluble protein 1	*Basp1*	1.42	Up	542803333.3	382897500
Branched-chain-amino-acid aminotransferase, cytosolic	*Bcat1*	1.02	Up	4813250000	4704575000
Brefeldin A-inhibited guanine nucleotide-exchange protein 1	*Arfgef1*	1.15	Up	97820000	85315250
Brefeldin A-inhibited guanine nucleotide-exchange protein 2	*Arfgef2*	1.22	Up	151195000	124415000
C-1-tetrahydrofolate synthase, cytoplasmic;Methylenetetrahydrofolate dehydrogenase;Methenyltetrahydrofolate cyclohydrolase;Formyltetrahydrofolate synthetase;C-1-tetrahydrofolate synthase, cytoplasmic, N-terminally processed	*Mthfd1*	1.09	Down	510312500	556932500
Cadherin-2	*Cdh2*	1.03	Up	605437500	589670000
Calbindin	*Calb1*	1.02	Up	1007465000	983677500
Calcium/calmodulin-dependent 3,5-cyclic nucleotide phosphodiesterase 1B	*Pde1b*	1.19	Up	265570000	222352500
Calcium/calmodulin-dependent protein kinase type 1	*Camk1*	1.01	Down	182242500	184892500
Calcium/calmodulin-dependent protein kinase type II subunit alpha	*Camk2a*	1.12	Up	430585000	386097500
Calcium/calmodulin-dependent protein kinase type II subunit beta	*Camk2b*	1.01	Up	2813475000	2798000000
Calcium/calmodulin-dependent protein kinase type II subunit delta	*Camk2d*	1.00	Down	8519400000	8553750000
Calcium/calmodulin-dependent protein kinase type II subunit gamma	*Camk2g*	1.17	Up	337736666.7	288540000
Calcium/calmodulin-dependent protein kinase type IV	*Camk4*	1.44	Down	30930000	44561750
Calcium-binding mitochondrial carrier protein SCaMC-2	*Slc25a25*	1.56	Up	108535333.3	69402000
Calcium-binding protein 1	*Cabp1*	1.37	Down	4665200	6370033.333
Calcium-dependent secretion activator 1	*Cadps*	1.03	Up	7112875000	6897350000
Calcyclin-binding protein	*Cacybp*	1.03	Down	1482100000	1521750000

Calmodulin	*Calm1*	1.01	Down	3273650000	3316400000
Calnexin	*Canx*	1.11	Up	7445525000	6682475000
Calpain small subunit 1	*Capns1*	1.03	Down	286240000	295207500
Calpain-2 catalytic subunit	*Capn2*	1.07	Up	602855000	562822500
Calponin-3	*Cnn3*	1.04	Down	601487500	623987500
Calreticulin	*Calr*	1.08	Up	4982675000	4622875000
Calretinin	*Calb2*	1.06	Down	18712750000	19831000000
Calsyntenin-1;Soluble Alc-alpha;CTF1-alpha	*Clstn1*	1.25	Up	68524000	54684500
CaM kinase-like vesicle-associated protein	*Camkv*	1.05	Up	2099625000	1995500000
cAMP-dependent protein kinase catalytic subunit alpha	*Prkaca*	1.04	Up	728912500	701215000
cAMP-dependent protein kinase catalytic subunit beta	*Prkacb*	1.03	Down	2213500000	2270525000
cAMP-dependent protein kinase type I-alpha regulatory subunit;cAMP-dependent protein kinase type I-alpha regulatory subunit, N-terminally processed	*Prkar1a*	1.03	Up	1200962500	1167400000
cAMP-dependent protein kinase type I-beta regulatory subunit	*Prkar1b*	1.37	Down	62355000	85529000
cAMP-dependent protein kinase type II-alpha regulatory subunit	*Prkar2a*	1.11	Up	4322750000	3902850000
cAMP-dependent protein kinase type II-beta regulatory subunit	*Prkar2b*	1.05	Up	934920000	888635000
CAP-Gly domain-containing linker protein 2	*Clip2*	1.05	Up	236885000	226520000
Caprin-1	*Caprin1*	1.01	Up	370625000	367505000
Cap-specific mRNA (nucleoside-2-O-)-methyltransferase 1	*Cmtr1*	1.05	Up	150460000	142640000
Carbonic anhydrase 1	*Ca1*	1.10	Down	150765000	166310000
Carbonic anhydrase 2	*Ca2*	1.00	Up	52683750000	52562000000
Carbonic anhydrase-related protein	*Ca8*	1.06	Up	93837000	88660000
Carbonyl reductase [NADPH] 1	*Cbr1*	1.08	Up	6000800000	5560425000
Carboxymethylenebutenolidase homolog	*Cmbl*	1.12	Up	109515000	97795000
Carboxypeptidase E	*Cpe*	1.10	Up	271400000	247690000
Carnitine O-acetyltransferase	*Crat*	1.04	Up	145940000	140482500
Carnitine O-palmitoyltransferase 1, liver isoform	*Cpt1a*	1.13	Up	192722500	170445000
Carnitine O-palmitoyltransferase 2, mitochondrial	*Cpt2*	1.11	Up	1232327500	1107037500
Casein kinase I isoform alpha	*Csnk1a1*	1.17	Down	127685000	148825000
Casein kinase I isoform delta	*Csnk1d*	2.17	Down	10006000	21690000
Casein kinase I isoform gamma-2;Casein kinase I isoform gamma-3;Casein kinase I isoform gamma-1	*Csnk1g2;Csnk1g3;Csnk1g1*	1.40	Up	116216666.7	83216333.33
Casein kinase II subunit alpha	*Csnk2a1*	1.01	Down	2880700000	2899725000
Casein kinase II subunit beta	*Csnk2b*	1.34	Down	902935000	1213875000
Caskin-1	*Caskin1*	1.03	Up	790285000	770755000
Catalase	*Cat*	1.32	Down	206920000	272392500
Catechol O-methyltransferase	*Comt*	1.06	Up	379535000	359157500
Catenin beta-1	*Ctnnb1*	1.01	Up	2928150000	2894900000
Cathepsin B;Cathepsin B light chain;Cathepsin B heavy chain	*Ctsb*	1.10	Down	694257500	765582500
Cathepsin D;Cathepsin D 12 kDa light chain;Cathepsin D 9 kDa light chain;Cathepsin D 34 kDa heavy chain;Cathepsin D 30 kDa heavy chain	*Ctsd*	1.02	Up	3606550000	3540900000
Caveolin-1	*Cav1*	1.03	Up	979492500	952035000
CB1 cannabinoid receptor-interacting protein 1	*Cnrip1*	1.02	Down	1809275000	1851825000
CD166 antigen	*Alcam*	1.18	Up	287600000	243542500
CD44 antigen	*Cd44*	1.14	Up	1259900000	1103585000
CD59 glycoprotein	*Cd59*	1.17	Down	479047500	560947500

CD81 antigen	*Cd81*	1.08	Down	529450000	573296666.7
CD9 antigen	*Cd9*	1.22	Up	891910000	729085000
CDGSH iron-sulfur domain-containing protein 1	*Cisd1*	1.02	Up	2465825000	2407000000
CDK5 regulatory subunit-associated protein 3	*Cdk5rap3*	1.19	Down	92408500	109740000
CDP-diacylglycerol--inositol 3-phosphatidyltransferase	*Cdipt*	1.07	Up	567880000	531367500
Cell adhesion molecule 2	*Cadm2*	1.05	Up	1804225000	1721200000
Cell adhesion molecule 3	*Cadm3*	1.10	Up	980950000	890287500
Cell adhesion molecule 4	*Cadm4*	1.10	Up	123832250	112748500
Cell cycle exit and neuronal differentiation protein 1	*Cend1*	1.17	Down	140577333.3	164780000
Cell division control protein 42 homolog	*Cdc42*	1.04	Up	2110225000	2021000000
Cell division cycle 5-like protein	*Cdc5l*	1.03	Up	226080000	219477500
Cell surface glycoprotein MUC18	*Mcam*	1.14	Up	117760000	103295000
Cellular nucleic acid-binding protein	*Cnbp*	1.54	Down	203021250	312617500
Cellular retinoic acid-binding protein 1	*Crabp1*	1.06	Down	1966075000	2090525000
Ceruloplasmin	*Cp*	1.15	Up	3074125000	2683400000
cGMP-gated cation channel alpha-1	*Cnga1*	1.15	Up	652675000	566775000
cGMP-inhibited 3,5-cyclic phosphodiesterase A	*Pde3a*	1.06	Down	92535250	97839500
Chitinase domain-containing protein 1	*Chid1*	1.09	Down	167870000	182767500
Chloride intracellular channel protein 1	*Clic1*	1.06	Down	562390000	593572500
Chloride intracellular channel protein 4	*Clic4*	1.39	Down	95956000	133440000
Chloride intracellular channel protein 6	*Clic6*	1.05	Down	93259000	97939000
Choline dehydrogenase, mitochondrial	*Chdh*	1.21	Down	129197500	155720000
Choline O-acetyltransferase	*Chat*	1.22	Down	104663666.7	127852500
Choline transporter-like protein 2	*Slc44a2*	1.06	Down	289837500	308567500
Choline/ethanolamine kinase	*Chkb*	1.02	Up	180790000	176460000
Chromodomain-helicase-DNA-binding protein 5	*Chd5*	1.12	Up	297210000	266305000
Cilia- and flagella-associated protein 36	*Cfap36*	1.33	Up	98955000	74481000
Ciliary neurotrophic factor	*Cntf*	1.00	Up	155580000	155373333.3
Ciliary neurotrophic factor receptor subunit alpha	*Cntfr*	1.27	Up	245292500	193467500
Citrate synthase, mitochondrial	*Cs*	1.08	Down	16584250000	17968500000
Clathrin coat assembly protein AP180	*Snap91*	1.00	Up	6890550000	6882075000
Clathrin heavy chain 1	*Cltc*	1.07	Up	80309000000	75261500000
Clathrin light chain A	*Clta*	1.77	Up	846083333.3	478195000
Clathrin light chain B	*Cltb*	1.11	Up	601797500	544375000
Cleavage and polyadenylation specificity factor subunit 5	*Nudt21*	1.19	Up	906427500	764075000
Cleavage and polyadenylation specificity factor subunit 7	*Cpsf7*	1.17	Up	115658000	99227750
Cleavage stimulation factor subunit 1	*Cstf1*	1.40	Down	49357000	68873000
CLIP-associating protein 2	*Clasp2*	1.06	Down	863222500	912397500
Clusterin;Clusterin beta chain;Clusterin alpha chain	*Clu*	1.91	Up	189795000	99412500
Coactosin-like protein	*Cotl1*	1.04	Up	1359675000	1305625000
Coatomer subunit beta	*Copb1*	1.06	Up	1112777500	1051637500
Coatomer subunit beta	*Copb2*	1.01	Down	1691000000	1702775000
Coatomer subunit delta	*Arcn1*	1.08	Down	661377500	715052500
Coatomer subunit gamma-1	*Copg1*	1.01	Down	397395000	400800000
Coatomer subunit gamma-2	*Copg2*	1.05	Up	557542500	530562500
Cocaine- and amphetamine-regulated transcript protein;CART(1-52);CART(55-102);CART(62-102)	*Cartpt*	1.05	Down	159690500	167835000
Cofilin-1	*Cfl1*	1.03	Up	11618250000	11298750000
Coiled-coil domain-containing protein 127	*Ccdc127*	1.31	Up	9697200	7405500
Coiled-coil domain-containing protein 47	*Ccdc47*	1.06	Down	150742500	160225000
Coiled-coil domain-containing protein 51	*Ccdc51*	1.08	Down	151900000	164422500
Coiled-coil domain-containing protein 93	*Ccdc93*	1.29	Down	42769000	55190666.67

Cold shock domain-containing protein E1	*Csde1*	1.22	Down	115406250	141222500
Cold-inducible RNA-binding protein	*Cirbp*	1.06	Down	1392280000	1473225000
Complement C3;Complement C3 beta chain;C3-beta-c;Complement C3 alpha chain;C3a anaphylatoxin;Acylation stimulating protein;Complement C3b alpha chain;Complement C3c alpha chain fragment 1;Complement C3dg fragment;Complement C3g fragment;Complement C3d fragment;Complement C3f fragment;Complement C3c alpha chain fragment 2	*C3*	1.11	Down	478977500	532495000
Complement component 1 Q subcomponent-binding protein, mitochondrial	*C1qbp*	1.14	Down	847745000	966745000
Complement component receptor 1-like protein	*Cr1l*	1.07	Down	106438250	113722750
Contactin-1	*Cntn1*	1.10	Down	2068550000	2279975000
Contactin-associated protein 1	*Cntnap1*	1.19	Up	221666666.7	187025000
COP9 signalosome complex subunit 1	*Gps1*	1.04	Down	944920000	985872500
COP9 signalosome complex subunit 2	*Cops2*	1.08	Up	1188605000	1100715000
COP9 signalosome complex subunit 3	*Cops3*	1.01	Down	846855000	853530000
COP9 signalosome complex subunit 4	*Cops4*	1.08	Up	1805575000	1665375000
COP9 signalosome complex subunit 8	*Cops8*	1.11	Down	302740000	336912500
Copine-9	*Cpne9*	1.12	Up	387710000	344850000
Copper chaperone for superoxide dismutase	*Ccs*	1.21	Down	152756666.7	184712500
Copper transport protein ATOX1	*Atox1*	1.01	Down	309475000	313915000
Coronin-1B	*Coro1b*	1.05	Down	623502500	656140000
Coronin-7	*Coro7*	1.09	Down	136747500	149445000
Craniofacial development protein 1	*Cfdp1*	1.05	Up	105670000	101062000
Creatine kinase B-type	*Ckb*	1.00	Up	92798000000	92772500000
Creatine kinase M-type	*Ckm*	1.70	Up	641130000	377383333.3
Creatine kinase U-type, mitochondrial	*Ckmt1*	1.05	Up	9006550000	8593375000
Crooked neck-like protein 1	*Crnkl1*	1.10	Up	1095000000	998220000
C-terminal-binding protein 1	*Ctbp1*	1.03	Up	1470275000	1426175000
C-terminal-binding protein 2	*Ctbp2*	1.22	Up	926720000	759195000
CTP synthase 2	*Ctps2*	1.03	Down	85330666.67	88178000
CUGBP Elav-like family member 1	*Celf1*	1.11	Up	171331500	154957500
CUGBP Elav-like family member 2	*Celf2*	1.11	Up	1238950000	1120375000
Cullin-3	*Cul3*	1.07	Down	1024205000	1098125000
Cullin-5	*Cul5*	1.06	Down	611385000	648687500
Cullin-associated NEDD8-dissociated protein 1	*Cand1*	1.07	Up	10681500000	9965400000
Cyclin-dependent kinase 17	*Cdk17*	1.48	Up	33591000	22647000
Cyclin-dependent-like kinase 5	*Cdk5*	1.09	Down	717620000	779000000
Cyclin-G-associated kinase	*Gak*	1.33	Up	275375000	207670000
Cystatin-B	*Cstb*	1.08	Up	654907500	608227500
Cysteine and glycine-rich protein 1	*Csrp1*	1.13	Up	494517500	439120000
Cysteine and glycine-rich protein 2	*Csrp2*	1.03	Up	63305000	61370000
Cysteine and histidine-rich domain-containing protein 1	*Chordc1*	1.17	Up	106470000	91174500
Cysteine desulfurase, mitochondrial	*Nfs1*	1.39	Down	112246666.7	155570000
Cysteine sulfinic acid decarboxylase	*Csad*	1.08	Down	337890000	364720000
Cysteine-rich protein 2	*Crip2*	1.10	Down	813660000	893795000
Cysteine-rich with EGF-like domain protein 1	*Creld1*	1.07	Down	53613000	57212500
Cytochrome b	*Mt-Cyb*	1.24	Up	139770000	113110000
Cytochrome b5	*Cyb5a*	1.01	Down	215947500	217137500
Cytochrome b5 type B	*Cyb5b*	1.02	Down	609345000	621447500
Cytochrome b-c1 complex subunit 1, mitochondrial	*Uqcrc1*	1.05	Up	4884900000	4670950000

Cytochrome b-c1 complex subunit 2, mitochondrial	*Uqcrc2*	1.01	Down	10668550000	10728775000
Cytochrome b-c1 complex subunit 6, mitochondrial	*Uqcrh*	1.07	Up	361790000	338215000
Cytochrome b-c1 complex subunit 8	*Uqcrq*	1.04	Up	1463900000	1406275000
Cytochrome b-c1 complex subunit Rieske, mitochondrial;Cytochrome b-c1 complex subunit 11	*Uqcrfs1*	1.06	Down	1416550000	1503875000
Cytochrome c oxidase subunit 1	*Mtco1*	1.29	Up	542560000	420583333.3
Cytochrome c oxidase subunit 2	*Mtco2*	1.06	Up	4976925000	4712325000
Cytochrome c oxidase subunit 3	*Mtco3*	1.18	Up	151855333.3	129183500
Cytochrome c oxidase subunit 4 isoform 1, mitochondrial	*Cox4i1*	1.30	Down	5322275000	6912550000
Cytochrome c oxidase subunit 4 isoform 2, mitochondrial	*Cox4i2*	1.26	Up	348440000	275582500
Cytochrome c oxidase subunit 5A, mitochondrial	*Cox5a*	1.02	Down	4050500000	4145125000
Cytochrome c oxidase subunit 5B, mitochondrial	*Cox5b*	1.00	Up	1596275000	1593275000
Cytochrome c oxidase subunit 6A1, mitochondrial	*Cox6a1*	1.02	Up	927206666.7	905800000
Cytochrome c oxidase subunit 6C-2	*Cox6c2*	1.22	Down	1671875000	2035775000
Cytochrome c oxidase subunit 7A2, mitochondrial	*Cox7a2*	1.18	Up	580072500	492022500
Cytochrome c, somatic	*Cycs*	1.07	Up	5842925000	5455900000
Cytoglobin	*Cygb*	1.09	Down	1207050000	1309950000
Cytoplasmic aconitate hydratase	*Aco1*	1.06	Up	1634425000	1542725000
Cytoplasmic dynein 1 heavy chain 1	*Dync1h1*	1.07	Up	34955250000	32685750000
Cytoplasmic dynein 1 intermediate chain 1	*Dync1i1*	1.37	Up	132340000	96435500
Cytoplasmic dynein 1 intermediate chain 2	*Dync1i2*	1.12	Up	465702500	414400000
Cytoplasmic dynein 1 light intermediate chain 1	*Dync1li1*	1.13	Down	1065242500	1203450000
Cytoplasmic dynein 1 light intermediate chain 2	*Dync1li2*	1.10	Down	242553500	267887500
Cytoplasmic dynein 2 heavy chain 1	*Dync2h1*	1.47	Down	132841500	195246666.7
Cytosol aminopeptidase	*Lap3*	1.12	Up	2099200000	1873325000
Cytosolic acyl coenzyme A thioester hydrolase	*Acot7*	1.02	Down	2055200000	2095475000
Cytosolic non-specific dipeptidase	*Cndp2*	1.21	Down	1931625000	2346150000
D-3-phosphoglycerate dehydrogenase	*Phgdh*	1.19	Up	643742500	539642500
D-beta-hydroxybutyrate dehydrogenase, mitochondrial	*Bdh1*	1.10	Up	599750000	546857500
DDB1- and CUL4-associated factor 8	*Dcaf8*	1.09	Up	75405000	69174666.67
D-dopachrome decarboxylase	*Ddt*	1.09	Down	2498975000	2716750000
Delta(3,5)-Delta(2,4)-dienoyl-CoA isomerase, mitochondrial	*Ech1*	1.20	Up	153865000	127990000
Delta-1-pyrroline-5-carboxylate dehydrogenase, mitochondrial	*Aldh4a1*	1.11	Down	648040000	716342500
Delta-aminolevulinic acid dehydratase	*Alad*	1.13	Up	362032500	320477500
Deoxyhypusine synthase	*Dhps*	1.41	Up	61238500	43553000
Dephospho-CoA kinase domain-containing protein	*Dcakd*	1.30	Down	21367000	27695500
Destrin	*Dstn*	1.14	Up	2849375000	2506300000
Deubiquitinating protein VCIP135	*Vcpip1*	1.25	Down	87165000	108924250
Diacylglycerol kinase zeta	*Dgkz*	1.08	Up	45510500	42246500
Dihydrolipoyl dehydrogenase, mitochondrial	*Dld*	1.03	Up	4284775000	4156875000
Dihydrolipoyllysine-residue acetyltransferase component of pyruvate dehydrogenase complex, mitochondrial	*Dlat*	1.09	Up	5067750000	4630450000
Dihydrolipoyllysine-residue succinyltransferase component of 2-oxoglutarate dehydrogenase complex, mitochondrial	*Dlst*	1.04	Up	1628725000	1569525000

Dihydropteridine reductase	*Qdpr*	1.08	Down	1921250000	2072125000
Dihydropyrimidinase-related protein 1	*Crmp1*	1.02	Down	9807025000	9972525000
Dihydropyrimidinase-related protein 2	*Dpysl2*	1.01	Up	68687250000	68087250000
Dihydropyrimidinase-related protein 3	*Dpysl3*	1.01	Up	8753425000	8670625000
Dihydropyrimidinase-related protein 4	*Dpysl4*	1.02	Up	19762500000	19328750000
Dihydropyrimidinase-related protein 5	*Dpysl5*	1.01	Down	10870950000	10985575000
Dipeptidyl aminopeptidase-like protein 6	*Dpp6*	1.17	Down	635902500	745355000
Dipeptidyl peptidase 3	*Dpp3*	1.06	Up	718152500	680207500
Diphosphoinositol polyphosphate phosphohydrolase 1	*Nudt3*	1.28	Down	273140000	350660000
Diphosphoinositol polyphosphate phosphohydrolase 2	*Nudt4*	1.51	Up	59385000	39292000
Diphosphomevalonate decarboxylase	*Mvd*	1.03	Up	133126666.7	129473333.3
Disabled homolog 2-interacting protein	*Dab2ip*	1.16	Up	113310000	97827250
Disks large homolog 1	*Dlg1*	1.26	Up	226430000	179730000
Disks large homolog 2	*Dlg2*	1.04	Up	2162450000	2087700000
Disks large homolog 3	*Dlg3*	1.19	Up	99968000	83712000
Disks large homolog 4	*Dlg4*	1.07	Up	1278925000	1195350000
DNA damage-binding protein 1	*Ddb1*	1.03	Up	3115475000	3031200000
DNA repair protein XRCC1	*Xrcc1*	1.15	Up	25060000	21846000
DNA topoisomerase 1	*Top1*	1.52	Up	57442000	37900000
DNA topoisomerase 2-alpha	*Top2a*	1.81	Up	152155000	83870333.33
DNA-(apurinic or apyrimidinic site) lyase;DNA-(apurinic or apyrimidinic site) lyase, mitochondrial	*Apex1*	1.03	Down	1779650000	1826375000
DnaJ homolog subfamily A member 1	*Dnaja1*	1.06	Up	587180000	553777500
DnaJ homolog subfamily A member 2	*Dnaja2*	1.13	Down	848457500	959552500
DnaJ homolog subfamily B member 11	*Dnajb11*	1.23	Down	266510000	328356666.7
DnaJ homolog subfamily C member 5	*Dnajc5*	1.06	Down	2786875000	2947525000
DnaJ homolog subfamily C member 8	*Dnajc8*	1.41	Down	234242500	330770000
Dolichyl-diphosphooligosaccharide--protein glycosyltransferase 48 kDa subunit	*Ddost*	1.15	Up	1128070000	976770000
Dolichyl-diphosphooligosaccharide--protein glycosyltransferase subunit 1	*Rpn1*	1.06	Up	2247975000	2120925000
Dolichyl-diphosphooligosaccharide--protein glycosyltransferase subunit 2	*Rpn2*	1.01	Up	1059145000	1047920000
Drebrin	*Dbn1*	1.38	Down	508357500	699162500
Drebrin-like protein	*Dbnl*	1.06	Down	381267500	402685000
Dual specificity mitogen-activated protein kinase kinase 1	*Map2k1*	1.06	Down	1030570000	1090725000
Dual specificity mitogen-activated protein kinase kinase 2	*Map2k2*	1.09	Down	263075000	286335000
Dual specificity tyrosine-phosphorylation-regulated kinase 1A	*Dyrk1a*	1.60	Down	74864000	119740000
Dynactin subunit 1	*Dctn1*	1.03	Up	2989975000	2898725000
Dynactin subunit 2	*Dctn2*	1.01	Down	3579775000	3616050000
Dynactin subunit 4	*Dctn4*	1.16	Up	528265000	453982500
Dynamin-1	*Dnm1*	1.04	Up	20937750000	20104000000
Dynamin-1-like protein	*Dnm1l*	1.06	Up	3076200000	2891225000
Dynamin-2	*Dnm2*	1.22	Up	1169297500	955762500
Dynamin-3	*Dnm3*	1.07	Up	1858275000	1744125000
Dynamin-like 120 kDa protein, mitochondrial;Dynamin-like 120 kDa protein, form S1	*Opa1*	1.03	Up	1278050000	1243450000
Dynein light chain 2, cytoplasmic	*Dynll2*	1.05	Up	3022000000	2874600000
Dynein light chain roadblock-type 1	*Dynlrb1*	1.17	Down	402870000	469890000
Dystrophin	*Dmd*	1.36	Up	330260000	243010000
E3 ubiquitin-protein ligase BRE1B	*Rnf40*	1.03	Up	107166666.7	103698000
E3 ubiquitin-protein ligase NEDD4	*Nedd4*	1.05	Down	1102540000	1159150000
E3 ubiquitin-protein ligase RNF123	*Rnf123*	1.09	Up	65552000	60024000
E3 ubiquitin-protein ligase UBR4	*Ubr4*	1.17	Down	353000000	411497500
E3 UFM1-protein ligase 1	*Ufl1*	1.10	Up	97281000	88604666.67

Echinoderm microtubule-associated protein-like 1	*Eml1*	1.03	Down	74647000	77119000
Echinoderm microtubule-associated protein-like 2	*Eml2*	1.06	Down	284425000	302717500
Ectonucleoside triphosphate diphosphohydrolase 2	*Entpd2*	1.32	Down	30518000	40215666.67
Ectonucleotide pyrophosphatase/phosphodiesterase family member 5	*Enpp5*	1.21	Up	221680000	183565000
EF-hand domain-containing protein D2	*Efhd2*	1.09	Down	557167500	605327500
EH domain-containing protein 1	*Ehd1*	1.02	Up	1676475000	1640625000
EH domain-containing protein 3	*Ehd3*	1.09	Down	466940000	509670000
ELAV-like protein 4	*Elavl4*	1.30	Down	293995000	382092500
Electron transfer flavoprotein subunit alpha, mitochondrial	*Etfa*	1.03	Up	1752150000	1697900000
Electron transfer flavoprotein subunit beta	*Etfb*	1.08	Up	721117500	664887500
Electron transfer flavoprotein-ubiquinone oxidoreductase, mitochondrial	*Etfdh*	1.66	Up	133353333.3	80505000
Elongation factor 1-alpha 1	*Eef1a1*	1.01	Down	30600000000	30839750000
Elongation factor 1-alpha 2	*Eef1a2*	1.07	Up	4201575000	3934950000
Elongation factor 1-delta	*Eef1d*	1.08	Down	1107050000	1195900000
Elongation factor 1-gamma	*Eef1g*	1.05	Up	4511200000	4291800000
Elongation factor 2	*Eef2*	1.01	Down	10288150000	10419325000
Elongation factor Tu, mitochondrial	*Tufm*	1.05	Up	4241075000	4020275000
Elongator complex protein 1	*Ikbkap*	1.10	Up	980387500	887767500
Elongator complex protein 2	*Elp2*	1.03	Down	212246666.7	217995000
Embigin	*Emb*	1.11	Down	191885000	212682500
Endophilin-A1	*Sh3gl2*	1.03	Up	4931150000	4791525000
Endophilin-A2	*Sh3gl1*	1.04	Up	151630000	146410000
Endophilin-B1	*Sh3glb1*	1.02	Down	131373333.3	133939500
Endophilin-B2	*Sh3glb2*	1.01	Up	508257500	502560000
Endoplasmic reticulum resident protein 29	*Erp29*	1.03	Up	621775000	602942500
Endoplasmin	*Hsp90b1*	1.05	Up	6463950000	6165925000
Endothelial differentiation-related factor 1	*Edf1*	1.08	Down	210013333.3	227110000
Enhancer of mRNA-decapping protein 4	*Edc4*	1.01	Down	140307000	141517500
Enolase-phosphatase E1	*Enoph1*	1.03	Down	314462500	323360000
Enoyl-CoA delta isomerase 1, mitochondrial	*Eci1*	1.01	Down	837167500	848575000
Enoyl-CoA delta isomerase 2, mitochondrial	*Eci2*	1.03	Down	164162500	169200000
Enoyl-CoA hydratase, mitochondrial	*Echs1*	1.01	Down	1292200000	1308550000
Ephrin type-B receptor 1;Ephrin type-A receptor 6	*Ephb1;Epha6*	1.38	Up	243163333.3	176490000
Epsin-1	*Epn1*	1.05	Up	360197500	341900000
ER membrane protein complex subunit 2	*Emc2*	1.17	Down	208270000	243205000
ER membrane protein complex subunit 3	*Emc3*	1.15	Up	130300000	113208333.3
ER membrane protein complex subunit 8	*Emc8*	1.27	Up	271150000	213530000
Erlin-2	*Erlin2*	1.04	Up	1866125000	1800400000
ERO1-like protein alpha	*Ero1l*	1.16	Down	155325000	180886666.7
ES1 protein homolog, mitochondrial	*C21orf33*	1.01	Up	2076875000	2066175000
Ester hydrolase C11orf54 homolog	*N/A*	1.47	Down	371655000	546245000
Estradiol 17-beta-dehydrogenase 8	*Hsd17b8*	1.16	Down	40091000	46445666.67
Ethanolamine-phosphate cytidylyltransferase	*Pcyt2*	1.02	Up	117134750	115013500
Ethylmalonyl-CoA decarboxylase	*Echdc1*	1.28	Up	175870000	137840000
Eukaryotic initiation factor 4A-II;Eukaryotic initiation factor 4A-II, N-terminally processed	*Eif4a2*	1.00	Down	6176475000	6200725000
Eukaryotic initiation factor 4A-III;Eukaryotic initiation factor 4A-III, N-terminally processed	*Eif4a3*	1.14	Down	1543950000	1766300000
Eukaryotic peptide chain release factor subunit 1	*Etf1*	1.06	Up	121140000	114486666.7

Eukaryotic translation initiation factor 1A	*Eif1a*	1.67	Up	102425500	61270000
Eukaryotic translation initiation factor 2 subunit 1	*Eif2s1*	1.02	Down	565380000	574070000
Eukaryotic translation initiation factor 2 subunit 3	*Eif2s3*	1.13	Down	853010000	960195000
Eukaryotic translation initiation factor 3 subunit A	*Eif3a*	1.07	Up	1370625000	1283300000
Eukaryotic translation initiation factor 3 subunit B	*Eif3b*	1.00	Down	569387500	570860000
Eukaryotic translation initiation factor 3 subunit C	*Eif3c*	1.04	Down	742740000	774672500
Eukaryotic translation initiation factor 3 subunit D	*Eif3d*	1.35	Up	411957500	304757500
Eukaryotic translation initiation factor 3 subunit E	*Eif3e*	1.06	Up	567937500	537272500
Eukaryotic translation initiation factor 3 subunit G	*Eif3g*	1.12	Up	202360000	181340000
Eukaryotic translation initiation factor 3 subunit H	*Eif3h*	1.10	Down	185925000	205422500
Eukaryotic translation initiation factor 3 subunit I	*Eif3i*	1.05	Up	432502500	412465000
Eukaryotic translation initiation factor 4E	*Eif4e*	1.01	Up	570132500	563075000
Eukaryotic translation initiation factor 4H	*Eif4h*	1.32	Down	586937500	776885000
Eukaryotic translation initiation factor 5	*Eif5*	1.03	Up	269870000	261230000
Eukaryotic translation initiation factor 5A-1	*Eif5a*	1.06	Up	1855425000	1743200000
Eukaryotic translation initiation factor 6	*Eif6*	1.04	Down	188422500	195895000
Excitatory amino acid transporter 1	*Slc1a3*	1.02	Up	5637925000	5547800000
Exocyst complex component 2	*Exoc2*	1.06	Down	69918000	74023500
Exportin-1	*Xpo1*	1.10	Up	3587775000	3271650000
Extended synaptotagmin-1	*Esyt1*	1.02	Up	129497500	126890000
Ezrin	*Ezr*	1.28	Down	558290000	716712500
F-actin-capping protein subunit alpha-1	*Capza1*	1.09	Up	385023333.3	353942500
F-actin-capping protein subunit alpha-2	*Capza2*	1.07	Down	1644400000	1757475000
F-actin-capping protein subunit beta	*Capzb*	1.26	Down	2110025000	2663500000
Far upstream element-binding protein 1	*Fubp1*	1.09	Down	1201522500	1311100000
Far upstream element-binding protein 2	*Khsrp*	1.11	Down	4186525000	4638725000
Farnesyl pyrophosphate synthase	*Fdps*	1.11	Up	1007287500	907285000
FAS-associated factor 2	*Faf2*	1.05	Up	230755000	219447500
Fascin	*Fscn1*	1.17	Down	4726950000	5516200000
Fatty acid synthase;[Acyl-carrier-protein] S-acetyltransferase;[Acyl-carrier-protein] S-malonyltransferase;3-oxoacyl-[acyl-carrier-protein] synthase;3-oxoacyl-[acyl-carrier-protein] reductase;3-hydroxyacyl-[acyl-carrier-protein] dehydratase;Enoyl-[acyl-carrier-protein] reductase;Oleoyl-[acyl-carrier-protein] hydrolase	*Fasn*	1.04	Up	5155000000	4973700000
Fatty acid-binding protein, epidermal	*Fabp5*	1.24	Up	3915750000	3154100000
Fatty acid-binding protein, heart	*Fabp3*	1.00	Up	800862500	799795000
Fatty aldehyde dehydrogenase	*Aldh3a2*	1.08	Up	1538275000	1424325000
Fatty-acid amide hydrolase 1	*Faah*	1.18	Up	115700000	98455000
F-box only protein 6	*Fbxo6*	1.13	Up	351442500	311037500
FERM, RhoGEF and pleckstrin domain-containing protein 1	*Farp1*	1.00	Down	77464000	77742500
Ferritin heavy chain;Ferritin heavy chain, N-terminally processed	*Fth1*	1.02	Down	1071757500	1094730000
Ferritin light chain 1;Protein FAM65B	*Ftl1;Fam65b*	1.28	Up	1986175000	1546800000
Fibrinogen alpha chain;Fibrinopeptide A;Fibrinogen alpha chain	*Fga*	1.86	Down	65288500	121578750
Fibrinogen beta chain;Fibrinopeptide B;Fibrinogen beta chain	*Fgb*	1.42	Down	67483000	96078000
Fibrinogen gamma chain	*Fgg*	1.45	Down	94733000	136922500
Fibroblast growth factor 2	*Fgf2*	1.37	Up	230947500	168823333.3

Filamin-C	*Flnc*	1.55	Up	222650000	143610000
Flap endonuclease 1	*Fen1*	1.12	Up	756302500	678247500
Four and a half LIM domains protein 1	*Fhl1*	1.13	Up	648537500	572940000
Fragile X mental retardation protein 1 homolog	*Fmr1*	1.15	Up	145952500	126827500
Fructose-bisphosphate aldolase A	*Aldoa*	1.12	Down	54270250000	60619500000
Fructose-bisphosphate aldolase C	*Aldoc*	1.02	Down	35491000000	36365500000
Fumarate hydratase, mitochondrial	*Fh*	1.05	Up	2440550000	2321325000
Fumarylacetoacetate hydrolase domain-containing protein 2	*Fahd2*	1.06	Up	1061852500	999592500
FUN14 domain-containing protein 1	*Fundc1*	1.26	Down	40045000	50572000
G protein-activated inward rectifier potassium channel 4	*Kcnj5*	1.22	Up	33148000	27097000
Galectin-1	*Lgals1*	1.12	Down	891037500	995640000
Gamma-adducin	*Add3*	1.03	Down	1273317500	1307250000
Gamma-aminobutyric acid receptor subunit alpha-1	*Gabra1*	1.26	Down	122369500	154087500
Gamma-aminobutyric acid receptor subunit beta-3	*Gabrb3*	1.27	Up	314863333.3	247365000
Gamma-aminobutyric acid receptor subunit gamma-2	*Gabrg2*	1.14	Up	142395000	124815000
Gamma-aminobutyric acid receptor-associated protein-like 2	*Gabarapl2*	1.03	Up	106259000	103305500
Gamma-aminobutyric acid type B receptor subunit 1	*Gabbr1*	1.36	Down	76157000	103348000
Gamma-crystallin B	*Crygb*	1.70	Down	168032500	285753333.3
Gamma-crystallin C	*Crygc*	1.99	Up	41397650	20802250
Gamma-crystallin D	*Crygd*	1.11	Up	153507500	138702500
Gamma-enolase	*Eno2*	1.02	Up	36167750000	35339750000
Gamma-glutamyltransferase 7;Gamma-glutamyltransferase 7 heavy chain;Gamma-glutamyltransferase 7 light chain	*Ggt7*	1.02	Down	129810000	131882500
Gamma-synuclein	*Sncg*	1.47	Down	609750000	896367500
Gelsolin	*Gsn*	1.11	Down	235106666.7	260227500
General transcription factor IIF subunit 2	*Gtf2f2*	1.06	Down	202722500	215075000
General transcription factor II-I	*Gtf2i*	1.04	Up	958157500	920080000
General vesicular transport factor p115	*Uso1*	1.11	Up	816972500	733087500
Gephyrin;Molybdopterin adenylyltransferase;Molybdopterin molybdenumtransferase	*Gphn*	1.00	Down	1436875000	1443850000
Geranylgeranyl transferase type-2 subunit alpha	*Rabggta*	1.08	Up	278692500	257482500
Glia maturation factor beta	*Gmfb*	1.30	Down	887127500	1156325000
Glial fibrillary acidic protein	*Gfap*	1.01	Down	45340500000	45946250000
Glucose-6-phosphate 1-dehydrogenase	*G6pdx*	1.04	Up	2812925000	2706425000
Glucose-6-phosphate isomerase	*Gpi*	1.01	Down	49207000000	49529000000
Glutamate decarboxylase 1	*Gad1*	1.00	Up	501082500	500717500
Glutamate decarboxylase 2	*Gad2*	1.06	Up	1393050000	1317050000
Glutamate dehydrogenase 1, mitochondrial	*Glud1*	1.10	Up	6985125000	6366775000
Glutamate receptor-interacting protein 1	*Grip1*	1.07	Up	130520000	121563333.3
Glutaminase kidney isoform, mitochondrial;Glutaminase kidney isoform 68 kDa chain;Glutaminase kidney isoform 65 kDa chain	*Gls*	1.08	Up	5973325000	5547725000
Glutamine synthetase	*Glul*	1.12	Up	75880750000	67674750000
Glutaredoxin-3	*Glrx3*	1.01	Up	532307500	525332500
Glutathione peroxidase 1	*Gpx1*	1.08	Down	319337500	346390000
Glutathione reductase	*Gsr*	1.11	Down	489040000	540580000
Glutathione S-transferase A6	*Gsta6*	1.14	Up	208200000	182676666.7
Glutathione S-transferase alpha-3;Glutathione S-transferase alpha-5	*Gsta3;Gsta5*	1.06	Down	31239000000	32991250000
Glutathione S-transferase alpha-4	*Gsta4*	1.08	Up	1383400000	1286475000

Glutathione S-transferase kappa 1	*Gstk1*	1.29	Up	184072500	142260000
Glutathione S-transferase Mu 1	*Gstm1*	1.09	Up	1912300000	1751625000
Glutathione S-transferase Mu 5	*Gstm5*	1.18	Up	2524200000	2143000000
Glutathione S-transferase omega-1	*Gsto1*	1.13	Up	688112500	608105000
Glutathione S-transferase P	*Gstp1*	1.00	Up	8590900000	8554050000
Glutathione S-transferase theta-2	*Gstt2*	1.01	Down	1782475000	1794100000
Glutathione S-transferase Yb-3	*Gstm3*	1.12	Down	9172700000	10287450000
Glutathione synthetase	*Gss*	1.04	Down	347905000	360752500
Glyceraldehyde-3-phosphate dehydrogenase	*Gapdh*	1.02	Up	1.85683E+11	1.82475E+11
Glycerol kinase	*Gk*	1.05	Down	228057500	240527500
Glycerophosphodiester phosphodiesterase 1	*Gde1*	1.20	Down	192427500	230490000
Glycerophosphodiester phosphodiesterase domain-containing protein 1	*Gdpd1*	1.12	Down	118494666.7	133080000
Glycine cleavage system H protein, mitochondrial	*Gcsh*	1.76	Up	142490000	80924000
Glycine--tRNA ligase	*Gars*	1.09	Down	1230860000	1342525000
Glycogen phosphorylase, brain form	*Pygb*	1.05	Up	9647900000	9177075000
Glycogen phosphorylase, liver form	*Pygl*	1.26	Up	326572500	260172500
Glycogen phosphorylase, muscle form	*Pygm*	1.19	Up	90068333.33	75990000
Glycogen synthase kinase-3 alpha	*Gsk3a*	1.02	Up	253237500	248042500
Glycogen synthase kinase-3 beta	*Gsk3b*	1.01	Up	467957500	461822500
Glycogenin-1	*Gyg1*	1.10	Down	407350000	447437500
Glycylpeptide N-tetradecanoyltransferase 1	*Nmt1*	1.11	Up	295325000	266047500
Glyoxalase domain-containing protein 4	*Glod4*	1.02	Up	1212450000	1188475000
GMP reductase 1	*Gmpr*	1.10	Down	199357500	219190000
GMP synthase [glutamine-hydrolyzing]	*Gmps*	1.07	Up	742080000	690467500
Golgi apparatus protein 1	*Glg1*	1.25	Up	522922500	419327500
Golgi reassembly-stacking protein 1	*Gorasp1*	1.47	Up	56870000	38790000
Golgi reassembly-stacking protein 2	*Gorasp2*	1.18	Down	297852500	352140000
Golgi resident protein GCP60	*Acbd3*	1.97	Up	117733500	59821500
Golgi SNAP receptor complex member 2	*Gosr2*	1.25	Down	118532500	147585250
Golgin subfamily A member 2	*Golga2*	1.26	Up	145723333.3	116085000
Golgin subfamily A member 4	*Golga4*	1.27	Down	52507000	66754000
GPI inositol-deacylase	*Pgap1*	1.74	Down	81943500	142834750
Graves disease carrier protein	*Slc25a16*	1.17	Up	8755850	7511566.667
GRIP1-associated protein 1	*Gripap1*	1.14	Down	210952500	240100000
Growth arrest-specific protein 7	*Gas7*	1.12	Up	31886000	28573000
Growth factor receptor-bound protein 2	*Grb2*	1.31	Down	313412500	409030000
Growth hormone-inducible transmembrane protein	*Ghitm*	1.29	Down	227832500	294775000
GrpE protein homolog 1, mitochondrial	*Grpel1*	1.09	Up	394355000	360757500
GTP:AMP phosphotransferase AK3, mitochondrial	*Ak3*	1.07	Down	266315000	285325000
GTPase HRas;GTPase HRas, N-terminally processed	*Hras*	1.07	Down	622460000	668580000
GTPase KRas;GTPase KRas, N-terminally processed	*Kras*	1.30	Down	245123333.3	317807500
GTP-binding nuclear protein Ran;GTP-binding nuclear protein Ran, testis-specific isoform	*Ran;Rasl2-9*	1.18	Down	9570850000	11275775000
GTP-binding protein 1	*Gtpbp1*	1.04	Down	484715000	503775000
GTP-binding protein Rheb	*Rheb*	1.17	Up	260770000	222916666.7
GTP-binding protein SAR1b	*Sar1b*	1.04	Down	165343333.3	172272500
Guanine nucleotide-binding protein G(i) subunit alpha-1	*Gnai1*	1.03	Down	672870000	694762500
Guanine nucleotide-binding protein G(i) subunit alpha-2	*Gnai2*	1.10	Up	6426075000	5866200000
Guanine nucleotide-binding protein G(I)/G(S)/G(O) subunit gamma-11	*Gng11*	2.00	Down	317790000	634080000

Guanine nucleotide-binding protein G(I)/G(S)/G(T) subunit beta-1	*Gnb1*	1.12	Up	80706250000	72144000000
Guanine nucleotide-binding protein G(I)/G(S)/G(T) subunit beta-2	*Gnb2*	1.03	Up	6113350000	5906675000
Guanine nucleotide-binding protein G(I)/G(S)/G(T) subunit beta-3	*Gnb3*	1.05	Up	5785950000	5497625000
Guanine nucleotide-binding protein G(k) subunit alpha	*Gnai3*	1.16	Down	212043333.3	245787500
Guanine nucleotide-binding protein G(o) subunit alpha	*Gnao1*	1.12	Up	32652500000	29154750000
Guanine nucleotide-binding protein G(q) subunit alpha	*Gnaq*	1.10	Up	1922075000	1743100000
Guanine nucleotide-binding protein G(s) subunit alpha isoforms short;Guanine nucleotide-binding protein G(s) subunit alpha isoforms XLas	*Gnas*	1.01	Down	592595000	596167500
Guanine nucleotide-binding protein G(t) subunit alpha-3	*Gnat3*	1.08	Up	8918625000	8286775000
Guanine nucleotide-binding protein G(z) subunit alpha	*Gnaz*	1.17	Up	1046917500	897355000
Guanine nucleotide-binding protein subunit alpha-11	*Gna11*	1.01	Up	492622500	487447500
Guanine nucleotide-binding protein subunit alpha-13	*Gna13*	1.04	Up	244402500	235722500
Guanine nucleotide-binding protein subunit beta-2-like 1;Guanine nucleotide-binding protein subunit beta-2-like 1, N-terminally processed	*Gnb2l1*	1.04	Up	4232350000	4068100000
Guanine nucleotide-binding protein subunit beta-5	*Gnb5*	1.25	Up	2115825000	1697950000
Guanine nucleotide-binding protein-like 1	*Gnl1*	1.12	Down	108306750	121226666.7
Guanylate cyclase soluble subunit alpha-2	*Gucy1a2*	1.34	Up	64621500	48102000
Guanylate cyclase soluble subunit alpha-3	*Gucy1a3*	1.02	Up	1631400000	1603750000
Guanylate cyclase soluble subunit beta-1	*Gucy1b3*	1.01	Down	3295875000	3315875000
Guanylyl cyclase GC-E	*Gucy2e*	1.19	Up	1921875000	1611225000
H(+)/Cl(-) exchange transporter 4	*Clcn4*	1.02	Down	45154000	45871000
H/ACA ribonucleoprotein complex subunit 4	*Dkc1*	1.11	Down	193385000	214427500
Haloacid dehalogenase-like hydrolase domain-containing protein 2	*Hdhd2*	1.07	Up	572632500	535422500
Haptoglobin;Haptoglobin alpha chain;Haptoglobin beta chain	*Hp*	1.35	Down	57383000	77297500
Heat shock 70 kDa protein 13	*Hspa13*	1.00	Down	188020000	188536666.7
Heat shock 70 kDa protein 1B;Heat shock 70 kDa protein 1A	*Hspa1a*	1.18	Up	8285150000	7034350000
Heat shock 70 kDa protein 4	*Hspa4*	1.00	Up	6784950000	6778875000
Heat shock cognate 71 kDa protein	*Hspa8*	1.13	Up	59393000000	52725000000
Heat shock protein 105 kDa	*Hsph1*	1.16	Up	951197500	818570000
Heat shock protein 75 kDa, mitochondrial	*Trap1*	1.08	Down	619815000	669042500
Heat shock protein HSP 90-alpha	*Hsp90aa1*	1.05	Up	53325000000	50677500000
Heat shock protein HSP 90-beta	*Hsp90ab1*	1.13	Up	15079000000	13377250000
Heat shock-related 70 kDa protein 2	*Hspa2*	1.25	Down	335715000	421197500
Heme oxygenase 2	*Hmox2*	1.07	Up	221450000	206350000
Hemoglobin subunit alpha-1/2	*Hba1*	1.13	Down	27838000000	31397500000
Hemoglobin subunit beta-1	*Hbb*	1.14	Down	39393750000	44857000000
Hemoglobin subunit beta-2	*Hbb-b2*	2.31	Down	1296967500	2997475000
Hemopexin	*Hpx*	1.06	Down	255630000	271825000
Hepatocyte growth factor-regulated tyrosine kinase substrate	*Hgs*	1.14	Down	286605000	326682500
Hepatoma-derived growth factor	*Hdgf*	1.12	Up	1721550000	1539525000
Hepatoma-derived growth factor-related protein 2	*Hdgfrp2*	1.13	Up	296447500	262925000
Hepatoma-derived growth factor-related protein 3	*Hdgfrp3*	1.05	Up	826805000	785612500

Heterochromatin protein 1-binding protein 3	*Hp1bp3*	1.33	Up	503252500	377260000
Heterogeneous nuclear ribonucleoprotein A1;Heterogeneous nuclear ribonucleoprotein A1, N-terminally processed	*Hnrnpa1*	1.18	Down	5288775000	6215050000
Heterogeneous nuclear ribonucleoprotein A3	*Hnrnpa3*	1.21	Down	9026475000	10925000000
Heterogeneous nuclear ribonucleoprotein C	*Hnrnpc*	1.16	Down	3123450000	3624950000
Heterogeneous nuclear ribonucleoprotein D0	*Hnrnpd*	1.00	Down	10995725000	11008500000
Heterogeneous nuclear ribonucleoprotein D-like	*Hnrnpdl*	1.17	Down	1690750000	1971825000
Heterogeneous nuclear ribonucleoprotein F;Heterogeneous nuclear ribonucleoprotein F, N-terminally processed	*Hnrnpf*	1.20	Down	1682732500	2024550000
Heterogeneous nuclear ribonucleoprotein H;Heterogeneous nuclear ribonucleoprotein H, N-terminally processed	*Hnrnph1*	1.13	Down	7179325000	8088400000
Heterogeneous nuclear ribonucleoprotein H2	*Hnrnph2*	1.03	Down	2278400000	2346125000
Heterogeneous nuclear ribonucleoprotein K	*Hnrnpk*	1.01	Up	20011500000	19891250000
Heterogeneous nuclear ribonucleoprotein M	*Hnrnpm*	1.01	Down	7175225000	7281125000
Heterogeneous nuclear ribonucleoprotein Q	*Syncrip*	1.05	Up	1231685000	1171525000
Heterogeneous nuclear ribonucleoproteins A2/B1	*Hnrnpa2b1*	1.33	Down	16343250000	21767250000
Hexokinase-1	*Hk1*	1.08	Up	21469250000	19893000000
Hexokinase-2	*Hk2*	1.06	Up	5568225000	5244700000
High affinity choline transporter 1	*Slc5a7*	1.02	Down	52821500	53963000
High mobility group nucleosome-binding domain-containing protein 5	*Hmgn5*	1.04	Down	350206666.7	363690000
High mobility group protein B1	*Hmgb1*	1.08	Down	18432250000	19818000000
High mobility group protein B2	*Hmgb2*	1.19	Down	3591000000	4291200000
High mobility group protein HMG-I/HMG-Y	*Hmga1*	1.06	Up	149619666.7	141102500
Hippocalcin-like protein 1	*Hpcal1*	1.07	Up	1553875000	1446350000
Hippocalcin-like protein 4	*Hpcal4*	1.22	Up	361112500	296485000
Histidine triad nucleotide-binding protein 1	*Hint1*	1.09	Down	3708975000	4035175000
Histone deacetylase 1	*Hdac1*	1.14	Down	790920000	899390000
Histone H1.4	*Hist1h1e*	1.08	Up	2014800000	1872325000
Histone H2A type 2-A	*Hist2h2aa3*	1.99	Down	4218075000	8381700000
Histone H2A.Z	*H2afz*	1.07	Up	1872875000	1748800000
Histone H2B type 1;Histone H2B type 1-A	*Hist1h2ba*	1.78	Down	4303400000	7642825000
Histone H3.3;Histone H3.1	*H3f3b*	1.87	Down	925393333.3	1734025000
Histone H4;Osteogenic growth peptide	*Hist1h4b*	2.07	Down	2266950000	4700975000
Histone-binding protein RBBP7	*Rbbp7*	1.08	Up	1297550000	1200075000
Homer protein homolog 1	*Homer1*	1.40	Down	26482000	37039000
Hsc70-interacting protein	*St13*	1.22	Down	1507625000	1832875000
Hsp90 co-chaperone Cdc37;Hsp90 co-chaperone Cdc37, N-terminally processed	*Cdc37*	1.06	Up	800742500	756665000
Huntingtin	*Htt*	1.16	Down	133722500	155640000
Hydroxyacyl-coenzyme A dehydrogenase, mitochondrial	*Hadh*	1.15	Up	410652500	357877500
Hydroxyacylglutathione hydrolase, mitochondrial	*Hagh*	1.19	Up	719777500	605027500
Hydroxymethylglutaryl-CoA lyase, mitochondrial	*Hmgcl*	1.16	Up	547810000	473795000

Hydroxymethylglutaryl-CoA synthase, cytoplasmic	*Hmgcs1*	1.27	Down	165335000	209472500
Hydroxysteroid dehydrogenase-like protein 2	*Hsdl2*	1.01	Up	190190000	187676666.7
Hypoxanthine-guanine phosphoribosyltransferase	*Hprt1*	1.06	Up	12138750000	11473150000
Hypoxia up-regulated protein 1	*Hyou1*	1.06	Up	2153325000	2036350000
Ig gamma-2A chain C region	*Igg-2a*	1.06	Down	371045000	394817500
Ig gamma-2B chain C region	*Igh-1a*	1.05	Down	198845000	208820000
Ig kappa chain C region, A allele	*N/A*	1.14	Down	202202500	230445000
Importin subunit alpha-5;Importin subunit alpha-5, N-terminally processed;Importin subunit alpha-6	*Kpna1;Kpna5*	1.01	Up	208213333.3	206620000
Importin subunit beta-1	*Kpnb1*	1.04	Up	3629150000	3477400000
Inactive hydroxysteroid dehydrogenase-like protein 1	*Hsdl1*	1.21	Up	160574750	132510000
Inactive phospholipase C-like protein 1	*Plcl1*	1.02	Up	151170000	147830000
Inosine triphosphate pyrophosphatase	*Itpa*	1.14	Down	654085000	748060000
Inosine-5-monophosphate dehydrogenase 1	*Impdh1*	1.15	Up	8673825000	7574475000
Inosine-5-monophosphate dehydrogenase 2	*Impdh2*	1.01	Up	615880000	608345000
Inositol hexakisphosphate and diphosphoinositol-pentakisphosphate kinase 1	*Ppip5k1*	1.34	Down	51645000	69024000
Inositol monophosphatase 1	*Impa1*	1.01	Up	3246100000	3211875000
Inositol polyphosphate 5-phosphatase OCRL-1	*Ocrl*	1.21	Down	57134500	68954000
Inositol-3-phosphate synthase 1	*Isyna1*	1.03	Up	99229000	96346000
Insulin-degrading enzyme	*Ide*	1.01	Down	337337500	339600000
Integral membrane protein 2B;BRI2, membrane form;BRI2 intracellular domain;BRI2C, soluble form;Bri23 peptide	*Itm2b*	1.40	Down	71998000	101122500
Integral membrane protein 2C;CT-BRI3	*Itm2c*	1.27	Up	124291333.3	98063000
Integrin beta-1	*Itgb1*	1.16	Up	341730000	295642500
Integrin-linked kinase-associated serine/threonine phosphatase 2C	*Ilkap*	1.08	Up	233235000	215125000
Integrin-linked protein kinase	*Ilk*	1.18	Down	78921500	92857750
Inter-alpha-trypsin inhibitor heavy chain H3	*Itih3*	1.10	Down	42086500	46236000
Interferon regulatory factor 2-binding protein-like	*Irf2bpl*	1.08	Down	209100000	225486666.7
Interferon-inducible double-stranded RNA-dependent protein kinase activator A	*Prkra*	1.13	Down	317282500	359360000
Interleukin enhancer-binding factor 2	*Ilf2*	1.05	Up	2212200000	2113300000
Interleukin enhancer-binding factor 3	*Ilf3*	1.06	Up	2496450000	2353675000
Interphotoreceptor matrix proteoglycan 1	*Impg1*	1.25	Up	1269950000	1015350000
Interphotoreceptor matrix proteoglycan 2	*Impg2*	1.33	Up	1663300000	1255125000
Intersectin-1	*Itsn1*	1.17	Up	259090000	220515000
IQ motif and SEC7 domain-containing protein 3	*Iqsec3*	1.00	Down	432840000	433207500
Isoamyl acetate-hydrolyzing esterase 1 homolog	*Iah1*	1.14	Down	423542500	482540000
Isoaspartyl peptidase/L-asparaginase;Isoaspartyl peptidase/L-asparaginase alpha chain;Isoaspartyl peptidase/L-asparaginase beta chain	*Asrgl1*	1.10	Up	998547500	907345000
Isochorismatase domain-containing protein 1	*Isoc1*	1.11	Up	436585000	394977500
Isocitrate dehydrogenase [NAD] subunit alpha, mitochondrial	*Idh3a*	1.07	Up	9318050000	8738100000
Isocitrate dehydrogenase [NAD] subunit beta, mitochondrial	*Idh3B*	1.03	Up	5476250000	5292225000

Isocitrate dehydrogenase [NAD] subunit gamma 1, mitochondrial	*Idh3g*	1.00	Down	2963100000	2970175000
Isocitrate dehydrogenase [NADP] cytoplasmic	*Idh1*	1.00	Up	2210150000	2202825000
Isocitrate dehydrogenase [NADP], mitochondrial	*Idh2*	1.09	Up	2271350000	2085850000
Isovaleryl-CoA dehydrogenase, mitochondrial	*Ivd*	1.19	Down	1001740000	1191650000
IST1 homolog	*Ist1*	1.17	Up	233097500	198567500
Jouberin	*Ahi1*	1.19	Down	127176666.7	151715000
Junction plakoglobin	*Jup*	1.10	Up	84295000	76313750
Kelch-like protein 22	*Klhl22*	1.40	Down	26188000	36730000
Keratin, type I cytoskeletal 18	*Krt18*	1.33	Up	212460000	159785000
Keratin, type II cytoskeletal 8	*Krt8*	1.08	Down	153700000	166310000
Ketimine reductase mu-crystallin	*Crym*	1.13	Up	24126750000	21444750000
KH domain-containing, RNA-binding, signal transduction-associated protein 1	*Khdrbs1*	1.30	Down	1402775000	1824300000
KH domain-containing, RNA-binding, signal transduction-associated protein 3	*Khdrbs3*	1.07	Down	215170000	229326666.7
KIF1-binding protein	*Kbp*	1.09	Down	80833000	88035000
Kinase D-interacting substrate of 220 kDa	*Kidins220*	1.16	Up	17879500	15479000
Kinesin heavy chain isoform 5A	*Kif5a*	1.01	Down	220686666.7	223190000
Kinesin light chain 1	*Klc1*	1.01	Up	556360000	548912500
Kinesin-1 heavy chain	*Kif5b*	1.03	Down	1391275000	1434400000
Kinesin-like protein KIF1B	*Kif1b*	1.03	Down	314005000	324820000
Kinesin-like protein KIF2A	*Kif2a*	1.02	Up	647130000	632467500
Kinesin-like protein KIF3C	*Kif3c*	1.30	Up	114440000	87719000
Kynurenine/alpha-aminoadipate aminotransferase, mitochondrial	*Aadat*	1.18	Up	544120000	462027500
Kynurenine--oxoglutarate transaminase 1, mitochondrial	*Ccbl1*	1.01	Down	206425000	208560000
Lactadherin	*Mfge8*	1.07	Down	272680000	290785000
Lactoylglutathione lyase	*Glo1*	1.01	Down	8229225000	8296350000
Lambda-crystallin homolog	*Cryl1*	1.07	Down	152153333.3	162827500
Lamina-associated polypeptide 2, isoform beta	*Tmpo*	1.40	Down	1405150000	1960950000
Lamin-B1	*Lmnb1*	3.42	Down	1433960000	4896975000
LanC-like protein 1	*Lancl1*	1.03	Down	2034250000	2104425000
La-related protein 7	*Larp7*	1.24	Down	90351000	112020000
Large neutral amino acids transporter small subunit 1	*Slc7a5*	5.14	Up	132143000	25704000
Large proline-rich protein BAG6	*Bag6*	1.12	Up	665765000	592242500
Latrophilin-3	*Lphn3*	1.08	Up	171890000	158825000
LDLR chaperone MESD	*Mesdc2*	1.02	Up	119337333.3	116795000
Lethal(2) giant larvae protein homolog 1	*Llgl1*	1.20	Up	171165000	142760000
Leucine carboxyl methyltransferase 1	*Lcmt1*	1.02	Up	170415000	166320000
Leucine zipper transcription factor-like protein 1	*Lztfl1*	1.10	Up	294555000	267435000
Leucine-rich PPR motif-containing protein, mitochondrial	*Lrpprc*	1.10	Up	2255325000	2048925000
Leucine-rich repeat-containing protein 15	*Lrrc15*	1.14	Up	141670000	124190000
Leucine-rich repeat-containing protein 4B	*Lrrc4b*	1.17	Down	85375500	99688500
Leucine-rich repeat-containing protein 57	*Lrrc57*	1.12	Down	133135000	149115000
Leucine-rich repeat-containing protein 59	*Lrrc59*	1.26	Up	1357850000	1077587500
Leukocyte surface antigen CD47	*Cd47*	1.00	Up	400025000	398810000
Leukotriene A-4 hydrolase	*Lta4h*	1.04	Up	3109375000	2988400000
LIM and SH3 domain protein 1	*Lasp1*	1.46	Down	931942500	1362250000
Limbic system-associated membrane protein	*Lsamp*	1.09	Down	1787800000	1956500000
Lipid phosphate phosphohydrolase 3	*Ppap2b*	1.31	Up	121150000	92748000
Lipoma HMGIC fusion partner-like protein 4	*Lhfpl4*	1.05	Up	34271500	32633250
Liprin-alpha-3	*Ppfia3*	1.50	Up	132940000	88788000

L-lactate dehydrogenase A chain	*Ldha*	1.01	Down	1.30363E+11	1.31398E+11
L-lactate dehydrogenase B chain	*Ldhb*	1.08	Down	16327500000	17644500000
Lon protease homolog, mitochondrial	*Lonp1*	1.02	Down	837132500	853117500
Long-chain fatty acid transport protein 1	*Slc27a1*	1.00	Down	1101237500	1104200000
Long-chain specific acyl-CoA dehydrogenase, mitochondrial	*Acadl*	1.10	Up	115816333.3	105346333.3
Long-chain-fatty-acid--CoA ligase 1	*Acsl1*	1.07	Down	1378400000	1473200000
Long-chain-fatty-acid--CoA ligase 3	*Acsl3*	1.15	Down	811712500	934005000
Long-chain-fatty-acid--CoA ligase 6	*Acsl6*	1.02	Up	5044075000	4948350000
Low molecular weight phosphotyrosine protein phosphatase	*Acp1*	1.08	Down	837690000	904312500
Lupus La protein homolog	*Ssb*	1.06	Up	2068575000	1944650000
Lysophosphatidylcholine acyltransferase 1	*Lpcat1*	1.05	Down	511292500	534890000
Lysosomal alpha-glucosidase	*Gaa*	1.35	Down	77176000	104137333.3
Lysosome membrane protein 2	*Scarb2*	1.48	Down	120363666.7	177835000
Lysosome-associated membrane glycoprotein 1	*Lamp1*	1.19	Down	212067500	253380000
Lysosome-associated membrane glycoprotein 5	*Lamp5*	1.49	Up	129775000	87132000
m7GpppX diphosphatase	*Dcps*	1.08	Up	1506775000	1397800000
Macrophage migration inhibitory factor	*Mif*	1.03	Down	6875150000	7047475000
MAGUK p55 subfamily member 4	*Mpp4*	1.14	Down	434482500	495912500
MAGUK p55 subfamily member 7	*Mpp7*	1.20	Down	56831000	68116000
Major prion protein	*Prnp*	1.01	Up	685240000	675465000
Malate dehydrogenase, cytoplasmic	*Mdh1*	1.10	Up	35442750000	32366500000
Malate dehydrogenase, mitochondrial	*Mdh2*	1.08	Up	40608750000	37482000000
Malectin	*Mlec*	1.05	Up	180687500	172533333.3
Maleylacetoacetate isomerase	*Gstz1*	1.10	Down	96771000	106100666.7
Malignant T-cell-amplified sequence 1	*Mcts1*	1.08	Up	165933333.3	152983333.3
Mammalian ependymin-related protein 1	*Epdr1*	1.34	Down	77927000	104124666.7
Mannose-6-phosphate isomerase	*Mpi*	1.11	Up	1217700000	1101542500
Mannosyl-oligosaccharide glucosidase	*Mogs*	1.16	Down	113371000	131737500
MAP kinase-activating death domain protein	*Madd*	1.01	Up	648107500	642735000
MAP/microtubule affinity-regulating kinase 3	*Mark3*	1.04	Down	93509000	96933750
Matrin-3	*Matr3*	1.21	Down	7945775000	9591100000
Medium-chain specific acyl-CoA dehydrogenase, mitochondrial	*Acadm*	1.02	Up	1112397500	1091085000
Medium-wave-sensitive opsin 1	*Opn1mw*	1.20	Up	208880000	174285000
Membrane-associated guanylate kinase, WW and PDZ domain-containing protein 1	*Magi1*	1.05	Up	60856666.67	58208000
Membrane-associated guanylate kinase, WW and PDZ domain-containing protein 3	*Magi3*	1.07	Up	64627500	60290666.67
Membrane-associated phosphatidylinositol transfer protein 1	*Pitpnm1*	1.03	Up	426175000	412740000
Membrane-associated progesterone receptor component 1	*Pgrmc1*	1.01	Down	1613775000	1628825000
Membrane-associated progesterone receptor component 2	*Pgrmc2*	1.17	Up	431296666.7	370033333.3
Mesencephalic astrocyte-derived neurotrophic factor	*Manf*	1.16	Down	222286666.7	257200000
Metabotropic glutamate receptor 2	*Grm2*	1.03	Up	217622500	212275000
Metastasis-associated protein MTA1	*Mta1*	1.04	Up	132120000	127306666.7
Methionine adenosyltransferase 2 subunit beta	*Mat2b*	1.24	Up	431447500	347255000
Methionine aminopeptidase 2	*Metap2*	1.50	Down	152010000	228252500
Methyl-CpG-binding protein 2	*Mecp2*	1.84	Down	217625000	399940000
Methylcrotonoyl-CoA carboxylase subunit alpha, mitochondrial	*Mccc1*	1.01	Down	109674250	111313333.3
Methylmalonate-semialdehyde dehydrogenase [acylating], mitochondrial	*Aldh6a1*	1.02	Up	503292500	494407500

Methylosome protein 50	*Wdr77*	1.05	Down	264726666.7	279057500
Methylthioribose-1-phosphate isomerase	*Mri1*	1.20	Up	416187500	346792500
Mevalonate kinase	*Mvk*	1.17	Down	43639500	51040666.67
MICOS complex subunit Mic25	*Chchd6*	1.14	Down	374015000	424927500
MICOS complex subunit Mic60	*Immt*	1.00	Down	2662175000	2668200000
Microsomal glutathione S-transferase 1	*Mgst1*	1.13	Down	95067000	107685000
Microtubule-actin cross-linking factor 1	*Macf1*	1.01	Down	937972500	947847500
Microtubule-associated protein 1A;MAP1A heavy chain;MAP1 light chain LC2	*Map1a*	1.01	Down	2047050000	2073925000
Microtubule-associated protein 1B;MAP1B heavy chain;MAP1 light chain LC1	*Map1b*	1.01	Up	12824500000	12741250000
Microtubule-associated protein 1S;MAP1S heavy chain;MAP1S light chain	*Map1s*	1.04	Up	428992500	412165000
Microtubule-associated protein 2	*Map2*	1.29	Down	4308975000	5560100000
Microtubule-associated protein 4	*Map4*	1.24	Down	2913675000	3619925000
Microtubule-associated protein 6	*Map6*	1.14	Down	3230575000	3688300000
Microtubule-associated protein RP/EB family member 1	*Mapre1*	1.03	Down	569165000	585057500
Microtubule-associated protein RP/EB family member 2	*Mapre2*	1.44	Down	682330000	980785000
Microtubule-associated protein RP/EB family member 3	*Mapre3*	1.06	Down	2777875000	2941950000
Microtubule-associated protein tau	*Mapt*	1.19	Down	2321250000	2772675000
Microtubule-associated proteins 1A/1B light chain 3A	*Map1lc3a*	1.02	Up	1513850000	1480325000
Microtubule-associated proteins 1A/1B light chain 3B	*Map1lc3b*	1.07	Down	669770000	718927500
Misshapen-like kinase 1	*Mink1*	1.17	Down	97424500	113510000
Mitochondrial 2-oxoglutarate/malate carrier protein	*Slc25a11*	1.27	Up	1342925000	1055355000
Mitochondrial amidoxime reducing component 2	*Marc--2*	1.11	Down	113613333.3	126490000
Mitochondrial antiviral-signaling protein	*Mavs*	1.38	Down	84098000	115773333.3
Mitochondrial carnitine/acylcarnitine carrier protein	*Slc25a20*	1.37	Up	238892500	174215000
Mitochondrial fission 1 protein	*Fis1*	1.23	Up	951905000	771585000
Mitochondrial glutamate carrier 2	*Slc25a18*	1.05	Up	1033696667	980795000
Mitochondrial import inner membrane translocase subunit Tim13	*Timm13*	1.33	Up	183543333.3	138323333.3
Mitochondrial import inner membrane translocase subunit TIM44	*Timm44*	1.16	Up	223146666.7	192712500
Mitochondrial import inner membrane translocase subunit Tim9	*Timm9*	1.22	Down	171650000	209560000
Mitochondrial import receptor subunit TOM22 homolog	*Tomm22*	1.02	Up	467410000	456870000
Mitochondrial import receptor subunit TOM34	*Tomm34*	1.06	Down	81306666.67	86036333.33
Mitochondrial import receptor subunit TOM40 homolog	*Tomm40*	1.46	Up	297047500	202850000
Mitochondrial import receptor subunit TOM70	*Tomm70a*	1.04	Down	2242750000	2325525000
Mitochondrial peptide methionine sulfoxide reductase	*Msra*	1.18	Up	254613333.3	215870000
Mitochondrial pyruvate carrier 1	*Mpc1*	2.28	Up	796500000	348980000
Mitochondrial pyruvate carrier 2	*Mpc2*	1.02	Down	230847500	234632500
Mitochondrial Rho GTPase 2	*Rhot2*	1.15	Up	1319100000	1143925000
Mitofusin-2	*Mfn2*	1.13	Up	190307500	168152500
Mitogen-activated protein kinase 1	*Mapk1*	1.01	Up	4056425000	3998650000
Mitogen-activated protein kinase 3	*Mapk3*	1.00	Up	1296077500	1292500000
Mitogen-activated protein kinase 9;Mitogen-activated protein kinase 8	*Mapk9;Mapk8*	1.14	Down	192187500	218172500
MOB-like protein phocein	*Mob4*	1.02	Up	109010000	107277666.7
Moesin	*Msn*	1.02	Down	3197925000	3259375000

Monoacylglycerol lipase ABHD12	*Abhd12*	1.00	Up	541587500	540372500
Monoacylglycerol lipase ABHD6	*Abhd6*	1.31	Up	190540000	145712500
Monocarboxylate transporter 1	*Slc16a1*	1.46	Up	930032500	635025000
Monoglyceride lipase	*Mgll*	1.05	Up	1162837500	1106025000
mRNA cap guanine-N7 methyltransferase	*Rnmt*	1.12	Up	637070000	566705000
Multidrug resistance protein 1;Multidrug resistance protein 3	*Abcb1;Abcb4*	1.03	Down	45812000	47024000
Multifunctional protein ADE2;Phosphoribosylaminoimidazole-succinocarboxamide synthase;Phosphoribosylaminoimidazole carboxylase	*Paics*	1.12	Up	632227500	566955000
Murinoglobulin-1	*Mug1*	1.26	Up	101826000	80973000
Muscleblind-like protein 2	*Mbnl2*	1.05	Up	225252500	213955000
Myb-binding protein 1A	*Mybbp1a*	1.07	Up	257660000	241562500
Myc box-dependent-interacting protein 1	*Bin1*	1.07	Up	2433775000	2279275000
Mycophenolic acid acyl-glucuronide esterase, mitochondrial	*Abhd10*	1.04	Down	601600000	627205000
Myocyte-specific enhancer factor 2D	*Mef2d*	1.16	Down	144192500	167382500
Myosin light polypeptide 6	*Myl6*	1.14	Down	537167500	611620000
Myosin regulatory light chain 12B;Myosin regulatory light chain RLC-A;Myosin regulatory light polypeptide 9	*Myl12b;Rlc-a;Myl9*	1.14	Down	269240000	308247500
Myosin-10	*Myh10*	1.02	Up	968467500	949935000
Myosin-9	*Myh9*	1.06	Up	334707500	316377500
Myotrophin	*Mtpn*	1.02	Down	503385000	512265000
Myristoylated alanine-rich C-kinase substrate	*Marcks*	2.47	Up	1760595000	713300000
N(G),N(G)-dimethylarginine dimethylaminohydrolase 1	*Ddah1*	1.09	Up	1622050000	1481400000
N(G),N(G)-dimethylarginine dimethylaminohydrolase 2	*Ddah2*	1.08	Up	5913325000	5498725000
Na(+)/H(+) exchange regulatory cofactor NHE-RF1	*Slc9a3r1*	1.00	Up	458587500	457092500
N-acetyl-D-glucosamine kinase	*Nagk*	1.20	Down	48486500	58356333.33
N-acetylgalactosamine kinase	*Galk2*	1.01	Down	98739000	99937000
N-acylneuraminate cytidylyltransferase	*Cmas*	1.14	Down	515305000	589610000
NAD kinase 2, mitochondrial	*Nadk2*	1.04	Down	385832500	402717500
NAD(P)H dehydrogenase [quinone] 1	*Nqo1*	1.48	Down	53623500	79525500
NAD(P)H-hydrate epimerase	*Apoa1bp*	1.02	Down	855900000	872240000
NAD-dependent protein deacetylase sirtuin-2	*Sirt2*	1.01	Down	915137500	922140000
NAD-dependent protein deacylase sirtuin-5, mitochondrial	*Sirt5*	1.88	Up	172790000	91968666.67
NADH dehydrogenase [ubiquinone] 1 alpha subcomplex subunit 10, mitochondrial	*Ndufa10*	1.11	Down	3027750000	3373000000
NADH dehydrogenase [ubiquinone] 1 alpha subcomplex subunit 11	*Ndufa11*	1.42	Down	378237500	537390000
NADH dehydrogenase [ubiquinone] 1 alpha subcomplex subunit 5	*Ndufa5*	1.26	Up	802457500	638547500
NADH dehydrogenase [ubiquinone] 1 alpha subcomplex subunit 9, mitochondrial	*Ndufa9*	1.03	Down	2073675000	2133925000
NADH dehydrogenase [ubiquinone] flavoprotein 2, mitochondrial	*Ndufv2*	1.05	Up	876885000	832072500
NADH dehydrogenase [ubiquinone] iron-sulfur protein 2, mitochondrial	*Ndufs2*	1.00	Down	3406550000	3416850000
NADH dehydrogenase [ubiquinone] iron-sulfur protein 4, mitochondrial	*Ndufs4*	1.03	Up	710217500	688227500
NADH dehydrogenase [ubiquinone] iron-sulfur protein 6, mitochondrial	*Ndufs6*	1.39	Down	588817500	815757500
NADH-cytochrome b5 reductase 1	*Cyb5r1*	1.02	Up	919035000	902275000

NADH-cytochrome b5 reductase 3;NADH-cytochrome b5 reductase 3 membrane-bound form;NADH-cytochrome b5 reductase 3 soluble form	*Cyb5r3*	1.04	Up	532020000	512635000
NADH-ubiquinone oxidoreductase 75 kDa subunit, mitochondrial	*Ndufs1*	1.00	Down	8101600000	8134650000
NADH-ubiquinone oxidoreductase chain 1	*Mtnd1*	1.05	Up	298100000	284315000
NADH-ubiquinone oxidoreductase chain 4	*Mtnd4*	1.08	Down	525252500	569520000
NADH-ubiquinone oxidoreductase chain 5	*Mtnd5*	1.01	Up	427002500	424805000
NADPH--cytochrome P450 reductase	*Por*	1.03	Up	1084940000	1053685000
N-alpha-acetyltransferase 25, NatB auxiliary subunit	*Naa25*	1.25	Down	42191000	52694000
Nardilysin	*Nrd1*	1.02	Down	310255000	316890000
Nck-associated protein 1	*Nckap1*	1.07	Up	1163925000	1084960000
NEDD8	*Nedd8*	1.11	Up	226593333.3	204710000
NEDD8-activating enzyme E1 catalytic subunit	*Uba3*	1.08	Up	594137500	550572500
NEDD8-activating enzyme E1 regulatory subunit	*Nae1*	1.00	Up	467112500	466367500
Neogenin	*Neo1*	1.48	Up	127860000	86328333.33
Neurabin-2	*Ppp1r9b*	1.13	Down	80872000	91412333.33
Neural cell adhesion molecule 1	*Ncam1*	1.05	Up	22134250000	21107500000
Neural cell adhesion molecule L1	*L1cam*	1.09	Down	275512500	300572500
Neural Wiskott-Aldrich syndrome protein	*Wasl*	1.14	Up	77079000	67454333.33
Neurexin-2	*Nrxn2*	1.44	Up	30077000	20940000
Neurexin-3	*Nrxn3*	1.02	Up	736855000	720675000
Neurocalcin-delta	*Ncald*	1.12	Down	254200000	285667500
Neurochondrin	*Ncdn*	1.02	Down	875472500	891115000
Neurofascin	*Nfasc*	1.01	Down	1757875000	1775750000
Neurofibromin	*Nf1*	1.47	Down	68270666.67	100527333.3
Neurofilament light polypeptide	*Nefl*	1.98	Down	545370000	1078150000
Neurofilament medium polypeptide	*Nefm*	1.20	Down	136570000	163363333.3
Neuroligin-2	*Nlgn2*	1.03	Down	245640000	252930000
Neuroligin-3	*Nlgn3*	1.04	Up	275516666.7	263877500
Neurolysin, mitochondrial	*Nln*	1.44	Up	94494000	65501000
Neuronal calcium sensor 1	*Ncs1*	1.79	Down	42340000	75598000
Neuronal cell adhesion molecule	*Nrcam*	1.12	Up	745157500	666162500
Neuronal growth regulator 1	*Negr1*	1.10	Up	1082662500	982225000
Neuronal membrane glycoprotein M6-a	*Gpm6a*	1.24	Up	3860850000	3113300000
Neuronal membrane glycoprotein M6-b	*Gpm6b*	1.21	Up	1238232500	1023492500
Neuronal migration protein doublecortin	*Dcx*	1.09	Down	185740000	202645000
Neuronal-specific septin-3	*Sep--3*	1.08	Down	848425000	913747500
Neuron-specific calcium-binding protein hippocalcin	*Hpca*	1.04	Up	537697500	516410000
Neuroplastin	*Nptn*	1.02	Down	2485100000	2536950000
Neurotrimin	*Ntm*	1.06	Down	1164640000	1234250000
Neutral cholesterol ester hydrolase 1	*Nceh1*	1.04	Up	522795000	500587500
NHP2-like protein 1;NHP2-like protein 1, N-terminally processed	*Nhp2l1*	1.15	Down	541045000	623755000
Niban-like protein 1	*Fam129b*	1.25	Up	137886666.7	110150000
Nicalin	*Ncln*	1.15	Down	148613333.3	171452500
Nicotinamide phosphoribosyltransferase	*Nampt*	1.01	Down	353320000	355360000
Ninjurin-1	*Ninj1*	1.11	Up	12808300	11497600
Nischarin	*Nisch*	1.03	Up	206256666.7	201090000
NLR family member X1	*Nlrx1*	1.13	Up	148370000	131865000
NmrA-like family domain-containing protein 1	*Nmral1*	1.13	Down	379520000	427590000
Non-histone chromosomal protein HMG-17	*Hmgn2*	1.51	Down	316087500	476235000
Non-lysosomal glucosylceramidase	*Gba2*	1.09	Up	104305000	96024333.33
Non-muscle caldesmon	*Cald1*	1.25	Down	86457500	108425500
Non-POU domain-containing octamer-binding protein	*Nono*	1.04	Down	2892200000	3000775000

Non-specific lipid-transfer protein	*Scp2*	1.03	Down	749987500	773745000
NSFL1 cofactor p47	*Nsfl1c*	1.08	Up	1115677500	1035252500
N-terminal EF-hand calcium-binding protein 1	*Necab1*	1.23	Down	48583000	59832666.67
Nuclear cap-binding protein subunit 1	*Ncbp1*	1.01	Up	644337500	637490000
Nuclear migration protein nudC	*Nudc*	1.18	Down	878617500	1039625000
Nuclear pore complex protein Nup155	*Nup155*	1.20	Down	206326666.7	247440000
Nuclear pore complex protein Nup54	*Nup54*	1.25	Down	89275500	111365000
Nuclear pore complex protein Nup93	*Nup93*	1.42	Down	141062500	200740000
Nuclear transport factor 2	*Nutf2*	1.07	Up	772705000	720110000
Nucleobindin-1	*Nucb1*	1.13	Down	129796666.7	146867500
Nucleolar and coiled-body phosphoprotein 1	*Nolc1*	1.22	Down	34645333.33	42272333.33
Nucleolar protein 58	*Nop58*	1.01	Down	289800000	292820000
Nucleolar RNA helicase 2	*Ddx21*	1.44	Up	184882500	128545000
Nucleolin	*Ncl*	1.08	Up	6253225000	5775675000
Nucleophosmin	*Npm1*	1.06	Up	3048100000	2889125000
Nucleoporin NUP53	*Nup35*	1.84	Down	61615000	113388666.7
Nucleoprotein TPR	*Tpr*	1.54	Down	393040000	606462500
Nucleoside diphosphate kinase A	*Nme1*	1.01	Down	7847075000	7895375000
Nucleoside diphosphate kinase B	*Nme2*	1.04	Up	1252555000	1200225000
Nucleosome assembly protein 1-like 1	*Nap1l1*	1.10	Up	1020265000	929487500
Nucleosome assembly protein 1-like 4	*Nap1l4*	1.02	Down	746545000	762087500
NudC domain-containing protein 2	*Nudcd2*	2.07	Up	85053333.33	41075000
O-acetyl-ADP-ribose deacetylase MACROD1	*Macrod1*	1.15	Up	245497500	213795000
Obg-like ATPase 1	*Ola1*	1.14	Up	1292010000	1137985000
OCIA domain-containing protein 1	*Ociad1*	1.08	Down	242795000	261737500
Omega-amidase NIT2	*Nit2*	1.09	Down	549477500	598920000
Opioid growth factor receptor	*Ogfr*	1.32	Down	39192000	51731000
Optineurin	*Optn*	1.21	Down	57419000	69396333.33
Ornithine aminotransferase, mitochondrial	*Oat*	1.01	Up	2432300000	2409175000
Osteoclast-stimulating factor 1	*Ostf1*	1.13	Down	34530500	38914000
OX-2 membrane glycoprotein	*Cd200*	1.04	Down	497602500	516742500
Oxidation resistance protein 1	*Oxr1*	1.10	Up	1351450000	1229575000
Oxygen-dependent coproporphyrinogen-III oxidase, mitochondrial	*Cpox*	1.05	Up	179945000	171033333.3
Oxysterol-binding protein-related protein 1	*Osbpl1a*	1.29	Up	196576666.7	152165000
Palmitoyl-protein thioesterase 1	*Ppt1*	1.05	Up	1171407500	1112425000
Pantothenate kinase 4	*Pank4*	1.16	Up	223960000	193767500
Parafibromin	*Cdc73*	1.23	Down	84347000	104102000
Paralemmin-1	*Palm*	1.20	Down	508286666.7	609970000
Paraspeckle component 1	*Pspc1*	1.02	Up	1464525000	1441975000
Parvalbumin alpha	*Pvalb*	1.26	Down	951177500	1196862500
Paxillin	*Pxn*	1.08	Up	25879000	23907000
PC4 and SFRS1-interacting protein	*Psip1*	1.07	Down	1739625000	1854550000
PDZ and LIM domain protein 5	*Pdlim5*	1.13	Down	275865000	311052500
PDZ domain-containing protein GIPC1	*Gipc1*	1.09	Down	56194000	61357666.67
Peflin	*Pef1*	1.10	Up	244060000	222200000
Peptidyl-prolyl cis-trans isomerase A;Peptidyl-prolyl cis-trans isomerase A, N-terminally processed	*Ppia*	1.04	Up	39240500000	37766250000
Peptidyl-prolyl cis-trans isomerase B	*Ppib*	1.02	Down	2237875000	2274025000
Peptidyl-prolyl cis-trans isomerase D	*Ppid*	1.01	Up	2376800000	2349775000
Peptidyl-prolyl cis-trans isomerase F, mitochondrial	*Ppif*	1.68	Down	49242000	82583000
Peptidyl-prolyl cis-trans isomerase FKBP1A	*Fkbp1a*	1.15	Up	889387500	771187500
Peptidyl-prolyl cis-trans isomerase FKBP4;Peptidyl-prolyl cis-trans isomerase FKBP4, N-terminally processed	*Fkbp4*	1.03	Down	2177175000	2239400000
Peptidyl-prolyl cis-trans isomerase FKBP8	*Fkbp8*	1.43	Up	366880000	255950000

Peripheral plasma membrane protein CASK	*Cask*	1.04	Down	807730000	838217500
Peripherin-2	*Prph2*	1.11	Up	4867375000	4386950000
Peroxiredoxin-1	*Prdx1*	1.04	Up	10496625000	10096825000
Peroxiredoxin-2	*Prdx2*	1.05	Up	15367500000	14621000000
Peroxiredoxin-4	*Prdx4*	1.02	Down	1665866667	1701650000
Peroxiredoxin-5, mitochondrial	*Prdx5*	1.07	Up	6655500000	6232075000
Peroxiredoxin-6	*Prdx6*	1.09	Up	4013600000	3688525000
Peroxisomal biogenesis factor 19	*Pex19*	1.74	Up	42736000	24579000
Peroxisomal carnitine O-octanoyltransferase	*Crot*	1.02	Up	90828000	88717000
Peroxisomal membrane protein PEX14	*Pex14*	1.64	Up	101900000	62250000
Peroxisomal multifunctional enzyme type 2;(3R)-hydroxyacyl-CoA dehydrogenase;Enoyl-CoA hydratase 2	*Hsd17b4*	1.02	Down	926407500	943955000
PHD finger-like domain-containing protein 5A	*Phf5a*	1.23	Up	273633333.3	221690000
Phenylalanine--tRNA ligase alpha subunit	*Farsa*	1.12	Up	520910000	464580000
Phosducin	*Pdc*	1.07	Down	10900025000	11700500000
Phosphate carrier protein, mitochondrial	*Slc25a3*	1.09	Up	18515000000	17063250000
Phosphatidate cytidylyltransferase 1	*Cds1*	1.12	Up	983665000	877062500
Phosphatidate cytidylyltransferase 2	*Cds2*	1.34	Up	1115240000	834277500
Phosphatidylethanolamine-binding protein 1;Hippocampal cholinergic neurostimulating peptide	*Pebp1*	1.03	Up	23893000000	23255750000
Phosphatidylinositide phosphatase SAC1	*Sacm1l*	1.09	Up	881262500	811245000
Phosphatidylinositol 4-kinase alpha	*Pi4ka*	1.01	Up	832437500	822437500
Phosphatidylinositol 4-phosphate 5-kinase type-1 gamma	*Pip5k1c*	1.04	Up	518985000	500840000
Phosphatidylinositol 5-phosphate 4-kinase type-2 alpha	*Pip4k2a*	1.46	Down	222002500	324627500
Phosphatidylinositol 5-phosphate 4-kinase type-2 beta	*Pip4k2b*	1.01	Down	302567500	306692500
Phosphatidylinositol 5-phosphate 4-kinase type-2 gamma	*Pip4k2c*	1.24	Down	110366000	137370000
Phosphatidylinositol transfer protein alpha isoform	*Pitpna*	1.04	Down	3247025000	3376375000
Phosphatidylinositol transfer protein beta isoform	*Pitpnb*	1.20	Up	188926666.7	157795000
Phosphatidylinositol-binding clathrin assembly protein	*Picalm*	1.05	Down	259580000	272107500
Phosphatidylserine synthase 2	*Ptdss2*	1.31	Up	179525000	136620000
Phosphofurin acidic cluster sorting protein 1	*Pacs1*	1.07	Up	650940000	608555000
Phosphoglucomutase-1	*Pgm1*	1.05	Up	4730050000	4490350000
Phosphoglycerate kinase 1	*Pgk1*	1.03	Up	66919750000	64806750000
Phosphoglycerate mutase 1	*Pgam1*	1.02	Down	46590250000	47476250000
Phospholipase A-2-activating protein	*Plaa*	1.06	Up	516702500	489265000
Phospholipase D3	*Pld3*	1.03	Up	194367500	189055000
Phospholipid hydroperoxide glutathione peroxidase, mitochondrial;Phospholipid hydroperoxide glutathione peroxidase, nuclear	*Gpx4*	1.99	Up	1290360000	647730000
Phospholysine phosphohistidine inorganic pyrophosphate phosphatase	*Lhpp*	1.00	Up	162775000	162226666.7
Phosphoribosyl pyrophosphate synthase-associated protein 1	*Prpsap1*	1.24	Up	227710000	184147500
Phosphoribosyl pyrophosphate synthase-associated protein 2	*Prpsap2*	1.10	Up	1204050000	1094590000
Phosphoserine phosphatase	*Psph*	1.03	Up	409507500	396000000
Phytanoyl-CoA hydroxylase-interacting protein-like	*Phyhipl*	1.01	Down	1120167500	1136250000
Pirin	*Pir*	1.14	Down	367377500	419270000

Plasma membrane calcium-transporting ATPase 1	*Atp2b1*	1.06	Down	7239875000	7661550000
Plasma membrane calcium-transporting ATPase 2	*Atp2b2*	1.33	Up	682900000	512100000
Plasma membrane calcium-transporting ATPase 3	*Atp2b3*	1.05	Down	205330000	215202500
Plasma membrane calcium-transporting ATPase 4	*Atp2b4*	1.23	Up	228602500	185826666.7
Plasminogen activator inhibitor 1 RNA-binding protein;Plasminogen activator inhibitor 1 RNA-binding protein, N-terminally processed	*Serbp1*	1.49	Up	481907500	322722500
Plastin-3	*Pls3*	1.03	Up	351340000	342590000
Platelet-activating factor acetylhydrolase IB subunit alpha	*Pafah1b1*	1.04	Down	2787575000	2896425000
Platelet-activating factor acetylhydrolase IB subunit beta	*Pafah1b2*	1.11	Up	2671325000	2415375000
Platelet-activating factor acetylhydrolase IB subunit gamma	*Pafah1b3*	1.06	Down	1082735000	1145700000
Plectin	*Plec*	1.71	Down	554365000	949052500
Pleiotropic regulator 1	*Plrg1*	1.25	Down	225547500	282270000
Podocalyxin	*Podxl*	2.08	Up	119540500	57478000
Poly [ADP-ribose] polymerase 1	*Parp1*	1.06	Up	104040750	97950000
Poly(U)-binding-splicing factor PUF60	*Puf60*	1.01	Up	590407500	582837500
Polyadenylate-binding protein 1	*Pabpc1*	1.02	Up	4488575000	4415600000
Polyamine-modulated factor 1-binding protein 1	*Pmfbp1*	2.49	Up	755560000	302990000
Polymerase I and transcript release factor	*Ptrf*	1.08	Down	227135000	246232500
Polypyrimidine tract-binding protein 1	*Ptbp1*	1.10	Down	480707500	526535000
Polypyrimidine tract-binding protein 2	*Ptbp2*	1.05	Down	2037625000	2130300000
Potassium voltage-gated channel subfamily B member 1	*Kcnb1*	1.05	Up	419220000	400907500
Potassium voltage-gated channel subfamily C member 1	*Kcnc1*	1.29	Up	84663000	65568000
Potassium voltage-gated channel subfamily D member 3;Potassium voltage-gated channel subfamily D member 2	*Kcnd3;Kcnd2*	1.37	Up	38383000	28107000
Potassium/sodium hyperpolarization-activated cyclic nucleotide-gated channel 1;Potassium/sodium hyperpolarization-activated cyclic nucleotide-gated channel 2	*Hcn1;Hcn2*	1.05	Down	93197000	97808666.67
PRA1 family protein 3	*Arl6ip5*	1.08	Up	665080000	613415000
Prefoldin subunit 2	*Pfdn2*	1.05	Down	230910000	243370000
Prelamin-A/C;Lamin-A/C	*Lmna*	3.58	Down	341547500	1222425000
Pre-mRNA-processing factor 19	*Prpf19*	1.09	Down	1945900000	2124950000
Pre-mRNA-processing factor 6	*Prpf6*	1.25	Down	237737500	296080000
Prenylcysteine oxidase	*Pcyox1*	1.01	Down	1174602500	1180575000
Probable 2-oxoglutarate dehydrogenase E1 component DHKTD1, mitochondrial	*Dhtkd1*	1.51	Down	35048500	52998000
Probable ATP-dependent RNA helicase DDX46	*Ddx46*	1.19	Up	86621000	73016500
Probable tRNA N6-adenosine threonylcarbamoyltransferase	*Osgep*	1.05	Up	821992500	784485000
Profilin-1	*Pfn1*	1.23	Down	3765925000	4632450000
Profilin-2	*Pfn2*	1.00	Up	1105150000	1103732500
Programmed cell death 6-interacting protein	*Pdcd6ip*	1.01	Up	1154432500	1142300000
Programmed cell death protein 10	*Pdcd10*	1.08	Down	155300000	168080000
Prohibitin	*Phb*	1.09	Up	3456250000	3163800000
Prohibitin-2	*Phb2*	1.16	Up	4155075000	3568350000
Proliferation-associated protein 2G4	*Pa2g4*	1.02	Up	2475900000	2415575000
Prolyl endopeptidase	*Prep*	1.01	Down	534547500	541962500
Prolyl endopeptidase-like	*Prepl*	1.08	Down	488957500	529537500

Propionyl-CoA carboxylase alpha chain, mitochondrial	*Pcca*	1.10	Down	94450000	104328500
Prosaposin receptor GPR37	*Gpr37*	2.10	Up	671413333.3	319330000
Prostaglandin E synthase 3	*Ptges3*	1.25	Up	2511575000	2014825000
Prostaglandin reductase 1	*Ptgr1*	1.08	Up	2044300000	1897025000
Prostaglandin reductase 2	*Ptgr2*	1.12	Down	922227500	1036045000
Prostamide/prostaglandin F synthase	*Fam213b*	1.00	Down	136593333.3	136870000
Proteasomal ubiquitin receptor ADRM1	*Adrm1*	1.12	Up	206947500	184720000
Proteasome activator complex subunit 1	*Psme1*	1.02	Up	676907500	662377500
Proteasome inhibitor PI31 subunit	*Psmf1*	1.17	Down	148395000	173516666.7
Proteasome subunit alpha type-1	*Psma1*	1.23	Up	1759800000	1427575000
Proteasome subunit alpha type-2	*Psma2*	1.18	Up	1043697500	884835000
Proteasome subunit alpha type-3	*Psma3*	1.04	Up	1107260000	1060402500
Proteasome subunit alpha type-4	*Psma4*	1.14	Up	1438800000	1266175000
Proteasome subunit alpha type-5	*Psma5*	1.06	Down	1589950000	1678100000
Proteasome subunit alpha type-6	*Psma6*	1.12	Up	2515050000	2253375000
Proteasome subunit alpha type-7	*Psma7*	1.01	Down	1466475000	1476350000
Proteasome subunit beta type-1	*Psmb1*	1.06	Up	1702650000	1608625000
Proteasome subunit beta type-2	*Psmb2*	1.06	Down	1360275000	1443325000
Proteasome subunit beta type-3	*Psmb3*	1.02	Down	516677500	529585000
Proteasome subunit beta type-4	*Psmb4*	1.04	Up	892165000	858800000
Proteasome subunit beta type-5	*Psmb5*	1.02	Down	1274695000	1299925000
Proteasome subunit beta type-6	*Psmb6*	1.13	Up	1287175000	1137165000
Proteasome subunit beta type-7	*Psmb7*	1.04	Down	564902500	588460000
Protein arginine N-methyltransferase 1	*Prmt1*	1.08	Up	1460475000	1353650000
Protein argonaute-2	*Ago2*	1.06	Up	942862500	888445000
Protein bassoon	*Bsn*	1.16	Down	878947500	1019445000
Protein BUD31 homolog	*Bud31*	2.88	Down	28350666.67	81558275
Protein CutA	*Cuta*	1.09	Up	151860000	138907500
Protein deglycase DJ-1	*Park7*	1.02	Down	19528500000	19826000000
Protein DEK	*Dek*	1.08	Down	1469175000	1586500000
Protein disulfide-isomerase	*P4hb*	1.00	Up	3386525000	3375875000
Protein disulfide-isomerase A3	*Pdia3*	1.05	Up	12080250000	11466750000
Protein disulfide-isomerase A4	*Pdia4*	1.05	Down	458390000	480350000
Protein disulfide-isomerase A6	*Pdia6*	1.12	Up	1750825000	1558725000
Protein ERGIC-53	*Lman1*	1.05	Up	399817500	379477500
Protein FAM136A	*Fam136a*	1.35	Up	119780000	88574666.67
Protein FAM162A	*Fam162a*	1.01	Down	422300000	428365000
Protein farnesyltransferase subunit beta	*Fntb*	1.29	Down	35225000	45482000
Protein farnesyltransferase/geranylgeranyltransferase type-1 subunit alpha	*Fnta*	1.09	Up	243746666.7	223062500
Protein Hikeshi	*HIKESHI*	2.18	Down	38476000	84060000
Protein IMPACT	*Impact*	1.00	Up	1171175000	1166300000
Protein kinase C alpha type	*Prkca*	1.07	Up	3562300000	3336725000
Protein kinase C and casein kinase substrate in neurons 2 protein	*Pacsin2*	1.16	Down	401567500	465940000
Protein kinase C and casein kinase substrate in neurons protein 1	*Pacsin1*	1.04	Down	6257050000	6526125000
Protein kinase C beta type	*Prkcb*	1.14	Up	138233333.3	120790000
Protein kinase C delta-binding protein	*Prkcdbp*	1.16	Up	116170000	100139000
Protein kinase C epsilon type	*Prkce*	1.06	Down	399312500	423212500
Protein lin-7 homolog A	*Lin7a*	1.25	Down	1179140000	1474957500
Protein lin-7 homolog C	*Lin7c*	1.13	Down	593672500	670740000
Protein LYRIC	*Mtdh*	1.11	Up	156926666.7	141265000
Protein MEMO1	*Memo1*	1.20	Up	118333333.3	98373000
Protein NDRG1	*Ndrg1*	1.26	Down	1111040000	1398850000
Protein NDRG2	*Ndrg2*	1.06	Up	5605875000	5266450000
Protein NDRG3	*Ndrg3*	1.04	Down	553065000	575900000
Protein NDRG4	*Ndrg4*	1.15	Down	236507500	271947500
Protein O-GlcNAcase	*Mgea5*	1.34	Up	488135000	363450000
Protein OSCP1	*Oscp1*	1.47	Down	36332000	53496000
Protein pelota homolog	*Pelo*	1.21	Up	27770000	22960000

Protein phosphatase 1 regulatory subunit 7	*Ppp1r7*	1.09	Up	1627850000	1492325000
Protein phosphatase 1A	*Ppm1a*	1.02	Up	788775000	774885000
Protein phosphatase 1B	*Ppm1b*	1.09	Down	608352500	663637500
Protein phosphatase 1E	*Ppm1e*	1.05	Down	627067500	659900000
Protein phosphatase 1F	*Ppm1f*	1.21	Down	164513333.3	199640000
Protein phosphatase 1G	*Ppm1g*	1.14	Up	233922500	204920000
Protein phosphatase 1H	*Ppm1h*	1.00	Down	177426666.7	177913333.3
Protein phosphatase inhibitor 2	*Ppp1r2*	1.56	Down	170503333.3	265972500
Protein phosphatase methylesterase 1	*Ppme1*	1.06	Up	964700000	909240000
Protein piccolo	*Pclo*	1.31	Up	843476666.7	642910000
Protein prune homolog 2	*Prune2*	1.07	Down	118271000	126069750
Protein Red	*Ik*	1.28	Down	62397666.67	79752000
Protein RER1	*Rer1*	1.13	Down	1316300000	1484400000
Protein rogdi homolog	*Rogdi*	1.07	Up	406275000	380302500
Protein RUFY3	*Rufy3*	1.17	Up	2018600000	1731450000
Protein S100-B	*S100b*	1.22	Up	351300000	288945000
Protein SCAF8	*Scaf8*	1.08	Up	161740000	149805000
Protein SEC13 homolog	*Sec13*	1.17	Up	388720000	333227500
Protein sel-1 homolog 1	*Sel1l*	1.05	Up	96554500	91771333.33
Protein SET	*Set*	1.11	Up	1895800000	1709850000
Protein TBRG4	*Tbrg4*	1.22	Up	119392750	97522000
Protein THEM6	*Them6*	1.56	Up	98520000	63031000
Protein transport protein Sec31A	*Sec31a*	1.05	Down	913962500	963597500
Protein transport protein Sec61 subunit alpha isoform 1	*Sec61a1*	1.02	Down	142710000	146092500
Protein TSSC1	*Tssc1*	1.15	Down	80587000	92532000
Protein tweety homolog 1	*Ttyh1*	1.02	Up	1536875000	1513600000
Protein tyrosine phosphatase type IVA 2	*Ptp4a2*	1.05	Up	226600000	216045000
Protein unc-119 homolog A	*Unc119*	1.07	Down	1857225000	1993450000
Protein VAC14 homolog	*Vac14*	1.02	Down	132955000	135232500
Protein-arginine deiminase type-2	*Padi2*	1.16	Up	984100000	846582500
Protein-L-isoaspartate(D-aspartate) O-methyltransferase	*Pcmt1*	1.06	Down	1094005000	1163725000
Protein-S-isoprenylcysteine O-methyltransferase	*Icmt*	1.35	Up	154280000	113876666.7
Proto-oncogene tyrosine-protein kinase Src	*Src*	1.12	Up	338107500	300860000
Purine nucleoside phosphorylase	*Pnp*	1.15	Up	1709825000	1485625000
Putative ATP-dependent RNA helicase DHX30	*Dhx30*	1.17	Up	40738333.33	34917000
Putative hydrolase RBBP9	*Rbbp9*	1.09	Up	331876666.7	303482500
Pyridoxal kinase	*Pdxk*	1.08	Up	4256450000	3931000000
Pyridoxal phosphate phosphatase	*Pdxp*	1.05	Up	2848325000	2707100000
Pyridoxine-5-phosphate oxidase	*Pnpo*	1.42	Down	422367500	598005000
Pyrroline-5-carboxylate reductase 2	*Pycr2*	1.39	Up	151030000	108680000
Pyrroline-5-carboxylate reductase 3	*Pycrl*	1.11	Down	311820000	346012500
Pyruvate carboxylase, mitochondrial	*Pc*	1.08	Up	1964475000	1826250000
Pyruvate dehydrogenase E1 component subunit alpha, somatic form, mitochondrial	*Pdha1*	1.20	Down	3426450000	4103825000
Pyruvate dehydrogenase E1 component subunit beta, mitochondrial	*Pdhb*	1.02	Up	5552625000	5459925000
Pyruvate kinase PKM	*Pkm*	1.04	Up	1.1203E+11	1.07333E+11
Quinone oxidoreductase	*Cryz*	1.13	Up	1008857500	896560000
Rab GDP dissociation inhibitor alpha	*Gdi1*	1.04	Up	30119000000	28842750000
Rab GDP dissociation inhibitor beta	*Gdi2*	1.19	Up	9922000000	8315600000
Rab GTPase-binding effector protein 1	*Rabep1*	1.12	Down	81467000	91085000
Rab3 GTPase-activating protein catalytic subunit	*Rab3gap1*	1.44	Up	126970000	88474750
Rab3 GTPase-activating protein non-catalytic subunit	*Rab3gap2*	1.30	Down	151643333.3	197082500
Rabphilin-3A	*Rph3a*	1.07	Down	1413460000	1516350000
RAC-alpha serine/threonine-protein kinase	*Akt1*	1.06	Up	194075000	182420000
Ragulator complex protein LAMTOR1	*Lamtor1*	1.03	Up	179535000	173722500

Ral GTPase-activating protein subunit alpha-1	*Ralgapa1*	1.11	Up	47257000	42628333.33
Ras-related C3 botulinum toxin substrate 1	*Rac1*	1.03	Down	4228875000	4358425000
Ras-related GTP-binding protein A	*Rraga*	1.09	Up	173720000	159337500
Ras-related protein Rab-10	*Rab10*	1.23	Up	619697500	501837500
Ras-related protein Rab-11B;Ras-related protein Rab-11A	*Rab11b;Rab11a*	1.09	Down	2444725000	2668675000
Ras-related protein Rab-12	*Rab12*	1.15	Down	71557000	82275000
Ras-related protein Rab-14	*Rab14*	1.04	Up	2872900000	2749950000
Ras-related protein Rab-18	*Rab18*	1.03	Up	1140630000	1110900000
Ras-related protein Rab-1A	*Rab1A*	1.01	Down	4676425000	4700525000
Ras-related protein Rab-1B	*Rab1b*	1.02	Down	1315775000	1347625000
Ras-related protein Rab-21	*Rab21*	1.24	Down	493895000	612417500
Ras-related protein Rab-28	*Rab28*	1.07	Up	157527500	147850000
Ras-related protein Rab-2A	*Rab2a*	1.07	Up	3218950000	3005750000
Ras-related protein Rab-35	*Rab35*	1.55	Down	372712500	576117500
Ras-related protein Rab-3A	*Rab3a*	1.15	Up	9262950000	8025975000
Ras-related protein Rab-3B	*Rab3b*	1.20	Up	645006666.7	536982500
Ras-related protein Rab-3C	*Rab3c*	1.48	Up	681147500	459653333.3
Ras-related protein Rab-4B	*Rab4b*	1.02	Down	134517500	136763333.3
Ras-related protein Rab-5A	*Rab5a*	1.01	Down	1983875000	1997175000
Ras-related protein Rab-6A	*Rab6a*	1.27	Down	4471200000	5688000000
Ras-related protein Rab-7a	*Rab7a*	1.07	Up	4918400000	4591250000
Ras-related protein Rab-8A	*Rab8a*	1.04	Up	153575000	148186666.7
Ras-related protein Rab-8B	*Rab8b*	1.46	Up	96372000	65839000
Ras-related protein Ral-A	*Rala*	1.11	Up	1267425000	1138492500
Ras-related protein Ral-B	*Ralb*	1.29	Down	903775000	1165927500
Ras-related protein Rap-1A	*Rap1a*	1.03	Down	1882400000	1930500000
Ras-related protein Rap-1b	*Rap1b*	1.11	Down	406467500	449605000
Ras-related protein R-Ras	*Rras*	1.16	Up	179485000	155226666.7
Receptor expression-enhancing protein 5	*Reep5*	1.23	Down	484420000	594210000
Receptor expression-enhancing protein 6	*Reep6*	1.03	Down	1357032500	1398500000
Receptor-type tyrosine-protein phosphatase alpha	*Ptpra*	2.17	Down	26060000	56626000
Receptor-type tyrosine-protein phosphatase F	*Ptprf*	1.09	Down	445202500	486040000
Receptor-type tyrosine-protein phosphatase zeta	*Ptprz1*	1.21	Down	74303666.67	90118500
Redox-regulatory protein FAM213A	*Fam213a*	1.10	Up	590447500	535132500
Regulator of G-protein signaling 7	*Rgs7*	1.11	Down	556655000	620267500
Regulator of G-protein signaling 9	*Rgs9*	1.22	Up	820292500	672115000
Regulator of microtubule dynamics protein 3	*Rmdn3*	1.00	Up	70074000	70068750
Renin receptor	*Atp6ap2*	1.13	Down	383755000	434572500
Replication initiator 1	*Repin1*	1.08	Down	10880500	11731500
Replication protein A 32 kDa subunit	*Rpa2*	1.09	Down	244867500	267005000
Reticulon-1	*Rtn1*	1.12	Down	3390725000	3789475000
Reticulon-3	*Rtn3*	1.05	Up	3169825000	3009250000
Reticulon-4	*Rtn4*	1.03	Up	8819375000	8600650000
Retinal dehydrogenase 1	*Aldh1a1*	11.69	Down	91349750	1067577500
Retinol-binding protein 1	*Rbp1*	1.10	Up	1912400000	1735725000
Rho GDP-dissociation inhibitor 1	*Arhgdia*	1.06	Down	3834700000	4078575000
Rho GTPase-activating protein 44	*Arhgap44*	1.34	Down	84239500	112710000
Rho guanine nucleotide exchange factor 9	*Arhgef9*	1.13	Down	100960000	114410000
Rhodopsin	*Rho*	1.08	Up	26407750000	24357750000
Rhodopsin kinase	*Grk1*	1.02	Down	2535050000	2588100000
Rho-related GTP-binding protein RhoB	*Rhob*	1.05	Down	215515000	225445000
Ribonuclease inhibitor	*Rnh1*	1.08	Up	827290000	768890000
Ribonuclease UK114	*Hrsp12*	1.09	Down	646263333.3	704910000
Ribose-phosphate pyrophosphokinase 1;Ribose-phosphate pyrophosphokinase 2	*Prps1;Prps2*	1.08	Down	2841600000	3058025000
Ribosomal protein S6 kinase alpha-1	*Rps6ka1*	1.11	Up	66309500	59865000
Ribosome maturation protein SBDS	*Sbds*	1.48	Down	74987000	111352750

RNA polymerase II subunit A C-terminal domain phosphatase SSU72	*Ssu72*	1.03	Down	53078750	54678666.67
RNA polymerase-associated protein LEO1	*Leo1*	1.22	Down	81605500	99152333.33
RNA-binding motif protein, X chromosome retrogene-like;RNA-binding motif protein, X chromosome retrogene-like, N-terminally processed;RNA binding motif protein, X-linked-like-1	*Rbmxrtl;Rbmxl1*	3.02	Up	232250000	76812000
RNA-binding motif protein, X chromosome;RNA-binding motif protein, X chromosome, N-terminally processed	*Rbmx*	1.02	Down	1961400000	2001475000
RNA-binding protein 10	*Rbm10*	1.48	Up	131406500	89069500
RNA-binding protein 3	*Rbm3*	1.08	Down	200910000	216060000
RNA-binding protein 4B	*Rbm4b*	1.06	Up	191100000	180340000
RNA-binding protein 8A	*Rbm8a*	1.52	Down	254630000	386995000
RNA-binding protein Musashi homolog 1	*Msi1*	1.09	Down	1970075000	2144100000
RNA-binding protein Nova-1	*Nova1*	1.07	Down	120321750	128301750
Rod outer segment membrane protein 1	*Rom1*	1.00	Up	1949975000	1941050000
rRNA 2-O-methyltransferase fibrillarin	*Fbl*	1.07	Up	256220000	239622500
RUS1 family protein C16orf58 homolog	*N/A*	1.04	Down	136480000	141870000
RuvB-like 1	*Ruvbl1*	1.08	Up	918832500	853235000
Ryanodine receptor 2	*Ryr2*	1.11	Down	46720500	51808000
Saccharopine dehydrogenase-like oxidoreductase	*Sccpdh*	1.09	Up	1352350000	1240350000
S-adenosylmethionine synthase isoform type-2	*Mat2a*	1.32	Down	508395000	671030000
SAP domain-containing ribonucleoprotein	*Sarnp*	1.23	Down	944910000	1161200000
Sarcolemmal membrane-associated protein	*Slmap*	1.20	Up	3605025000	2996700000
Sarcoplasmic/endoplasmic reticulum calcium ATPase 2	*Atp2a2*	1.03	Down	3168775000	3258175000
Sarcosine dehydrogenase, mitochondrial	*Sardh*	1.34	Down	82565000	110309750
S-arrestin	*Sag*	1.21	Up	96800750000	79675250000
Scaffold attachment factor B1	*Safb*	1.02	Up	461730000	454822500
Sec1 family domain-containing protein 1	*Scfd1*	1.02	Down	196523333.3	200697500
SEC14-like protein 2	*Sec14l2*	1.37	Up	175510000	127936666.7
Secernin-1	*Scrn1*	1.14	Up	2253975000	1977525000
Secernin-2	*Scrn2*	1.03	Down	429765000	443240000
Secretagogin	*Scgn*	1.03	Down	1755000000	1805575000
Secretory carrier-associated membrane protein 1	*Scamp1*	1.03	Up	1431850000	1395725000
Secretory carrier-associated membrane protein 5	*Scamp5*	1.16	Up	598747500	514072500
Selenium-binding protein 1	*Selenbp1*	1.02	Down	123290000	125687500
Selenocysteine lyase	*Scly*	1.15	Down	257877500	295410000
Selenoprotein T	*Selt*	1.16	Down	96403666.67	111700000
Sepiapterin reductase	*Spr*	1.00	Up	148627500	148045000
Septin-11	*Sep--11*	1.06	Down	2299300000	2437950000
Septin-2	*Sep--2*	1.07	Up	1123650000	1046535000
Septin-5	*Sep--5*	1.03	Up	2498075000	2416925000
Septin-7	*Sep--7*	1.46	Up	3596500000	2466075000
Septin-8	*Sep--8*	1.00	Down	602317500	604175000
Septin-9	*Sep--9*	1.15	Up	516305000	447475000
Serine protease inhibitor A3K	*Serpina3k*	1.51	Down	132024666.7	199932500
Serine/arginine-rich splicing factor 2	*Srsf2*	1.11	Up	1065990000	958195000
Serine/arginine-rich splicing factor 5	*Srsf5*	1.05	Down	320243333.3	336552500
Serine/arginine-rich splicing factor 6	*Srsf6*	1.04	Down	748063333.3	781485000
Serine/threonine-protein kinase 24;Serine/threonine-protein kinase 24 35 kDa subunit;Serine/threonine-protein kinase 24 12 kDa subunit	*Stk24*	1.61	Up	104294333.3	64753000
Serine/threonine-protein kinase A-Raf	*Araf*	1.37	Up	83004000	60522000
Serine/threonine-protein kinase BRSK2	*Brsk2*	1.07	Up	208770000	195107500
Serine/threonine-protein kinase DCLK1	*Dclk1*	1.25	Down	306647500	384375000

Serine/threonine-protein kinase DCLK2	*Dclk2*	1.01	Up	165895000	163965000
Serine/threonine-protein kinase MARK2	*Mark2*	1.33	Up	40142500	30255500
Serine/threonine-protein kinase mTOR	*Mtor*	1.15	Up	230965000	201340000
Serine/threonine-protein kinase PAK 2;PAK-2p27;PAK-2p34	*Pak2*	1.20	Down	223215000	266893333.3
Serine/threonine-protein kinase PAK 3	*Pak3*	1.08	Down	1514900000	1629750000
Serine/threonine-protein kinase TAO3	*Taok3*	1.30	Down	118640000	154710000
Serine/threonine-protein kinase WNK1;Serine/threonine-protein kinase WNK4	*Wnk1;Wnk4*	1.03	Up	58340000	56867000
Serine/threonine-protein phosphatase 2A 55 kDa regulatory subunit B alpha isoform	*Ppp2r2a*	1.03	Up	337285000	328932500
Serine/threonine-protein phosphatase 2A 65 kDa regulatory subunit A beta isoform	*Ppp2r1b*	1.06	Up	1155500000	1094775000
Serine/threonine-protein phosphatase 2A catalytic subunit alpha isoform	*Ppp2ca*	1.08	Up	3995775000	3705925000
Serine/threonine-protein phosphatase 2B catalytic subunit alpha isoform	*Ppp3ca*	1.08	Up	1322000000	1224275000
Serine/threonine-protein phosphatase 2B catalytic subunit beta isoform	*Ppp3cb*	1.05	Up	230286666.7	218947500
Serine/threonine-protein phosphatase 4 catalytic subunit	*Ppp4c*	1.21	Down	112535000	135670000
Serine/threonine-protein phosphatase 5	*Ppp5c*	1.04	Up	593802500	571602500
Serine/threonine-protein phosphatase 6 catalytic subunit;Serine/threonine-protein phosphatase 6 catalytic subunit, N-terminally processed	*Ppp6c*	1.19	Down	196230000	234075000
Serine/threonine-protein phosphatase CPPED1	*Cpped1*	1.06	Down	82469000	87428333.33
Serine/threonine-protein phosphatase PGAM5, mitochondrial	*Pgam5*	1.27	Down	96226500	122074666.7
Serine/threonine-protein phosphatase PP1-alpha catalytic subunit	*Ppp1ca*	1.18	Down	221720000	261602500
Serine/threonine-protein phosphatase PP1-beta catalytic subunit	*Ppp1cb*	1.28	Up	442220000	344995000
Serine/threonine-protein phosphatase PP1-gamma catalytic subunit	*Ppp1cc*	1.09	Up	4121775000	3783200000
Serine-threonine kinase receptor-associated protein	*Strap*	1.13	Down	700285000	794575000
Serine--tRNA ligase, cytoplasmic	*Sars*	1.05	Up	1865950000	1775475000
Serotransferrin	*Tf*	1.01	Up	3547475000	3514350000
Serpin H1	*Serpinh1*	1.61	Up	193510000	120133333.3
Serum albumin	*Alb*	1.03	Down	19192000000	19850750000
Serum paraoxonase/arylesterase 2	*Pon2*	1.19	Up	303880000	255890000
S-formylglutathione hydrolase	*Esd*	1.01	Up	3316125000	3298700000
SH3 domain-containing kinase-binding protein 1	*Sh3kbp1*	1.86	Up	47868000	25696500
SH3-containing GRB2-like protein 3-interacting protein 1	*Sgip1*	1.02	Down	237450000	241025000
Shootin-1	*SHTN1*	1.10	Up	31828500	29052500
Short/branched chain specific acyl-CoA dehydrogenase, mitochondrial	*Acadsb*	1.03	Down	165245000	170806666.7
Sideroflexin-1	*Sfxn1*	1.00	Up	1202000000	1201102500
Sideroflexin-3	*Sfxn3*	1.04	Up	4003750000	3837475000
Sideroflexin-5	*Sfxn5*	1.06	Up	1213290000	1140100000
Signal recognition particle 54 kDa protein	*Srp54*	1.08	Up	187326666.7	173645000
Signal recognition particle receptor subunit beta	*Srprb*	1.06	Down	324102500	342970000
Signal transducer and activator of transcription 3	*Stat3*	1.15	Up	333137500	289377500
Signal transducer and activator of transcription 5B;Signal transducer and activator of transcription 5A	*Stat5b;Stat5a*	1.24	Up	337037500	272312500

Single-stranded DNA-binding protein, mitochondrial	*Ssbp1*	1.14	Up	338435000	296365000
Sister chromatid cohesion protein PDS5 homolog B	*Pds5b*	1.07	Up	288365000	269365000
Small glutamine-rich tetratricopeptide repeat-containing protein alpha	*Sgta*	1.10	Down	571772500	626810000
Small nuclear ribonucleoprotein-associated protein N;Small nuclear ribonucleoprotein-associated protein B	*Snrpn;Snrpb*	1.09	Down	1284407500	1403950000
Sodium- and chloride-dependent GABA transporter 1	*Slc6a1*	1.16	Up	2573050000	2213350000
Sodium- and chloride-dependent GABA transporter 3	*Slc6a11*	1.01	Down	4406025000	4431925000
Sodium- and chloride-dependent glycine transporter 1	*Slc6a9*	1.01	Down	1246575000	1260000000
Sodium- and chloride-dependent taurine transporter	*Slc6a6*	1.06	Down	121843333.3	128672500
Sodium bicarbonate cotransporter 3	*Slc4a7*	1.17	Up	451202500	384570000
Sodium channel subunit beta-2	*Scn2b*	1.30	Down	123630000	161150000
Sodium/calcium exchanger 1	*Slc8a1*	1.03	Down	527492500	541127500
Sodium/calcium exchanger 3	*Slc8a3*	1.13	Up	117280000	104222500
Sodium/potassium/calcium exchanger 1	*Slc24a1*	1.32	Down	177730000	235337500
Sodium/potassium-transporting ATPase subunit alpha-1	*Atp1a1*	1.06	Up	19515750000	18458750000
Sodium/potassium-transporting ATPase subunit alpha-2	*Atp1a2*	1.04	Up	1241300000	1197500000
Sodium/potassium-transporting ATPase subunit alpha-3	*Atp1a3*	1.01	Down	80839250000	81326500000
Sodium/potassium-transporting ATPase subunit beta-1	*Atp1b1*	1.00	Down	9784775000	9814050000
Sodium/potassium-transporting ATPase subunit beta-2	*Atp1b2*	1.05	Up	2520100000	2407200000
Sodium/potassium-transporting ATPase subunit beta-3	*Atp1b3*	1.12	Up	6050200000	5397725000
Sodium-coupled neutral amino acid transporter 1	*Slc38a1*	1.01	Down	138213333.3	139800000
Sodium-coupled neutral amino acid transporter 3	*Slc38a3*	1.14	Up	896325000	784575000
Sodium-dependent neutral amino acid transporter SLC6A17	*Slc6a17*	1.21	Up	260710000	215350000
Sodium-driven chloride bicarbonate exchanger	*Slc4a10*	1.13	Up	728967500	643257500
Solute carrier family 12 member 4	*Slc12a4*	1.27	Up	79625500	62519000
Solute carrier family 12 member 5	*Slc12a5*	1.06	Up	17085000000	16092500000
Solute carrier family 12 member 7	*Slc12a7*	1.20	Up	183110000	152080000
Solute carrier family 2, facilitated glucose transporter member 1	*Slc2a1*	1.03	Down	3231675000	3329350000
Solute carrier family 2, facilitated glucose transporter member 3	*Slc2a3*	1.07	Down	321717500	344862500
Solute carrier family 25 member 46	*Slc25a46*	1.24	Up	48822000	39464500
Sorbitol dehydrogenase	*Sord*	1.05	Up	246646666.7	235005000
Sorting and assembly machinery component 50 homolog	*Samm50*	1.01	Up	654022500	650327500
Sorting nexin-1	*Snx1*	1.04	Down	416315000	432150000
Sorting nexin-27	*Snx27*	1.15	Up	542395000	471720000
Sorting nexin-3	*Snx3*	1.02	Up	423475000	413987500
Sorting nexin-5	*Snx5*	1.18	Down	180912500	214070000
SPARC	*Sparc*	1.02	Up	136928333.3	133952500
Spectrin alpha chain, non-erythrocytic 1	*Sptan1*	1.06	Down	26600500000	28065500000
Spectrin beta chain, non-erythrocytic 2	*Sptbn2*	1.02	Down	2536825000	2600125000
Spermatid perinuclear RNA-binding protein	*Strbp*	1.11	Down	327503333.3	363865000
S-phase kinase-associated protein 1	*Skp1*	1.28	Down	571392500	733552500
Spliceosome RNA helicase Ddx39b	*Ddx39b*	1.07	Up	13133250000	12272250000

Splicing factor 3A subunit 2	*Sf3a2*	1.04	Down	352065000	367212500
Splicing factor U2AF 26 kDa subunit	*U2af1l4*	1.06	Down	348540000	369882500
SPRY domain-containing protein 4	*Spryd4*	1.16	Up	189885000	164257500
Squalene synthase	*Fdft1*	1.00	Down	140610000	140670000
Src substrate cortactin	*Cttn*	1.25	Down	551412500	690572500
STAM-binding protein	*Stambp*	1.11	Down	77872000	86784000
Staphylococcal nuclease domain-containing protein 1	*Snd1*	1.00	Down	1160075000	1165675000
Stathmin	*Stmn1*	1.12	Up	336470000	300377500
STE20/SPS1-related proline-alanine-rich protein kinase	*Stk39*	1.12	Down	109460000	122720000
Steroid hormone receptor ERR2	*Esrrb*	1.37	Up	71196500	52100000
Sterol-4-alpha-carboxylate 3-dehydrogenase, decarboxylating	*Nsdhl*	1.22	Down	104900000	127516666.7
Stomatin-like protein 2, mitochondrial	*Stoml2*	1.16	Up	315772500	271677500
Stress-70 protein, mitochondrial	*Hspa9*	1.01	Down	7398450000	7493350000
Stress-induced-phosphoprotein 1	*Stip1*	1.03	Up	4715325000	4584025000
Striatin-3	*Strn3*	1.04	Down	152775000	158786666.7
Stromal interaction molecule 1	*Stim1*	1.15	Up	165247500	143662500
Structural maintenance of chromosomes protein 1A	*Smc1a*	1.34	Down	611392500	819747500
Structural maintenance of chromosomes protein 3	*Smc3*	1.04	Down	581857500	605362500
Succinate dehydrogenase [ubiquinone] cytochrome b small subunit, mitochondrial	*Sdhd*	1.19	Up	163987250	138220666.7
Succinate dehydrogenase [ubiquinone] flavoprotein subunit, mitochondrial	*Sdha*	1.17	Up	3935325000	3368800000
Succinate dehydrogenase [ubiquinone] iron-sulfur subunit, mitochondrial	*Sdhb*	1.06	Up	1604900000	1508050000
Succinate-semialdehyde dehydrogenase, mitochondrial	*Aldh5a1*	1.02	Down	4163250000	4255475000
Succinyl-CoA ligase [ADP/GDP-forming] subunit alpha, mitochondrial	*Suclg1*	1.18	Down	3339200000	3938100000
Succinyl-CoA:3-ketoacid coenzyme A transferase 1, mitochondrial	*Oxct1*	1.16	Down	1127907500	1306827500
Sulfated glycoprotein 1	*Psap*	1.01	Down	509285000	515987500
Sulfite oxidase, mitochondrial	*Suox*	1.02	Up	234365000	229715000
Sulfotransferase 4A1	*Sult4a1*	1.05	Up	87122500	82898500
SUMO-activating enzyme subunit 1;SUMO-activating enzyme subunit 1, N-terminally processed	*Sae1*	1.12	Up	1472700000	1320275000
SUMO-conjugating enzyme UBC9	*Ube2i*	1.04	Up	839395000	807437500
Superoxide dismutase [Cu-Zn]	*Sod1*	1.34	Down	6953700000	9313725000
Superoxide dismutase [Mn], mitochondrial	*Sod2*	1.26	Down	3240400000	4075425000
Suppressor of G2 allele of SKP1 homolog	*Sugt1*	1.06	Down	360217500	380930000
Survival of motor neuron-related-splicing factor 30	*Smndc1*	1.14	Down	135375000	153753333.3
SWI/SNF-related matrix-associated actin-dependent regulator of chromatin subfamily E member 1	*Smarce1*	1.14	Down	88763000	100844250
Synapsin-1	*Syn1*	1.05	Up	4880575000	4627325000
Synapsin-2	*Syn2*	1.09	Up	1732950000	1589400000
Synaptic vesicle glycoprotein 2A	*Sv2a*	1.12	Down	4487575000	5012825000
Synaptic vesicle glycoprotein 2B	*Sv2b*	1.05	Up	3880975000	3691600000
Synaptic vesicle membrane protein VAT-1 homolog	*Vat1*	1.04	Down	1342875000	1390625000
Synaptobrevin homolog YKT6	*Ykt6*	1.04	Down	214685000	224200000
Synaptogyrin-1	*Syngr1*	1.14	Up	1517000000	1326300000
Synaptojanin-1	*Synj1*	1.01	Up	6085625000	6003750000
Synaptojanin-2-binding protein	*Synj2bp*	1.26	Up	131640000	104662750
Synaptophysin	*Syp*	1.02	Up	6568025000	6432950000
Synaptoporin	*Synpr*	1.31	Up	3362525000	2575875000
Synaptosomal-associated protein 25	*Snap25*	1.38	Down	1777875000	2458325000

Synaptotagmin-1	*Syt1*	1.07	Up	20616500000	19315500000
Synergin gamma	*Synrg*	1.28	Up	77271750	60272500
Syntaxin-12	*Stx12*	1.09	Up	564302500	518855000
Syntaxin-1A	*Stx1a*	1.13	Down	153806666.7	174343333.3
Syntaxin-1B	*Stx1b*	1.03	Down	4578000000	4723000000
Syntaxin-3	*Stx3*	1.08	Up	2040875000	1898100000
Syntaxin-4	*Stx4*	1.07	Up	44740000	41763250
Syntaxin-7	*Stx7*	1.08	Down	244536666.7	263545000
Syntaxin-binding protein 1	*Stxbp1*	1.03	Up	32669000000	31744500000
Syntaxin-binding protein 5	*Stxbp5*	1.05	Up	515200000	489430000
Syntenin-1	*Sdcbp*	1.08	Up	278740000	258766666.7
Tail-anchored protein insertion receptor WRB	*Wrb*	1.06	Down	83905500	88536500
Target of rapamycin complex subunit LST8	*Mlst8*	1.38	Up	62205000	45208000
T-complex protein 1 subunit alpha	*Tcp1*	1.02	Up	6685625000	6527375000
T-complex protein 1 subunit beta	*Cct2*	1.02	Up	5406450000	5325475000
T-complex protein 1 subunit delta	*Cct4*	1.03	Up	6755650000	6556350000
T-complex protein 1 subunit epsilon	*Cct5*	1.04	Up	5777400000	5572925000
T-complex protein 1 subunit gamma	*Cct3*	1.01	Down	7184175000	7277150000
Tectonin beta-propeller repeat-containing protein 1	*Tecpr1*	1.21	Up	164236666.7	135565000
Tenascin-R	*Tnr*	1.05	Up	1080647500	1030092500
Tetratricopeptide repeat protein 9C	*Ttc9c*	1.21	Up	76186000	63159000
Thiamine-triphosphatase	*Thtpa*	1.47	Down	98002000	144545000
Thimet oligopeptidase	*Thop1*	1.08	Up	2974025000	2743875000
Thioredoxin	*Txn*	1.25	Down	879995000	1098555000
Thioredoxin domain-containing protein 12	*Txndc12*	1.04	Down	296112500	308317500
Thioredoxin domain-containing protein 9	*Txndc9*	1.04	Up	70813000	67984500
Thioredoxin reductase 1, cytoplasmic	*Txnrd1*	1.03	Up	836972500	816035000
Thioredoxin reductase 2, mitochondrial	*Txnrd2*	1.44	Down	48753000	70265750
Thioredoxin-dependent peroxide reductase, mitochondrial	*Prdx3*	1.22	Up	1675425000	1378250000
Thioredoxin-like protein 1	*Txnl1*	1.02	Down	915105000	936362500
Thioredoxin-related transmembrane protein 2	*Tmx2*	1.04	Down	137690000	143350000
Thiosulfate sulfurtransferase	*Tst*	1.11	Up	859970000	772370000
THO complex subunit 6 homolog	*Thoc6*	2.04	Up	80596000	39597500
Threonine--tRNA ligase, cytoplasmic	*Tars*	1.18	Up	1267650000	1075850000
Threonine--tRNA ligase, mitochondrial	*Tars2*	1.59	Up	84227500	52861000
Thy-1 membrane glycoprotein	*Thy1*	1.06	Up	2387200000	2245575000
Thyroid hormone receptor-associated protein 3	*Thrap3*	1.11	Down	88340500	97908000
T-kininogen 1;T-kininogen 1 heavy chain;T-kinin;T-kininogen 1 light chain	*Map1*	1.20	Up	151596666.7	126384000
Toll-interacting protein	*Tollip*	1.01	Down	572852500	577695000
Torsin-1A-interacting protein 1	*Tor1aip1*	1.03	Down	307905000	315810000
Trafficking protein particle complex subunit 1	*Trappc1*	1.30	Up	286165000	220397500
Trafficking protein particle complex subunit 2	*Trappc2*	1.00	Up	160100000	160025000
Trafficking protein particle complex subunit 2-like protein	*Trappc2l*	1.08	Down	63559000	68520500
Trafficking protein particle complex subunit 3	*Trappc3*	1.05	Down	340727500	358202500
Trafficking protein particle complex subunit 4	*Trappc4*	1.07	Up	124727500	116191500
Transaldolase	*Taldo1*	1.01	Up	10657475000	10560425000
Transcription activator BRG1	*Smarca4*	1.21	Up	505662500	416695000
Transcription elongation factor A protein 1	*Tcea1*	1.01	Down	620927500	629307500
Transcription elongation factor B polypeptide 1	*Tceb1*	1.07	Up	619627500	576407500

Transcription elongation factor B polypeptide 2	*Tceb2*	1.01	Down	695107500	700152500
Transcription initiation factor IIB	*Gtf2b*	1.46	Down	78263333.33	114628666.7
Transcription intermediary factor 1-beta	*Trim28*	1.06	Up	3449475000	3257400000
Transcriptional activator protein Pur-alpha	*Pura*	1.16	Down	2988525000	3456550000
Transcriptional activator protein Pur-beta	*Purb*	1.11	Down	2697150000	3006925000
Transcriptional repressor CTCF	*Ctcf*	1.90	Down	34962666.67	66479250
Transferrin receptor protein 1	*Tfrc*	1.05	Up	99044250	94668750
Transformer-2 protein homolog beta	*Tra2b*	1.21	Up	375363333.3	310995000
Transforming protein RhoA	*Rhoa*	1.08	Down	2619425000	2833125000
Transgelin	*Tagln*	1.11	Down	503730000	557707500
Transgelin-2	*Tagln2*	1.06	Up	710737500	671445000
Transgelin-3	*Tagln3*	1.08	Down	3464500000	3730650000
Transitional endoplasmic reticulum ATPase	*Vcp*	1.05	Up	15793000000	15104250000
Transketolase	*Tkt*	1.01	Up	37281250000	36774250000
Translation initiation factor eIF-2B subunit alpha	*Eif2b1*	1.02	Up	94491000	92966333.33
Translation initiation factor eIF-2B subunit beta	*Eif2b2*	1.01	Down	135920000	136650000
Translation initiation factor eIF-2B subunit delta	*Eif2b4*	1.10	Down	95128000	104539000
Translation initiation factor eIF-2B subunit epsilon	*Eif2b5*	1.16	Down	73034000	84616000
Translationally-controlled tumor protein	*Tpt1*	1.02	Up	1307100000	1277525000
Translin-associated protein X	*Tsnax*	1.19	Up	928185000	782005000
Translocon-associated protein subunit alpha	*Ssr1*	1.31	Up	270030000	206600000
Translocon-associated protein subunit delta	*Ssr4*	1.03	Up	375200000	362737500
Transmembrane 9 superfamily member 2	*Tm9sf2*	1.13	Up	212426666.7	188177500
Transmembrane emp24 domain-containing protein 10	*Tmed10*	1.15	Up	935800000	817102500
Transmembrane emp24 domain-containing protein 2	*Tmed2*	1.18	Up	250300000	211665000
Transmembrane emp24 domain-containing protein 7	*Tmed7*	1.08	Up	617145000	571037500
Transmembrane emp24 domain-containing protein 9	*Tmed9*	1.10	Up	360995000	328862500
Transmembrane protein 11, mitochondrial	*Tmem11*	1.07	Up	306173333.3	285792500
Transmembrane protein 33	*Tmem33*	1.12	Up	159195000	142597500
Transmembrane protein 43	*Tmem43*	1.05	Down	83275333.33	87113000
Tricarboxylate transport protein, mitochondrial	*Slc25a1*	1.12	Up	480727500	428960000
Trifunctional enzyme subunit alpha, mitochondrial;Long-chain enoyl-CoA hydratase;Long chain 3-hydroxyacyl-CoA dehydrogenase	*Hadha*	1.15	Up	3086000000	2682700000
Trifunctional enzyme subunit beta, mitochondrial;3-ketoacyl-CoA thiolase	*Hadhb*	1.08	Up	1779450000	1647400000
Triosephosphate isomerase	*Tpi1*	1.00	Up	64398250000	64240000000
Tripartite motif-containing protein 2	*Trim2*	1.48	Up	258530000	174747500
Tripartite motif-containing protein 3	*Trim3*	1.09	Up	175020000	160805000
Tripeptidyl-peptidase 1	*Tpp1*	1.01	Down	746463333.3	751680000
Tripeptidyl-peptidase 2	*Tpp2*	1.01	Up	699282500	692732500
tRNA-dihydrouridine(47) synthase [NAD(P)(+)]-like	*Dus3l*	1.22	Up	145213333.3	119230000
tRNA-splicing ligase RtcB homolog	*Rtcb*	1.03	Up	3169125000	3072075000
Trophoblast glycoprotein	*Tpbg*	1.72	Down	49395000	84988000
Tropomodulin-2	*Tmod2*	1.06	Down	551965000	584470000
Tropomyosin alpha-3 chain	*Tpm3*	1.14	Up	360455000	316755000
Tryptophan--tRNA ligase, cytoplasmic;T1-TrpRS;T2-TrpRS	*Wars*	1.08	Down	1040112500	1120125000

Tubulin alpha-1A chain;Tubulin alpha-3 chain	*Tuba1a;Tuba3a*	1.07	Up	1.86443E+11	1.7469E+11
Tubulin alpha-1B chain	*Tuba1b*	1.03	Up	17442000000	16899750000
Tubulin alpha-4A chain	*Tuba4a*	1.05	Up	7466925000	7129725000
Tubulin beta-2B chain	*Tubb2b*	1.04	Up	41901750000	40449750000
Tubulin beta-3 chain	*Tubb3*	1.08	Down	9903000000	10717400000
Tubulin beta-4B chain	*Tubb4b*	1.04	Up	1.2388E+11	1.1906E+11
Tubulin beta-5 chain	*Tubb5*	1.11	Up	19009750000	17191750000
Tubulin gamma-1 chain	*Tubg1*	1.26	Down	40820000	51503000
Tubulin polymerization-promoting protein family member 3	*Tppp3*	1.57	Down	350595000	549615000
Tubulin-specific chaperone A	*Tbca*	1.12	Up	163950000	146713333.3
Tudor domain-containing protein 7	*Tdrd7*	1.21	Down	126440000	152600000
Tumor protein p63-regulated gene 1-like protein	*Tprg1l*	1.03	Down	65446500	67715000
Tumor susceptibility gene 101 protein	*Tsg101*	1.11	Up	299720000	269277500
Twinfilin-1	*Twf1*	1.07	Up	338676666.7	318000000
Type 2 phosphatidylinositol 4,5-bisphosphate 4-phosphatase	*Tmem55a*	1.30	Down	100459000	130140000
Type I inositol 3,4-bisphosphate 4-phosphatase	*Inpp4a*	1.05	Down	248762500	261187500
Tyrosine 3-monooxygenase	*Th*	1.87	Down	54232000	101462000
Tyrosine-protein phosphatase non-receptor type 11	*Ptpn11*	1.06	Down	1067007500	1130125000
Tyrosine-protein phosphatase non-receptor type 23	*Ptpn23*	1.08	Down	164062500	177257500
Tyrosine-protein phosphatase non-receptor type substrate 1	*Sirpa*	1.30	Up	262620000	201465000
Tyrosine--tRNA ligase, cytoplasmic;Tyrosine--tRNA ligase, cytoplasmic, N-terminally processed	*Yars*	1.16	Down	1192372500	1388525000
Tyrosyl-DNA phosphodiesterase 1	*Tdp1*	1.45	Down	9693800	14101000
U1 small nuclear ribonucleoprotein C	*Snrpc*	1.20	Down	348402500	418890000
U4/U6.U5 tri-snRNP-associated protein 1	*Sart1*	1.05	Down	110525000	116420000
U5 small nuclear ribonucleoprotein 200 kDa helicase	*Snrnp200*	1.09	Down	2106350000	2303400000
Ubiquilin-1	*Ubqln1*	1.04	Down	431950000	450232500
Ubiquitin carboxyl-terminal hydrolase 10	*Usp10*	1.11	Up	172682500	155995000
Ubiquitin carboxyl-terminal hydrolase 19	*Usp19*	1.49	Down	66728000	99718000
Ubiquitin carboxyl-terminal hydrolase 7	*Usp7*	1.04	Down	1102022500	1141675000
Ubiquitin carboxyl-terminal hydrolase isozyme L1	*Uchl1*	1.04	Down	9070375000	9404700000
Ubiquitin carboxyl-terminal hydrolase isozyme L3	*Uchl3*	1.27	Down	245136666.7	310247500
Ubiquitin fusion degradation protein 1 homolog	*Ufd1l*	1.08	Down	216415000	232760000
Ubiquitin thioesterase OTUB1	*Otub1*	1.03	Down	2404850000	2478925000
Ubiquitin-40S ribosomal protein S27a;Ubiquitin;40S ribosomal protein S27a;Ubiquitin-60S ribosomal protein L40;Ubiquitin;60S ribosomal protein L40;Polyubiquitin-B;Ubiquitin;Polyubiquitin-C;Ubiquitin;Ubiquitin-related	*Rps27a;Uba52;Ubb;Ubc*	1.12	Down	13056500000	14682500000
Ubiquitin-conjugating enzyme E2 D2	*Ube2d2*	1.07	Up	822830000	769910000
Ubiquitin-conjugating enzyme E2 N	*Ube2n*	1.00	Down	2985775000	2995875000
Ubiquitin-conjugating enzyme E2 variant 2	*Ube2v2*	1.08	Up	1087542500	1010097500
Ubiquitin-conjugating enzyme E2 Z	*Ube2z*	1.37	Up	218840000	159480000
Ubiquitin-fold modifier-conjugating enzyme 1	*Ufc1*	1.02	Up	250720000	246913333.3
Ubiquitin-like domain-containing CTD phosphatase 1	*Ublcp1*	1.01	Up	498237500	491237500
Ubiquitin-like modifier-activating enzyme 1	*Uba1*	1.01	Up	15074000000	14944000000

Ubiquitin-like protein 4A	*Ubl4a*	1.19	Down	158355000	188377500
Ubiquitin-like-conjugating enzyme ATG3	*Atg3*	1.09	Up	138110000	126900000
UBX domain-containing protein 1	*Ubxn1*	1.13	Up	232557500	205085000
UBX domain-containing protein 4	*Ubxn4*	1.05	Down	169940000	179250000
UDP-glucose 4-epimerase	*Gale*	1.22	Up	420020000	345370000
UDP-glucose 6-dehydrogenase	*Ugdh*	1.42	Up	110595000	77697000
UDP-glucose:glycoprotein glucosyltransferase 1	*Uggt1*	1.01	Down	921495000	933007500
UDP-N-acetylglucosamine--peptide N-acetylglucosaminyltransferase 110 kDa subunit	*Ogt*	1.13	Up	653770000	579725000
UMP-CMP kinase	*Cmpk1*	1.01	Down	1187475000	1200650000
Uncharacterized protein C12orf43 homolog	*N/A*	1.06	Down	113100000	120165000
Uncharacterized protein C6orf136 homolog	*N/A*	1.01	Down	53788000	54112000
Unconventional myosin-Ib	*Myo1b*	1.22	Up	133962500	109612500
Unconventional myosin-Va	*Myo5a*	1.04	Up	1928725000	1853900000
UPF0160 protein MYG1, mitochondrial	*Myg1*	1.09	Up	214400000	196640000
UPF0562 protein C7orf55 homolog	*LOC108099353*	1.14	Down	54016000	61316333.33
UPF0587 protein C1orf123 homolog	*N/A*	1.14	Up	65202000	57350000
UPF0598 protein C8orf82 homolog	*N/A*	1.20	Up	115908750	96206000
UPF0696 protein C11orf68 homolog	*Bles03*	1.11	Down	187760000	207790000
Up-regulated during skeletal muscle growth protein 5	*Usmg5*	1.09	Down	1394642500	1522575000
Urea transporter 1	*Slc14a1*	1.75	Up	367257500	209727500
UV excision repair protein RAD23 homolog B	*Rad23b*	1.03	Up	305842500	297020000
Vacuolar protein sorting-associated protein 26A	*Vps26a*	1.19	Up	138732500	116463250
Vacuolar protein sorting-associated protein 28 homolog	*Vps28*	1.29	Up	156110000	121356666.7
Vacuolar protein sorting-associated protein 29	*Vps29*	1.08	Up	577587500	532382500
Vacuolar protein sorting-associated protein 33A	*Vps33a*	1.40	Down	64095000	89625000
Vacuolar protein sorting-associated protein 33B	*Vps33b*	1.28	Up	60992000	47815000
Vacuolar protein sorting-associated protein 45	*Vps45*	1.16	Up	305176666.7	264075000
Vacuolar protein sorting-associated protein 4A	*Vps4a*	1.01	Down	316662500	319947500
Vacuole membrane protein 1	*Vmp1*	1.24	Up	101030000	81744000
Valine--tRNA ligase	*Vars*	1.07	Up	1804525000	1690300000
Very long-chain specific acyl-CoA dehydrogenase, mitochondrial	*Acadvl*	1.11	Down	238315000	265570000
Very-long-chain 3-oxoacyl-CoA reductase	*Hsd17b12*	1.51	Up	283850000	187745000
Very-long-chain enoyl-CoA reductase	*Tecr*	1.09	Up	898845000	822965000
Vesicle-associated membrane protein 2	*Vamp2*	1.22	Up	9263825000	7604325000
Vesicle-associated membrane protein-associated protein A	*Vapa*	1.14	Down	2392500000	2715575000
Vesicle-associated membrane protein-associated protein B	*Vapb*	1.05	Up	1230507500	1171680000
Vesicle-fusing ATPase	*Nsf*	1.06	Up	36787000000	34569000000
Vesicle-trafficking protein SEC22b	*Sec22b*	1.11	Up	637600000	573762500
Vesicular acetylcholine transporter	*Slc18a3*	1.50	Up	129949666.7	86501500
Vesicular glutamate transporter 1	*Slc17a7*	1.07	Down	1148925000	1225775000
Vesicular inhibitory amino acid transporter	*Slc32a1*	1.02	Down	3696300000	3781900000
Vigilin	*Hdlbp*	1.01	Up	949707500	944950000
Vimentin	*Vim*	1.01	Up	55972000000	55646250000
Vinculin	*Vcl*	1.07	Up	1393850000	1300425000
Visinin-like protein 1	*Vsnl1*	1.01	Up	1012837500	1001747500

Vitamin K epoxide reductase complex subunit 1-like protein 1	*Vkorc1l1*	1.05	Up	239882500	227992500
Voltage-dependent anion-selective channel protein 1	*Vdac1*	1.00	Up	36267500000	36119000000
Voltage-dependent anion-selective channel protein 2	*Vdac2*	1.06	Up	10400425000	9768250000
Voltage-dependent anion-selective channel protein 3	*Vdac3*	1.05	Up	9989125000	9532000000
Voltage-dependent calcium channel subunit alpha-2/delta-1;Voltage-dependent calcium channel subunit alpha-2-1;Voltage-dependent calcium channel subunit delta-1	*Cacna2d1*	1.12	Up	579695000	518835000
Voltage-dependent calcium channel subunit alpha-2/delta-2;Voltage-dependent calcium channel subunit alpha-2-2;Voltage-dependent calcium channel subunit delta-2	*Cacna2d2*	1.17	Up	1132682500	967927500
Voltage-gated potassium channel subunit beta-2	*Kcnab2*	1.13	Down	125685000	142492500
V-type proton ATPase 116 kDa subunit a isoform 1	*Atp6v0a1*	1.11	Up	12073750000	10919000000
V-type proton ATPase 16 kDa proteolipid subunit	*Atp6v0c*	1.04	Down	2727625000	2846300000
V-type proton ATPase subunit B, brain isoform	*Atp6v1b2*	1.08	Up	15493000000	14403750000
V-type proton ATPase subunit C 1	*Atp6v1c1*	1.02	Up	4043350000	3958725000
V-type proton ATPase subunit E 1	*Atp6v1e1*	1.24	Down	5877350000	7277675000
V-type proton ATPase subunit F	*Atp6v1f*	1.07	Up	418135000	392235000
V-type proton ATPase subunit S1	*Atp6ap1*	1.02	Down	501245000	510790000
WD repeat-containing protein 1	*Wdr1*	1.04	Up	7641625000	7334450000
WD repeat-containing protein 44	*Wdr44*	1.28	Down	62067000	79526750
WD repeat-containing protein 5	*Wdr5*	1.02	Down	383645000	389910000
WD repeat-containing protein 6	*Wdr6*	1.92	Down	10836000	20762000
WD repeat-containing protein 61;WD repeat-containing protein 61, N-terminally processed	*Wdr61*	1.09	Down	310337500	339242500
WD repeat-containing protein 7	*Wdr7*	1.07	Up	1715100000	1609525000
WD repeat-containing protein 89	*Wdr89*	1.13	Down	104470000	118205000
WD40 repeat-containing protein SMU1;WD40 repeat-containing protein SMU1, N-terminally processed	*Smu1*	1.37	Down	448995000	615107500
Wiskott-Aldrich syndrome protein family member 1	*Wasf1*	1.09	Down	75764000	82269750
WW domain-binding protein 11	*Wbp11*	1.11	Up	122970000	110304000
WW domain-binding protein 2	*Wbp2*	1.00	Up	661360000	659000000
Xaa-Pro aminopeptidase 1;Xaa-Pro aminopeptidase 1, N-terminally processed	*Xpnpep1*	1.06	Up	829977500	784485000
Xaa-Pro dipeptidase	*Pepd*	1.07	Up	886640000	828695000
Y-box-binding protein 3	*Ybx3*	1.03	Down	51431000	53194000
Zinc finger protein 22	*Znf22*	1.47	Up	45265666.67	30708000
Zinc finger protein 652	*Znf652*	1.63	Down	7768200	12693000
Zinc finger Ran-binding domain-containing protein 2	*Zranb2*	1.02	Up	79320000	77915000
Zinc finger RNA-binding protein	*Zfr*	1.27	Down	271942500	344887500
Zinc phosphodiesterase ELAC protein 2	*Elac2*	1.01	Up	36486000	36055000
ZW10 interactor	*Zwint*	1.08	Up	208970000	194270000
